# Degradation of neurodegenerative disease-associated TDP-43 aggregates and oligomers via a proteolysis-targeting chimera

**DOI:** 10.1186/s12929-023-00921-7

**Published:** 2023-04-26

**Authors:** Yu-Ling Tseng, Po-Chao Lu, Chi-Chang Lee, Ruei-Yu He, Yung-An Huang, Yin-Chen Tseng, Ting-Jen Rachel Cheng, Joseph Jen-Tse Huang, Jim-Min Fang

**Affiliations:** 1 Department of Chemistry, National Taiwan University, Taipei, 106 Taiwan; 2 Institute of Chemistry, Academia Sinica, Taipei, 115 Taiwan; 3 Chemical Biology and Molecular Biophysics, Taiwan International Graduate Program, Academia Sinica, Taipei, 115 Taiwan; 4 Sustainable Chemical Science and Technology, Taiwan International Graduate Program, Academia Sinica, Taipei, 115 Taiwan; 5 Department and Graduate Institute of Pharmacology, National Taiwan University, Taipei, 100 Taiwan; 6 The Genomics Research Center, Academia Sinica, Taipei, 115 Taiwan; 7 Department of Applied Chemistry, National Chiayi University, Chiayi City, 600 Taiwan; 8 Neuroscience Program of Academia Sinica, Academia Sinica, Taipei, 115 Taiwan

**Keywords:** Neurodegenerative diseases, Amyotrophic lateral sclerosis, TDP-43 cytotoxicity, Aggregate and oligomer, PROTACs, Protein degradation, Transgenic *C. elegans*

## Abstract

**Background:**

Amyotrophic lateral sclerosis (ALS) associated with TAR DNA-binding protein 43 (TDP-43) aggregation has been considered as a lethal and progressive motor neuron disease. Recent studies have shown that both C-terminal TDP-43 (C-TDP-43) aggregates and oligomers were neurotoxic and pathologic agents in ALS and frontotemporal lobar degeneration (FTLD). However, misfolding protein has long been considered as an undruggable target by applying conventional inhibitors, agonists, or antagonists. To provide this unmet medical need, we aim to degrade these misfolding proteins by designing a series of proteolysis targeting chimeras (PROTACs) against C-TDP-43.

**Methods:**

By applying filter trap assay, western blotting, and microscopy imaging, the degradation efficiency of C-TDP-43 aggregates was studied in Neuro-2a cells overexpressing eGFP-C-TDP-43 or mCherry-C-TDP-43. The cell viability was characterized by alarmarBlue assay. The beneficial and disaggregating effects of TDP-43 PROTAC were examined with the YFP-C-TDP-43 transgenic *C. elegans* by motility assay and confocal microscopy. The impact of TDP-43 PROTAC on C-TDP-43 oligomeric intermediates was monitored by fluorescence lifetime imaging microscopy and size exclusion chromatography in the Neuro-2a cells co-expressing eGFP-C-TDP-43 and mCherry-C-TDP-43.

**Results:**

Four PROTACs with different linker lengths were synthesized and characterized. Among these chimeras, PROTAC **2** decreased C-TDP-43 aggregates and relieved C-TDP-43-induced cytotoxicity in Neuro-2a cells without affecting endogenous TDP-43. We showed that PROTAC **2** bound to C-TDP-43 aggregates and E3 ligase to initiate ubiquitination and proteolytic degradation. By applying advanced microscopy, it was further shown that PROTAC **2** decreased the compactness and population of C-TDP-43 oligomers. In addition to cellular model, PROTAC **2** also improved the motility of transgenic *C. elegans* by reducing the C-TDP-43 aggregates in the nervous system.

**Conclusions:**

Our study demonstrated the dual-targeting capacity of the newly-designed PROTAC **2** against both C-TDP-43 aggregates and oligomers to reduce their neurotoxicity, which shed light on the potential drug development for ALS as well as other neurodegenerative diseases.

**Supplementary Information:**

The online version contains supplementary material available at 10.1186/s12929-023-00921-7.

## Background

With the increase in the aging population, the misfolded proteinaceous agents associated with neurodegenerative diseases have drawn increasing attention [1, 2]. The major symptom of these diseases is severe motility and/or cognitive dysfunctions resulting from the damage of neuron cells. Clinicopathologically, the regional misfolding protein aggregate within the cells is an important hallmark [3]. Since misfolding protein aggregates are considered as an “undruggable” target in terms of the conventional drugs, clinical trial experience for these diseases is still challenging. Currently, therapeutic agents can only provide temporary symptomatic relief rather than reversing disease progression [4], reflecting the difficulty in drug discovery. In addition, despite transgenic animal models have enormously benefitted the preclinical trial in drug development against neurodegenerative diseases, the phenotypical similarities and differences between animal models and human race should be taken into consideration [5, 6]. Furthermore, delivery of drugs to central nervous system (CNS) is difficult as blood–brain barrier (BBB) restricts most of the drugs to reach the putatively therapeutic targets [4]. Therefore, developing new strategies against neurodegenerative diseases is urgently needed.

Recently, proteolysis targeting chimeras (PROTACs) have received increasing attention due to their potential abilities to induce targeted protein degradation including the neurodegenerative disease associated proteins [7–17]. Structurally, PROTACs are heterobifunctional molecules consisting of an E3-ligase recruiting moiety and a binding ligand of targeted protein, which are joined by an appropriate linker. Upon forming a ternary complex of (E3 ligase)–PROTAC–(targeted protein), the induced proximity can facilitate the transfer of ubiquitins to target protein (namely polyubiquitination), and render the protein for degradation by proteasome. Unlike the traditional inhibitor that requires a stoichiometric amount to suppress the activity of target protein, a selective PROTAC in substoichiometric amount can eliminate all the levels of target protein [13, 18]. The catalytic action mode of PROTAC allows it to perform multiple rounds of ubiquitination on target protein, thus a promise providing efficiency in protein degradation with decreased drug concentration to avoid adverse side effect. However, a general structural design of efficient PROTACs does not exist due to lots of variables. The choice of binding ligands to recruit E3-ligase and target protein would influence the degradation profiles of the PROTACs [7]. Though more than 600 E3-ligases are encoded in mammalian genome [19], only a few E3-ligases, including murine double minute 2 (MDM2), cellular inhibitor of apoptosis protein (cIAP), Von Hippel-Lindau (VHL) and cereblon (CRBN) complex, with strong binding ligands are commonly applied in PROTACs [20]. In addition, the chemical composition and chain length of the linker are critical factors affecting the efficiency of the PROTAC compound [18, 21–23]. So far, optimization of PROTAC molecules is still done on a case-by-case basis [24].

Nowadays, PROTACs have been developed to degrade a wide range of proteins, such as androgen receptor [25], bromodomain-containing protein 4 [26–28], BCR-ABL tyrosine kinase [7], and E3-ligase self-degrader [22]. Recently, PROTACs were also applied to examine on neurodegenerative diseases including Alzheimer's disease [29–31], Huntington's disease, [9, 17] and Parkinson's disease [32]. Though considerable effort has been devoted, there is yet no effective PROTAC for the treatment of amyotrophic lateral sclerosis (ALS) [33]. ALS is a progressive motor neuron disease leading to paralysis and eventually death. Until now, riluzole (inhibitor of glutamic acid release) and edaravone (free radical scavenger) are the two drugs approved by the FDA to relief the symptoms of ALS [34]. In 2006, the C-terminal TAR DNA-binding protein (referred as C-TDP-43 hereafter) was identified as the major component in the inclusions of ALS and frontotemporal lobar degeneration (FTLD) patients [35]. Later on, the accumulation of TDP-43 aggregates was also found in the central nervous system of different neurodegenerative diseases [36]. TDP-43 is an ubiquitously expressed DNA/RNA binding protein implicated in gene transcription, pre-mRNA splicing, and translational regulation [37]. Later studies have further disclosed that C-TDP-43 protein and some peptide fragments of C-TDP-43 form toxic aggregates with amyloid properties [38–44]. Apart from the C-TDP-43 aggregates, cumulative evidence has also argued that TDP-43 oligomers played an important role in ALS and FTLD [45, 46]. Since both C-TDP-43 aggregates and oligomers are neurotoxic and pathologic agents in TDP-43 proteinopathy, they are all included in this study.

**Fig. 1:**
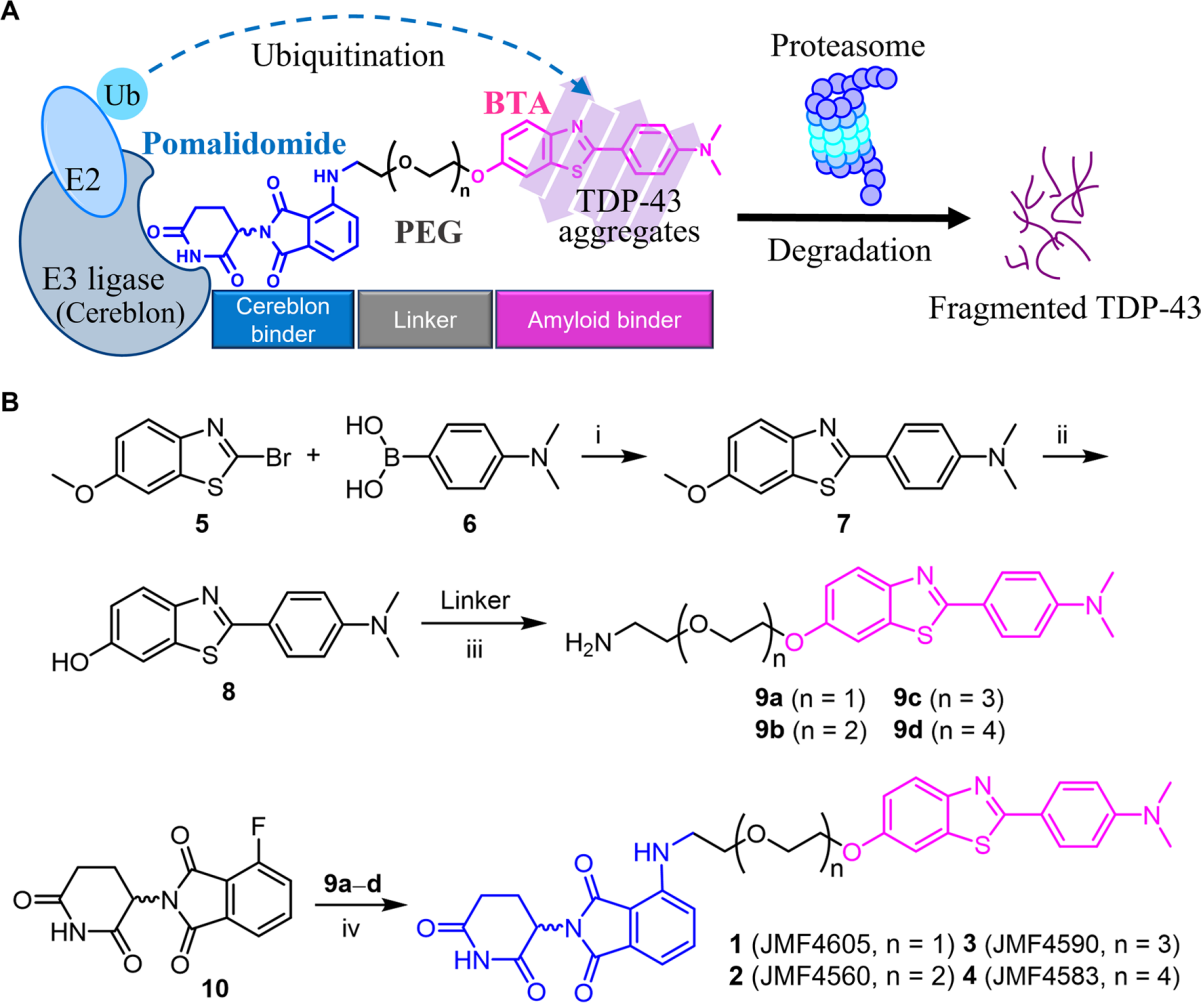
Mechanism for degradation of TDP-43 aggregates by PROTAC molecules. **A** The PROTAC binds E3 ligase and TDP-43 aggregates simultaneously to facilitate the transfer of ubiquitins to TDP-43 aggregates. As ubiquitin chains on TDP-43 aggregates are recognized by proteasome, TDP-43 aggregates are degraded. BTA: benzothiazole-aniline derivative. **B** The synthetic process of PROTACs **1**–**4**. *Reagents and reaction conditions*: (i) Pd(dppf)Cl_2_, K_2_CO_3_, DMF, 80 °C, 18 h; 60%. (ii) BBr_3_, CH_2_Cl_2_, 0 °C, 20 h; 98%. (iii) For **9a**, N_3_CH_2_CH_2_(OCH_2_CH_2_)OMs, K_2_CO_3_, DMF, 80 °C, 21 h; then PPh_3_, THF, rt, 24 h; 50% overall yield. (iv) *i*-Pr_2_NEt, NMP, 90 °C, 16–18 h; 32%. For the synthesis of **9b**–**9d**, see experimental section

To provide a proof-of-concept examination, we developed “TDP-43 PROTAC” as a novel therapeutic strategy for reducing the C-TDP-43 cytotoxicity in ALS and other TDP-43 proteinopathy (Fig. 1A). A TDP-43 PROTAC simultaneously binds C-TDP-43 and the engaging E3-ligase, and thus facilitates tagging ubiquitins to C-TDP-43 for further degradation by proteasomes. Among a number of synthetic PROTACs with variable composition of linkers, compounds **1–4** (JMF4605, JMF4560, JMF4590 and JMF4583 in Fig. 1) with different lengths of ethylene glycol linkers (n = 1–4) showed proteolysis activity on misfolded C-TDP-43. Our studies further demonstrated that the misfolded C-TDP-43 rather than endogenous TDP-43 could be selectively degraded. In addition to reducing C-TDP-43 toxicity in mammalian cultured cells, PROTAC **2** (JMF4560) also rescued the C-TDP-43-mediated motility defects in nematode *C. elegans*.

## Methods

### General

All the reagents and solvents were reagent grade and used as purchased without further purification unless indicated otherwise. All solvents were anhydrous grade unless indicated otherwise. Dichloromethane (CH_2_Cl_2_) was distilled from CaH_2_, and tetrahydrofuran (THF) was distilled from sodium. All nonaqueous reactions were performed in oven-dried glassware under a slightly positive pressure of argon unless otherwise noted. Reactions were magnetically stirred and monitored by thin-layer chromatography on silica gel using phosphomolybdic acid (PMA), *p*-anisaldehyde, KMnO_4_, ninhydrin or iodine vapor as visualizing agent. Flash chromatography was performed on silica gel of 60–200 μm particle size. Yields are reported for spectroscopically pure compounds. Melting points were recorded on a Yanaco micro apparatus or Electrothermal MEL-TEMP 1101D apparatus in open capillaries and are not corrected. Infrared (IR) spectra were recorded on Thermo Scientific Nicolet is-5 FT-IR spectrometer. Nuclear magnetic resonance (NMR) spectra were obtained on Bruker AVIII 500 (500 MHz) and Bruker AVIII 400 (400 MHz) spectrometers. Chemical shifts are given in δ values relative to tetramethylsilane (TMS, δ_H_ = 0). Internal standards were CHCl_3_ (δ_H_ = 7.24), CDCl_3_ (δ_C_ = 77.0, central line of the triplet), CD_2_HOD (δ_H_ = 3.31), CD_3_OD (δ_C_ = 49.15), (H_3_C)_2_SO (DMSO, δ_H_ = 2.50), or (CHD_2_)_2_SO (DMSO-*d*_6_, δ_C_ = 39.5). The splitting patterns are reported as s (singlet), d (doublet), t (triplet), q (quartet), m (multiplet), dd (double of doublets), td (triple of doublets) and br (broad). Coupling constants (*J*) are given in Hz. Electrospray ionization high-resolution mass spectra (ESI-HRMS) were recorded on a Bruker Daltonics BioTOF III high-resolution mass spectrometer. UV–Vis absorption spectra were recorded on a PerkinElmer Lambda 35 spectrometer. Fluorescence spectra were recorded on an AMINCO-Bowman Series 2 luminescence spectrometer.

New compounds were characterized by their physical and spectroscopic properties (mp, IR, ESI − MS, ^1^H, ^13^C and ^19^F NMR). All compounds are > 95% pure by HPLC analysis.

### Representative synthetic procedures of PROTACs

#### 2-(4-(Dimethylamino)phenyl)benzo[*d*]thiazol-6-ol (8) [47]

According to the previously reported procedure, 2-amino-6-methoxybenzothiazole (3 g, 16.6 mmol) was stirred with copper bromide (5.58 g, 25 mmol) and *tert*-butyl nitrite (2.96 mL, 25 mmol) in anhydrous acetonitrile (75 mL) at 65 °C for 1.5 h to give 2-bromo-6-methoxybenzo[*d*]thiazole (**5**) [48] (3.54 g, 14.5 mmol, 87% yield). C_8_H_6_BrNOS; brown solid; mp 47.5–49.0 °C.

A mixture of bromo compound **5** (100 mg, 0.41 mmol), 4-(dimethylamino)phenylboronic acid (82 mg, 0.50 mmol), K_2_CO_3_ (373 mg, 2.7 mmol) and Pd(dppf)Cl_2_CH_2_Cl_2_ (22 mg, 0.027 mmol) in anhydrous DMF (2 mL) was stirred at 80 °C for 23 h under an atmosphere of argon to afford **4-**(6-methoxybenzo[*d*]thiazol-2-yl)-*N,N*-dimethylaniline (**7**) [49]. C_16_H_16_N_2_OS; brown solid; mp 179.5–181.5 °C.

A solution of compound **7** (1.4 g, 4.9 mmol) in anhydrous CH_2_Cl_2_ (75 mL) was added BBr_3_ (5.61 mL, 60 mmol) dropwise at 0 °C under an atmosphere of argon. The mixture was stirred at room temperature for 24 h. The reaction was quenched by addition of H_2_O, and the solution was adjusted to pH 6–7 by addition of NaOH_(aq)_. The orange precipitate was collected by vacuum filtration. The crude product was recrystallized from MeOH/Et_2_O to give pure compound **8** (1.3 g, 98% yield). C_15_H_14_N_2_OS; orange solid; mp 227.0–228.0 °C.

#### 4-(6-(2-(2-Aminoethoxy)ethoxy)benzo[*d*]thiazol-2-yl)-*N*,*N*-dimethylaniline (9a)

To a solution of compound **8** (100 mg, 0.37 mmol) in anhydrous DMF (3 mL) was added K_2_CO_3_ (102 mg, 0.74 mmol). The mixture was stirred for 30 min at room temperature, and then treated with 2-(2-azidoethoxy)ethyl methanesulfonate [50] (116 mg, 0.56 mmol) at 80 °C for 21 h. The mixture was cooled, and concentrated under reduced pressure. The mixture was extracted with EtOAc and H_2_O. The organic phase was dried over MgSO_4_, filtered, concentrated under reduced pressure, and purified by flash chromatography on a silica gel column with elution of CH_2_Cl_2_ to give the alkylation product, 4-(6-(2-(2-azidoethoxy)ethoxy)benzo[*d*]thiazol-2-yl)-*N*,*N*-dimethylaniline compound (**S2a**) (66 mg, 51% yield). C_19_H_21_N_5_O_2_S; yellow solid; mp 96.0–97.0 °C.

A solution of azido compound **S2a** (202 mg, 0.53 mmol) in THF (3 mL) was stirred with PPh_3_ (415 mg, 1.6 mmol) and H_2_O (30 µL, 1.6 mmol) at room temperature for 24 h. The mixture was concentrated under reduced pressure, and purified by flash chromatography on a silica gel column with elution of CH_2_Cl_2_/MeOH (10:1) to give the amino compound **9a** (195 mg, 99% yield). C_19_H_23_N_3_O_2_S; yellow solid; mp 66.5–67.5 °C; TLC (CH_2_Cl_2_/MeOH = 7:1) *R*_*f*_ = 0.13; IR ν_max_ (neat) 3358, 3190, 2919, 2849, 1659, 1653, 1634, 1470, 1423, 1264, 828 cm^–1^; ^1^H NMR (400 MHz, CDCl_3_) δ 7.83 (d, *J* = 8.8 Hz, 2 H), 7.80 (d, *J* = 8.9 Hz, 1 H), 7.26 (d, *J* = 2.1 Hz, 1 H), 7.00 (dd, *J* = 8.9, 2.1 Hz, 1 H), 6.65 (d, *J* = 8.8 Hz, 2 H), 4.10 (t, *J* = 4.4 Hz, 2 H), 3.77 (t, *J* = 4.4 Hz, 2 H), 3.52 (t, *J* = 4.9 Hz, 2 H), 2.95 (s, 6 H), 2.83 (s, 2 H), 1.91 (br, 2 H); ^13^C NMR (100 MHz, CDCl_3_) δ 166.4, 156.0, 151.7, 148.9, 135.6, 128.3 (2 ×), 122.5, 121.3, 115.2, 111.5 (2 ×), 105.2, 73.3, 69.3, 67.9, 41.5, 39.9 (2 ×). ESI-HRMS calcd for C_19_H_24_N_3_O_2_S: 358.1584, found: *m/z* 358.1570 [M + H]^+^.

#### 4-((2-(2-((2-(4-(Dimethylamino)phenyl)benzo[*d*]thiazol-6-yl)oxy)ethoxy)ethyl)amino)-2-(2,6-dioxopiperidin-3-yl)isoindoline-1,3-dione (1)

A mixture of 3-fluorophthalic anhydride (100 mg, 0.6 mmol), 2,6-dioxopiperidin-3-amine hydrochloride (99 mg, 0.6 mmol) and NaOAc3H_2_O (98 mg, 0.72 mmol) in AcOH (3 mL) was heated under reflux for 12 h. The mixture was concentrated under reduced pressure, and purified by flash chromatography on a silica gel column with elution of CH_2_Cl_2_/MeOH (100:1) to give 2-(2,6-dioxopiperidin-3-yl)-4-fluoroisoindoline-1,3-dione (**10**) [51] (155 mg, 93% yield). The purity of compound **10** was 98.9% as shown by HPLC on a silica column (Dikma, 10 × 250 mm, 10 μm particle size), elution: EtOAc/hexane = 4:1 at a flow rate of 3.0 mL/min, *t*_R_ = 8.1 min. C_13_H_9_FN_2_O_4_; white solid; mp 255.5–257.0 °C.

A mixture of compound **9a** (181 mg, 0.66 mmol), compound **10** (180 mg, 0.50 mmol) and diisopropylethylamine (DIPEA) (180 µL, 1.01 mmol) in 1-methyl-2-pyrrolidone (NMP) (2.5 mL) was stirred at 90 °C for 18 h. The mixture was extracted with EtOAc and H_2_O. The combined organic phase was dried over MgSO_4_, filtered, concentrated under reduced pressure, and purified by flash chromatography on a silica gel column with elution of EtOAc/CH_2_Cl_2_ (1:2) to give the desired compound **1** (100 mg, 32% yield). The purity of compound **1** was 96.4% as shown by HPLC on a silica column (Dikma, 10 × 250 mm, 10 μm particle size), elution: EtOAc/hexane = 3:1 at a flow rate of 3.0 mL/min, *t*_R_ = 12.2 min. C_32_H_31_N_5_O_6_S; yellow solid; mp 157.0–158.0 °C; TLC (EtOAc/ CH_2_Cl_2_ = 1:2)* R*_*f*_ = 0.63; IR ν_max_ (neat) 3359, 3182, 2919, 2849, 1699, 1695, 1657, 1557, 1538, 1471 cm^–1^; ^1^H NMR (400 MHz, CDCl_3_) δ 8.15 (s, 1 H), 7.97–7.80 (m, 3 H), 7.44 (t, *J* = 7.8 Hz, 1 H), 7.30 (s, 1 H), 7.10–7.00 (m, 2 H), 6.90 (d, *J* = 8.5 Hz, 1 H), 6.74 (d, *J* = 8.5 Hz, 2 H), 6.50 (s, 1 H), 4.86 (q, *J* = 5.4 Hz, 1 H), 4.19 (t, *J* = 4.3 Hz, 2 H), 3.87 (t, *J* = 4.3, 2 H), 3.79 (t, *J* = 5.1 Hz, 2 H), 3.48 (q, *J* = 5.1 Hz, 2 H), 3.03 (s, 6 H), 2.86–2.67 (m, 3 H), 2.08–2.00 (m, 1 H); ^13^C NMR (125 MHz, DMSO-*d*_*6*_) δ 172.9, 170.2, 169.0, 167.4, 165.5, 156.0, 151.9, 148.3, 146.5, 136.3, 135.2, 132.1, 128.2 (2 ×), 122.4, 120.5, 117.5, 115.7, 112.0 (2 ×), 110.8, 109.3, 105.8, 69.0, 68.8, 67.8, 48.6, 41.7 (2 ×), 31.1, 22.2, 18.6. ESI-HRMS calcd for C_32_H_32_N_5_O_6_S: 614.2068, found: *m/z* 614.2031 [M + H]^+^.

#### 4-((2-(2-(2-((2-(4-(Dimethylamino)phenyl)benzo[*d*]thiazol-6-yl)oxy)ethoxy)ethoxy)ethyl)amino)-2-(2,6-dioxopiperidin-3-yl)isoindoline-1,3-dione (2)

By a procedure similar to that for compound **1**, the substitution reaction of **10** (74 mg, 0.26 mmol) with **9b** (54 mg, 0.13 mmol) gave a crude product, which was purified by flash chromatography on a silica gel column with elution of EtOAc/CH_2_Cl_2_ (1:1) to give the desired compound **2** (46 mg, 52% yield). The purity of compound **2** was 97.0% as shown by HPLC on a silica column (Dikma, 10 × 250 mm, 10 μm particle size), elution: EtOAc/hexane = 3:1 at a flow rate of 3.0 mL/min, *t*_R_ = 17.9 min. C_34_H_35_N_5_O_7_S; yellow solid; mp 128.0–129.0 °C; TLC (EtOAc/ CH_2_Cl_2_ = 1:1) *R*_*f*_ = 0.5; IR ν_max_ (neat) 3357, 3197, 2920, 2849, 1653, 1632, 1471 cm^–1^; ^1^H NMR (400 MHz, CDCl_3_) δ 8.35 (s, 1 H), 7.88 (d, *J* = 8.8 Hz, 2 H), 7.82 (d, *J* = 8.9 Hz, 1 H), 7.41 (t, *J* = 7.8 Hz, 1 H), 7.29 (d, *J* = 2.4 Hz, 1 H), 7.01–6.99 (m, 2 H), 6.84 (d, *J* = 8.5 Hz, 1 H), 6.71 (d, *J* = 8.8 Hz, 2 H), 6.44 (t, *J* = 5.4 Hz, 1 H), 4.85 (dd, *J* = 12.1, 5.3 Hz, 1 H), 4.15 (t, *J* = 4.7 Hz, 2 H), 3.86 (t, *J* = 4.7 Hz, 2 H), 3.78–3.64 (m, 6 H), 3.40 (q, *J* = 5.4 Hz, 2 H), 3.01 (s, 6 H), 2.87–2.59 (m, 3 H), 2.09–2.00 (m, 1 H); ^13^C NMR (100 MHz, CHCl_3_) δ 171.1, 169.2, 168.4, 167.6, 166.6, 156.2, 151.9, 149.0, 146.7, 136.0, 135.6, 132.4, 128.5 (2 ×), 122.6, 121.5, 116.7, 115.4, 111.7 (2 ×), 111.6, 110.2, 105.4, 70.9, 70.7, 69.8, 69.5, 68.1, 48.8, 42.3, 40.14 (2 ×), 31.3, 22.7. ESI-HRMS calcd for C_34_H_36_N_5_O_7_S: 658.2330, found: *m/z* 658.2307 [M + H]^+^.

#### 4-((2-(2-(2-(2-((2-(4-(Dimethylamino)phenyl)benzo[*d*]thiazol-6yl)oxy)ethoxy)ethoxy)ethoxy)ethyl)amino)-2-(2,6-dioxopiperidin-3-yl)isoindoline-1,3-dione (3)

By a procedure similar to that for compound **1**, the substitution reaction of **10** (88 mg, 0.32 mmol) with **9c** (110 mg, 0.25 mmol) gave a crude product, which was purified by flash chromatography on a silica gel column with elution of EtOAc/CH_2_Cl_2_ (1:1) to give the desired compound **3** (70 mg, 40% yield). The purity of compound **3** was 95.1% as shown by HPLC on a silica column (Dikma, 10 × 250 mm, 10 μm particle size), elution: EtOAc/hexane = 9:1 at a flow rate of 3.0 mL/min, *t*_R_ = 15.9 min. C_36_H_39_N_5_O_8_S; yellow solid; mp 87.5–89.0 °C; TLC (EtOAc/CH_2_Cl_2_ = 1:1)* R*_*f*_ = 0.38; IR ν_max_ (neat) 3358, 3197, 2919, 2849, 1661, 1645, 1622, 1471, 1407 cm^–1^; ^1^H NMR (400 MHz, CDCl_3_) δ 8.55 (t, *J* = 13.5 Hz, 1 H), 7.86 (d, *J* = 8.6 Hz, 2 H), 7.81 (d, *J* = 8.9 Hz, 1 H), 7.39 (t, *J* = 7.8 Hz, 1 H), 7.27 (s, 1 H), 7.05–6.96 (m, 2 H), 6.82 (d, *J* = 8.5 Hz, 1 H), 6.68 (d, *J* = 8.6 Hz, 2 H), 6.42 (t, *J* = 5.1 Hz, 1 H), 4.86 (q, *J* = 4.3 Hz, 1 H), 4.13 (t, *J* = 3.9 Hz, 2 H), 3.84 (t, *J* = 4.6, 2 H), 3.72–3.67 (m, 2 H), 3.67–3.59 (m, 8 H), 3.38 (q, *J* = 5.2 Hz, 2 H), 2.99 (s, 6 H), 2.80–2.60 (m, 3 H), 2.04 (t, *J* = 6.3 Hz,1 H); ^13^C NMR (100 MHz, CDCl_3_) δ 171.2, 169.2, 168.5, 167.5, 166.5, 156.2, 151.9, 148.6, 146.7, 135.9, 135.4, 132.4, 128.5 (2 ×), 122.5, 121.2, 116.7, 115.4, 111.7 (2 ×), 111.5, 110.1, 105.4, 70.7, 70.6 (3 ×), 69.6, 69.4, 68.1, 48.8, 42.3, 40.1 (2 ×), 31.3, 22.6. ESI-HRMS calcd for C_36_H_40_N_5_O_8_S: 702.2592, found: *m/z* 702.2589 [M + H]^+^.

#### 4-((14-((2-(4-(Dimethylamino)phenyl)benzo[*d*]thiazol-6-yl)oxy)-3,6,9,12-tetraoxatetradecyl)amino)-2-(2,6-dioxopiperidin-3-yl)isoindoline-1,3-dione (4)

By a procedure similar to that for compound **1**, the substitution reaction of **10** (102 mg, 0.37 mmol) with **9d** (90 mg, 0.18 mmol) gave a crude product, which was purified by flash chromatography on a silica gel column with elution of EtOAc/CH_2_Cl_2_(2:1) to give the desired compound **4** (89 mg, 65% yield). The purity of compound **4** was 99.3% as shown by HPLC on a silica column (Dikma, 10 × 250 mm, 10 μm particle size), elution: EtOAc/MeOH = 99:1 at a flow rate of 3.0 mL/min, *t*_R_ = 11.3 min. C_38_H_43_N_5_O_9_S; yellow solid; mp 77.5–79.0 °C; TLC (EtOAc/ CH_2_Cl_2_ = 2:1)* R*_*f*_ = 0.38; IR ν_max_ (neat) 3356, 3197, 2921, 2851, 1653, 1634, 1470, 1456, 1368, 742, 701 cm^–1^; ^1^H NMR (400 MHz, CDCl_3_) δ 8.66 (s, 1 H), 7.85 (d, *J* = 8.8 Hz, 2 H), 7.80 (d, *J* = 8.9 Hz, 1 H), 7.40 (t, *J* = 7.8 Hz, 1 H), 7.27 (d, *J* = 2.4 Hz, 1 H), 7.05–6.96 (m, 2 H), 6.83 (d, *J* = 8.6 Hz, 1 H), 6.68 (d, *J* = 8.8 Hz, 2 H), 6.42 (t, *J* = 5.4 Hz, 1 H), 4.85 (q, *J* = 5.2 Hz, 1 H), 4.14 (t, *J* = 4.7 Hz, 2 H), 3.84 (t, *J* = 4.7, 2 H), 3.73–3.67 (m, 2 H), 3.67–3.57 (m, 12 H), 3.38 (q, *J* = 5.4 Hz, 2 H), 2.99 (s, 6 H), 2.85–2.60 (m, 3 H), 2.07–1.97 (m, 1 H); ^13^C NMR (100 MHz, CDCl_3_) δ 171.3, 169.1, 168.5, 167.5, 166.5, 156.1, 151.8, 148.7, 146.7, 135.9, 135.5, 132.4, 128.4 (2 ×), 122.5, 121.3, 116.7, 115.4, 111.7 (2 ×), 111.5, 110.1, 105.3, 70.8, 70.6, 70.5 (2 ×), 70.4 (2 ×), 69.6, 69.3, 68.0, 48.7, 42.2, 40.1 (2 ×), 31.3, 22.6. ESI-HRMS calcd for C_38_H_44_N_5_O_9_S: 746.2854, found: *m/z* 746.2888 [M + H]^+^.

### Cereblon binding assay

A cereblon TR-FRET binding assay was developed by using XL-665-labelled thalidomide (Perkin Elmer) and a specific glutathione *S*-transferase (GST) antibody labelled with europium cryptate which binds GST-tagged human cereblon/DDB1 protein. This assay can be used to detect competitive ligand that replaces the binding of thalidomide to human cereblon/DDB1 protein. GST-tagged human cereblon and DDB1 protein was co-expressed by using baculovirus expression system. The GST-tagged protein complex was purified by using Glutathione Sepharose (Cytiva LifeSciences) and the purity of the recombinant protein was confirmed by SDS-PAGE. The competitive binding of a given compound was measured by incubating various concentrations of compounds with 100 nM cereblon/DDB1 in a buffer containing 50 mM Tris at pH 7.5 and 200 mM NaCl. Final concentration of DMSO was kept at 2.5%. Subsequently, the reaction was added 20 μL of 10 nM XL-665-labelled-thalidomide and 100 nM europium cryptate-labelled GST antibody. All assays were performed in 384-well plates (Geiner Bio-One) and the signals were measured using a Pherastar (BMG) plate reader with excitation at 337 nm and emission at 665 nm/620 nm for detection. The ratio of the acceptor (XL665, 665 nm) and the donor (europium-cryptate, 620 nm) emission signals were used for calculation of EC_50_ values by using a nonlinear fit model (GraphPad Prism Software). Data was presented as means ± standard deviation (n = 3).

### Plasmid constructs

The cDNA encoding N-terminal truncated TDP-43 (TDP-43_208–414_, C-TDP-43) were constructed into pEGFP-C3 vector (kindly provided by Pang-Hsien Tu’s lab in the Institute of Biomedical Sciences (IBMS), Academia Sinica). To get the mCherry-C-TDP-43 and FLAG-C-TDP-43 plasmid, C-TDP-43 was subcloned from pEGFP-TDP-43_208–414_ construct into pcDNA3.1-mCherry and pCMV-tag2B vector, respectively. Either eGFP, mCherry, and FLAG was fused N-terminally of TDP-43_208–414_. The pEGFP-C3 and pcDNA3.1-mCherry plasmid were used as control plasmids for FLIM-FRET experiments.

### Cell culture

Neuro-2a cell is the mouse neuroblastoma cell line from Dr. Yijuang Chern (IBMS, Academia Sinica). Neuro-2a cells were cultured in Dulbecco’s modified Eagle’s medium (DMEM; Gibco) containing 2 × 10^−3^ M glutamine, 10% heat inactivated fetal bovine serum, and 100U mL^−1^ penicillin–streptomycin (Gibco) at 37 °C in a humidified incubator containing 5% CO_2_.

### TDP-43 Fractionation on Neuro-2a cells

This method demonstrated how to collect the RIPA-insoluble C-TDP-43 aggregates for SDS-PAGE analysis. RIPA buffer-lysed C-TDP-43 expressing Neuro-2a cells were centrifuged at 70,000*g* at 4 °C for 40 min (Optima™ MAX-XP Ultracentrifuge, Beckman Coulter). The supernatant (RIPA-soluble fraction) was carefully collected and the pellet of C-TDP-43 aggregates was further washed with RIPA buffer. During the washing step, the pellet was resuspended by 200 μL RIPA buffer and centrifuged at 70,000 g at 4 °C for 10 min. After centrifugation, the supernatant was carefully removed. Then, the washing step was repeated to remove most of the remaining RIPA-soluble protein. Lastly, 50 μL 1% sarkosyl buffer (1 g sarkosyl in 50 mL PBS buffer) was added into the pellet tube, followed by intense pipetting to resuspend the pellet (sarkosyl-soluble fraction). Both the RIPA-soluble and insoluble samples were further processed for detection by using western blot.

### AlamarBlue reduction assay

Neuro-2a cells were seeded in a 24-well plate at a concentration of 8 × 10^4^ cells/well and incubated overnight. The attached Neuro-2a cells were then transfected with the eGFP-C-TDP-43 plasmid (1.1 μg) using TurboFect™ transfection reagent (Invitrogen) according to manufacturer’s recommendations. After 2 h transfection, Neuro-2a cells were further treated with or without 5 μM PROTAC (compounds **1**–**4**). The cell viability indicator, AlamarBlue (Invitrogen), was added after 42 h of incubation, and the mixture was incubated for another 6 h (total duration time = 48 h). Two-hundred μL conditioned medium was transferred to a 96-well plate, and cell viability was determined by the increased fluorescence intensity (λ_ex_ = 560 nm, λ_em_ = 590 nm).

### Filter trap assay and slot blot assay

Neuro-2a cells were seeded in a 6-well plate at a concentration of 2 × 10^5^ cells/well and incubated overnight. Then, Neuro-2a cells were transfected with the eGFP-C-TDP-43 plasmid (2.2 μg) using Turbofect transfection reagent (Invitrogen) and treated with or without 5 μM PROTAC molecules after 2 h transfection. After incubation for another 22 h, the transfected cells were harvested by RIPA buffer containing protease inhibitor (Roche) and sonicated on ice for 10 s. Extracts were centrifuged at 14,000 rpm for 10 min at 4 °C, followed by measurement of protein concentration using a bicinchoninic acid (BCA) assay. For filter trap assay, 300 μL sample (100 μg total protein) were passed through 0.2 μm cellulose acetate (CA) membranes (OE66, GE Healthcare) membranes using a 48-well slot-blot apparatus. Aggregated eGFP-C-TDP-43 protein retained on CA membranes was determined by immunoblotting with TDP-43 (C-terminal) antibody (1:1000, Proteintech, 12,892–1-AP). Amersham™ protein® Nitrocellulose (NC) membranes (pore size 0.1 μm, GE Healthcare) were applied to slot blot assay for soluble protein lysate analysis (endogenous TDP-43 and loading control).

### In vitro protein binding assay

Neuro-2a cells were seeded in 10 cm dishes at a concentration of 1 × 10^6^ (blank control) or 2 × 10^6^ (mCherry-C-TDP-43 overexpression) cells/mL. After overnight incubation, Neuro-2a cells were transfected with the mCherry-C-TDP-43 plasmid (10 μg) using Lipofectamine® 3000 (Invitrogen) and incubated for another 24 h. The transfected cells were then harvested with RIPA buffer containing complete protease inhibitor (Roche) and sonicated on ice (10 s, two repetitions). Next, PROTAC **2** at various concentrations (0, 5, 10, 20, and 40 μM) was added to each extract (identical protein quantity, 100 μg). The mixtures (PROTAC **2 + **cell lysate**)** were gently shaken at 4 °C for 2 h. After that, the mixtures were fractionated and the RIPA-insoluble fraction were resuspended with RIPA buffer and loaded on CA membrane by applying filter trap assay. The fluorescent PROTAC **2** retained on C-TDP-43 aggregates was detected by Typhoon9410 Variable Mode Imager (Amersham BioScience, Piscataway, NJ, USA) (λ_ex_ = 457 nm, λ_em_ = 488 nm).

### Epifluorescence microscopy

Neuro-2a cells [2 × 10^6^ cells in sterile 35 mm µ-Dish (ibidi, Martinsried, Germany)] were transfected with mCherry-C-TDP-43 (1.1 µg) by TurboFect transfection reagent (Invitrogen). For quantifying the C-TDP-43 aggregates in the presence or absence of PROTAC **2** or MG132, mCherry-C-TDP-43 transfected cells were treated with PROTAC **2** (5 µM) after 2 h of transfection. For the group intended to block proteasome activity, MG132 (2 μM) was pre-treated 1 h before adding PROTAC **2**. After total 24 h incubation, the Neuro-2a cells were fixed (4% paraformaldehyde in 15 min and stored in 1 × PBS buffer) and subsequently imaged with NIKON TiE microscope. Epifluorescence images were illuminated with an ultrahigh pressure mercury lamp (130 W) for UV excitation or using a 488 nm laser light source. Filters were used to collect fluorescence emission including excited eGFP (excitation D480/40, dichroic D505LP, emission D535/50) and mCherry (excitation D535/50, dichroic D565LP, emission D590LP) cubes. Cellular images were captured with an Andor iXon3 888 back-illuminated high-sensitivity EMCCD camera. Images were edited and cropped using Nikon NIS element software.

### Confocal microscopy

To ensure the colocalization event of PROTAC **2** and C-TDP-43 aggregates as well as the expression yield of either co-expressed eGFP and mCherry or eGFP-C-TDP-43 and mCherry-C-TDP-43, 8 × 10^5^ of Neuro-2a cells were seeded in 6-well plate with a 30 mm square coverslip. After O/N incubation, cells were transfected with either mCherry-C-TDP-43 (1.1 μg), eGFP/mCherry (0.55 μg each), or eGFP-C-TDP-43 /mCherry-C-TDP-43 (0.55 μg each) plasmids with Turbofect transfection reagent (Invitrogen) according to the manufacturer’s protocol. After treatment with PROTAC **2**, cells were incubated for another 22 h and then fixed with 4% paraformaldehyde in 15 min and stored in 1 × PBS buffer. Confocal images were captured with confocal laser scanning microscopy (FV3000, Olympus, Japan). The 405 nm laser was used for excitation of PROTAC **2** with emission 415–470 nm bandpass filter. The 488 nm laser was used for excitation of eGFP with emission 507–540 nm bandpass filter. The 561 nm laser was used for excitation of mCherry with emission 610–640 nm bandpass filter.

### Frequency-domain fluorescence lifetime imaging

To study the E_FRET_ of oligomeric intermediates of C-TDP-43, we seeded 2 × 10^5^ cells/dish of Neuro-2a cells in sterile 35 mm µ-Dish and transfected with either both 0.55 µg eGFP-C-TDP-43 and 0.55 µg mCherry-C-TDP-43 or both 0.55 µg eGFP and 0.55 µg mCherry (negative control) plasmids. After 2 h, PROTAC **2** was delivered to the experimental group. After 48 h incubation, the Neuro-2a cells were fixed (4% paraformaldehyde in 15 min and stored in 1 × PBS buffer) and further analyzed by Q2 FastFLIM system (ISS Inc.). The Neuro-2a cells were monitored and captured under oil-immersion objective observation [ Nikon Plan Apo 100 × /numerical aperture (NA) 1.4]. The eGFP-C-TDP-43 excitation sources came from 488 nm (5 mW) sub-nanosecond modulated pulsed laser at the fundamental frequency of 20 MHz was controlled by ISS VistaVision software. The photon counts of eGFP were collected by GaAs photomultiplier tube (PMT) detector with EM1 filter (530/43 nm bandpass filter). To precisely obtain the lifetime value, the calibration of the system was operated by measuring fluorescein, a fluorophore with a single exponential lifetime around 4 ns in ddH_2_O, every time before the measurement.

### FLIM-FRET data analysis

The fitting method of the FastFLIM images were detailed in the “Experimental” section of a previous publication [52]. For the “frame” lifetime fitting model, the lifetime of each pixel in FLIM images (Additional file 1: Fig. S8A) were directly obtained by ISS Software VistaVision and subsequently transformed into E_FRET_ maps and per-pixel distribution histogram (the population of each pixel corresponding to the E_FRET_ maps) (Fig. 4B).

For the “highlighted-pixel” lifetime fitting model, first, we ensured that PROTAC **2** would not form crosstalk with the eGFP donor (Additional file 1: Fig. S7). Then, by thresholding photon counts of the eGFP-C-TDP-43 against reddish pixels (high photon counts) in FLIM image (Additional file 1: Fig. S8A), we were able to filter out the aggregate species and leave the soluble C-TDP-43 (namely monomer and oligomer, shown in purple masking) corresponding to the phasor plot (Additional file 1: Fig. S8B). After that, the highlighted soluble regions were fitted with 2-exponential fitting (Additional file 1: Fig. S8C) to get the average lifetime (Fig. 4C) as well as the fraction (Fig. 4D) of C-TDP-43 oligomeric intermediates. To obtain the lifetime of C-TDP-43 oligomeric intermediates under two-exponential fitting, the lifetime of eGFP-C-TDP-43 monomers were fixed as 2.6 ns (the lifetime of eGFP). The FRET efficiency of oligomeric intermediates (Fig. 4C) was calculated by the formula: ${E}_{FRET}=\frac{{\tau }_{D}-{\tau }_{DA}}{{\tau }_{D}}=1-\frac{{\tau }_{DA}}{{\tau }_{D}}$, wherein ${\tau }_{D}$ is donor lifetime of eGFP only, and ${\tau }_{DA}$ denotes lifetime of intermediates.

To fairly judge the E_FRET_ oligomeric intermediates, the Neuro-2a expressing 2FP-C-TDP-43 were arbitrarily selected. All of the lifetime values in this study were carefully fitted in a reasonable range with the acceptable chi-square value (*χ*^2^).

### Size exclusion chromatography (SEC)

Neuro-2a cells harboring eGFP-C-TDP-43 (10 µg) were seeded in 10 cm dishes (2 × 10^6^ cells/dish) and treated after 2 h with PROTAC **2** (5 µM) or both PROTAC **2** (5 µM) and MG132 (2 µM). After 48 h incubation, the transfected cells were harvested in 1000 μL of ice-cold RIPA buffer containing protease inhibitor cocktail (Roche) and sonicated on ice for 1 min. Extracts were centrifuged at 14,000 rpm for 10 min at 4 °C and the protein concentrations were determined using BCA assay. Samples containing 300 μg of total proteins in a volume of 500 μL were filtered with a 0.22 μm filter (Millipore) and fractionated on a Superdex 200 10/300 column (GE Healthcare) at a flow rate of 0.3 mL/min. Each fraction (1 mL volume/fraction) was collected and subjected to western blot and slot blot analysis.

### Western blot

For gel electrophoresis and blotting, all the necessary materials and procedures were described in a previous paper [53]. Proteins were separated using 12% Tris–glycine SDS-PAGE. Proteins were transferred onto PVDF membrane (Millipore). Blots were blocked with 5% bovine serum albumin (BSA, Sigma) in 0.1% PBST for at least 1 h. After blocking, blots were subjected to incubation with the primary antibodies TDP-43 (C-terminal) (1:1000, Proteintech, 12,892-1-AP), TDP-43 (1:1000, Abcam, ab104223), p-TDP-43 (pS409/410) (1:1000, Cosmo Bio, TIP-PTD-M01), GFP (1:1000, Abcam, ab183734), A11 (1:1000, Invitrogen, AHB0052), GAPDH (1:10,000, GeneTex, GTX627408), GSPT1 (1:1000, Proteintech, 10,763-1-AP), flag M2 (1:1000, Sigma, F1804), LC3B (1:1000, cell signaling, #2775), γH2A.X (phosphor-Ser139) (1:1000, Merck Millipore, 05–636), HSP70 (1:1000, Proteintech, 10,995-1-AP), HMGB1 (1:1000, Abcam, ab18256) or β-actin (1:10,000, GeneTex, GTX109639) in 2–5% BSA and incubated overnight at 4 °C on a shaker. After washing with 0.1% PBST, the blots were further incubated with HRP-labelled secondary antibodies [1:15,000, anti-Rabbit (GeneTex, GTX213110-01), anti-Mouse (Jackson ImmunoResearch Laboratories, Inc., 115-035-003)] at room temperature for another 2 h. The blots were washed and developed with electrochemiluminescence (ECL, Millipore). The signals were visualized with luminescence (iBright™ FL1000 instrument, Invitrogen).

### *C. elegans* strains maintenance and behavioral assays

The YFP-C-TDP-43 and YFP transgenic strains of *C. elegans* generated in this study were IW33 [*Psnb-l*::C-TDP-43_219–414_-YFP (*iwIs22*)] and IW62 [*Psnb-1*::YFP(*iwIs25*)], respectively (kindly provided by Prof. Jiou Wang at Bloomberg School of Public Health, The Johns Hopkins University). The strains of nematodes were maintained with standard procedure and grown at 20 °C [54]. For larvae synchronization, the eggs were isolated by lysing gravid adult worms with freshly prepared bleaching solution (0.5 mL 5 M NaOH with 1 mL bleach) and incubated in S buffer (129 mL 0.05 M K_2_HPO_4_, 871 mL 0.05 M KH_2_PO_4_, 5.85 g NaCl) for overnight. For drug treatments, PROTAC **2** or/and DMSO were solely or along with MG132 applied to fresh NGM plates prior to installing the synchronized *C. elegans*. The body bends of the corresponding treatment in a duration of 30 s of the various strains were documented through SMZ800N stereomicroscope equipped with a CCD camera (Nikon). A body bend was counted as the head of one-day adult *C. elegans* travels across the mid-body in 1 × PBS buffer. Then, the bending videos of *C. elegans* were analyzed by ImageJ with the wrMTrck plugin [55]. For monitoring the effects of PROTAC **2** on C-TDP-43 accumulation in L4 worms, the confocal images were captured with LSM 780 (Zeiss).

### Statistical analysis

Statistical comparison of multiple independent groups was conducted by one-way ANOVA with Tukey or Dunnett post-hoc test. Two-way ANOVA with False Discovery Rate post-hoc test was used to determine the effect of two nominal predictor variables on a continuous outcome variable. Statistical comparison of two independent groups was done by two-tailed unpaired t-tests. Significance was accepted at p < 0.05. ns is not significant. All the statistical figures and analysis were done by GraphPad Prism9 software.

## Results

### Design and synthesis of PROTAC molecules

Our investigation began with designing TDP-43 PROTACs. To date, no small molecule as C-TDP-43 ligand is discovered. To find potential ligand, we surveyed literature to study the main structure of C-TDP-43 rather than individual binding pocket. Several peptidyl fragments of C-TDP-43 protein exist in β-sheet structures when forming aggregates or fibrils [38, 56–60], which can bind to amyloid dyes including thioflavin T (ThT) [61–64]. After removal of the methyl group from the quaternary nitrogen atom in the benzothiazole moiety of ThT, the electrically neutral benzothiazole-aniline (BTA) molecule is predicted to have higher binding affinity to β-sheet structures [61] and better cell permeability [49]. Furthermore, it is known that installation of a substituent at the 6-position of the BTA core would not interfere with its binding with amyloid [61]. Therefore, we decided to utilize the BTA compound **7** (Fig. 1B) as a suitable binder to C-TDP-43 aggregates. To activate ubiquitin proteasome system (UPS)-mediated degradation, pomalidomide (POM) and lenalidomide have been widely used to recruit CRBN complex for the protein degradation [26–28, 65]. We chose polyethylene glycol (PEG) to construct the linkers because PEGs were readily accessible by synthesis and allow fine-tuning of the putative linker length [66]. In fact, both the linker length and composition play important roles on the physicochemical properties and bioactivity of PROTACs [66]. Meanwhile, hydrophilic PEG linker can ameliorate the hydrophobicity penalty caused by the BTA moiety, and therefore helps balance the lipophilicity and solubility of PROTACs.

Bearing the linker optimization issue in mind, we synthesized PROTACs **1**–**4** with the linkers containing 2–5 units of ethylene glycol, respectively (Fig. 1B). The bromo compound **5** and 4-(dimethylamino)phenylboronic acid (**6**) underwent Suzuki coupling reaction by the catalysis of Pd(dppf)Cl_2_ to form anisole **7** as the core structure of TDP-43 binder [49]. Anisole **7** was treated with BBr_3_ at 0 °C to obtain the 6-hydroxy substituted BTA (**8**). As an example, BTA **8** was subjected to alkylation with a PEG linker N_3_CH_2_CH_2_(OCH_2_CH_2_)OMs in the presence of K_2_CO_3_, followed by reduction of the azido group to amino group, to afford compound **9a**. The conjugation of **9a** with a pomalidomide analog **10**, which bears a fluorine atom at the 4-position of the isoindoline ring, was conducted in 1-methyl-2-pyrrolidone (NMP) [51], an aprotic solvent with high polarity, to give the desired PROTAC **1** (JMF4605). Other PROTAC molecules **2**–**4** (JMF4560, JMF4590 and JMF4583) having different PEG units were similarly synthesized (Supplementary Scheme S1 and S2). The successful conjugation reaction should not be performed under alkaline conditions in order to retain the imide groups in the pomalidomide structure.

### PROTAC 2 facilitates degradation of C-TDP-43 aggregates and enhances cell viability

**Fig. 2:**
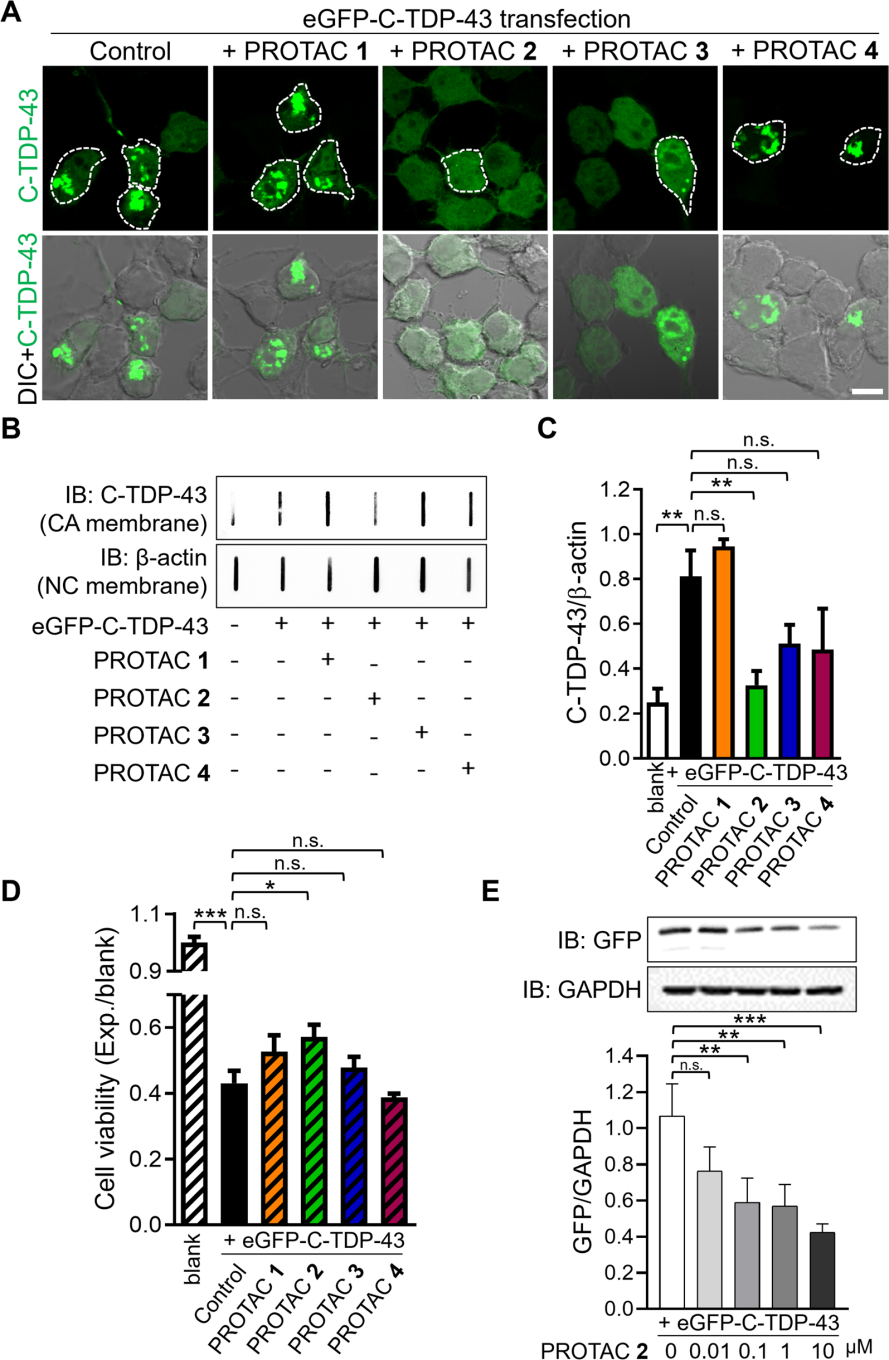
Examination of the C-TDP-43 disaggregation and beneficial effects of PROTACs **1–4**. **A** Representative images of eGFP-C-TDP-43-expressing Neuro-2a cells with or without PROTAC **1**–**4** (5 μM). Scale bar = 10 μm. **B** Filter trap assay of eGFP-C-TDP-43 expressed Neuro-2a cells in the presence and absence of PROTACs **1**–**4** (5 μM). The cell lysate was either loaded on cellulose acetate (CA) or nitrocellulose (NC) membrane probed with TDP-43 (C-terminal) antibody and β-actin antibody (loading control), respectively. **C** Quantification of blots in panel B. **D** AlamarBlue reduction assay of eGFP-C-TDP-43 expressed Neuro-2a cells treated with PROTACs **1**–**4** (5 μM). **E** Western blot of eGFP-C-TDP-43 transfected Neuro-2a cells treated with various concentrations of PROTAC **2**. The RIPA-insoluble fraction and RIPA-soluble fraction of Neuro-2a lysate were further probed with GFP and GAPDH antibody, respectively. All the statistic results were quantified by ImageJ and shown as mean ± SD (n ≥ 3). Data were analyzed by one-way ANOVA with Dunnett post-hoc test (*P < 0.05, **P < 0.01, ***P < 0.001)

Currently, PROTACs have been exploited to target on total tau [29], mutant tau [30], mutant huntingtin [7, 15], and amyloid-beta aggregates [31]. Although these endeavors have made huge progress on disease-related protein degradation, treatment of TDP-43 proteinopathy still remains unsolved. Along this line, we examined whether PROTACs **1–4** can induce C-TDP-43 aggregates degradation by expressing eGFP-TDP-43_208–414_ (hereafter referred as eGFP-C-TDP-43) in Neuro-2a cells (Fig. 2A). Among these candidates, PROTAC **2** treated group substantially exhibited less cytoplasmic C-TDP-43 aggregates compared to either control or other PROTACs. To quantify the remaining amount of C-TDP-43 aggregates upon PROTACs treatment, we performed filter trap assay and immunoblotting by applying the Neuro-2a lysate on cellulose acetate (CA) membrane (Fig. 2B). The C-TDP-43 amount on CA membrane (normalized with the corresponding loading control) indicated that overexpressing eGFP-C-TDP-43 in Neuro-2a cells did induce a significant amount of C-TDP-43 aggregates (control, 1.08 ± 0.31, the black bar in Fig. 2C) compared with that in blank (0.38 ± 0.08, the white bar in Fig. 2C). Interestingly, treatment with PROTAC **2** (JMF4560) markedly reduced the eGFP-C-TDP-43 aggregates (0.41 ± 0.06, the green bar in Fig. 2C) than other PROTAC-treated groups (0.64–1.31, the orange, blue, and the deep pink bars in Fig. 2C).

In addition to the degradation ability, we also evaluated the possible cytotoxicity of PROTACs **1–4** to Neuro-2a cells before evaluating their therapeutic potential. According to the slot blot result, PROTAC **2** (1.11 ± 0.14, the green bar in Figure S1A) and other PROTAC-treated groups did not significantly affect endogenous TDP-43 protein level compared to mock (0.91 ± 0.07, the white bar in Figure S1A). PROTACs with shorter linker (PROTAC **1** and** 2**) showed neglectable cytotoxicity compared to mock group of Neuro-2a cells (Additional file 1: Fig. S1B). Given facts that cellular aggregates are considered as a cytotoxicity agent [67], we further examined whether PROTACs **1**–**4** could relieve C-TDP-43-mediated cytotoxicity. Compared to blank (1.0 ± 0.03, the white bar in Fig. 2D), overexpression of eGFP-C-TDP-43 caused a decrease in cell viability (0.43 ± 0.04, the black bar in Fig. 2D). Notably, PROTAC **2** significantly enhanced cell viability (0.56 ± 0.04, green bar in Fig. 2D) as compared to the control group and other PROTAC-treated groups (0.37–0.50, other bars in Fig. 2D). In line with the cell viability results, we found that PROTAC **2** was capable of reducing the level of apoptosis-associated protein (γH2A.X) (Additional file 1: Fig. S1C, D) in response to the reduction of C-TDP-43 aggregates (Additional file 1: Fig. S1C and E). By contrast, PROTAC **2** showed neglectable effect on regulating cell necrosis (HMGB1, Additional file 1: Fig. S1F, G), autophagy (LC3B, Additional file 1: Fig. S1F, H), and protein folding (HSP70, Additional file 1: Fig. S1F, I).

As PROTAC **2** outperforms other PROTAC candidates in respect to eGFP-TDP-43 removal and antagonizing TDP-43-mediated cytotoxicity, it was further selected for examining the specificity in degradation of C-TDP-43 aggregates. Our data showed that PROTAC **2** exhibited significant degradation of eGFP-C-TDP-43 aggregates in a dosage-dependent manner (Fig. 2E). Meanwhile, PROTAC **2** displayed similar degradation capability toward FLAG-tagged C-TDP-43 aggregates (Additional file 1: Fig. S2A) but not eGFP (Additional file 1: Fig. S2B), supporting the distinct impact of PROTAC **2** on C-TDP-43 degradation. Since it has been reported that POM-based PROTAC may reduce the ectopically-expressed protein caused by mishit-degradation of translation regulator protein (GSPT1) [51], we also monitored the GSPT1 level upon PROTAC **2** treatment. According to the western blot results, GSPT1 level remains stable irrespective of PROTAC **2** treatment at various concentrations (Additional file 1: Fig. S2C), ensuring the PROTAC **2** degradation efficacy is specific to C-TDP-43 aggregates. Taken together, our data suggested that PROTAC **2**-mediated C-TDP-43 aggregates degradation was capable of alleviating C-TDP-43-induced apoptosis and eventually decreased C-TDP-43 cytotoxicity.

### PROTAC 2 binds to E3 ligase (CRBN) and neo-substrate (C-TDP-43 aggregates)

To learn the specificity of PROTAC drugs to the engaged E3 ligase, we determined the binding of PROTACs **1–4** to CRBN with an in vitro time-resolved fluorescence resonance energy transfer (TR-FRET) assay (Additional file 1: Fig. S3A) [49, 51]. By measuring the competitive binding of PROTACs **1–4** versus thalidomide to E3 ligase, all PROTACs **1–4** showed comparable binding affinity to the E3 ligase (recombinant CRBN protein was used here) with the EC_50_ values in the range of 0.6–2.6 μM (Additional file 1: Fig. S3B), which was similar to that of the sole pomalidomide (1.9 μM), indicating the robust binding of PROTAC drugs to its E3 ligase. Among PROTACs **1–4**, PROTACs **2** exhibited highest binding affinity (0.6 μM). We also examined whether PROTAC **2** can bind to C-TDP-43 aggregates by in vitro protein binding assay (Additional file 1: Fig. S4A, Details in “Materials and methods” section). According to the Additional file 1: Fig. S4B, PROTAC **2** significantly bound to the mCherry-C-TDP-43 aggregates retained on the filter membrane compared to control. However, based on the aggregation prone property of C-TDP-43, determining its precise K_d_ value for PROTAC **2** is difficult. To further confirm the interaction between C-TDP-43 aggregates and PROTAC **2** at lower concentration, we applied fluorescence microscopy to check the colocalization event between PROTAC **2** and C-TDP-43 aggregates in the mCherry-C-TDP-43 harboring Neuro-2a cells. As shown in Additional file 1: Fig. S4C, the fluorescent PROTAC **2** (5 µM) colocalized with mCherry-C-TDP-43 aggregates (Additional file 1: Fig. S4C and E) but not the untreated group (Additional file 1: Fig. S4C, D), suggesting the interaction between PROTAC **2** and its neo-substrate in cells. Collectively, we confirmed that PROTAC **2** can form the binary complex with either CRBN or C-TDP-43 aggregates.

### PROTAC 2 degrades C-TDP-43 aggregates via ubiquitin–proteasome system

Both ubiquitin–proteasome system (UPS) and autophagy pathway may account for the clearance of misfolded TDP-43 [56]. Generally, POM-based PROTACs recruit CRBN complex and neo-substrates to guide neo-substrates to proteasome for degradation [68]. To verify whether UPS pathway dominated in the PROTAC **2**-mediated degradation of C-TDP-43 aggregates, we made use of a proteasome inhibitor, MG132, to the eGFP-C-TDP-43-expressing Neuro-2a cells in the presence of PROTAC **2**. Due to the poor solubility eGFP-C-TDP-43 aggregates, it was collected in the RIPA-insoluble fraction from the cell lysate by ultracentrifugation (Details in “Materials and methods” section). Both RIPA-soluble and insoluble fractions were further analyzed by SDS-PAGE with immunoblotting.

**Fig. 3:**
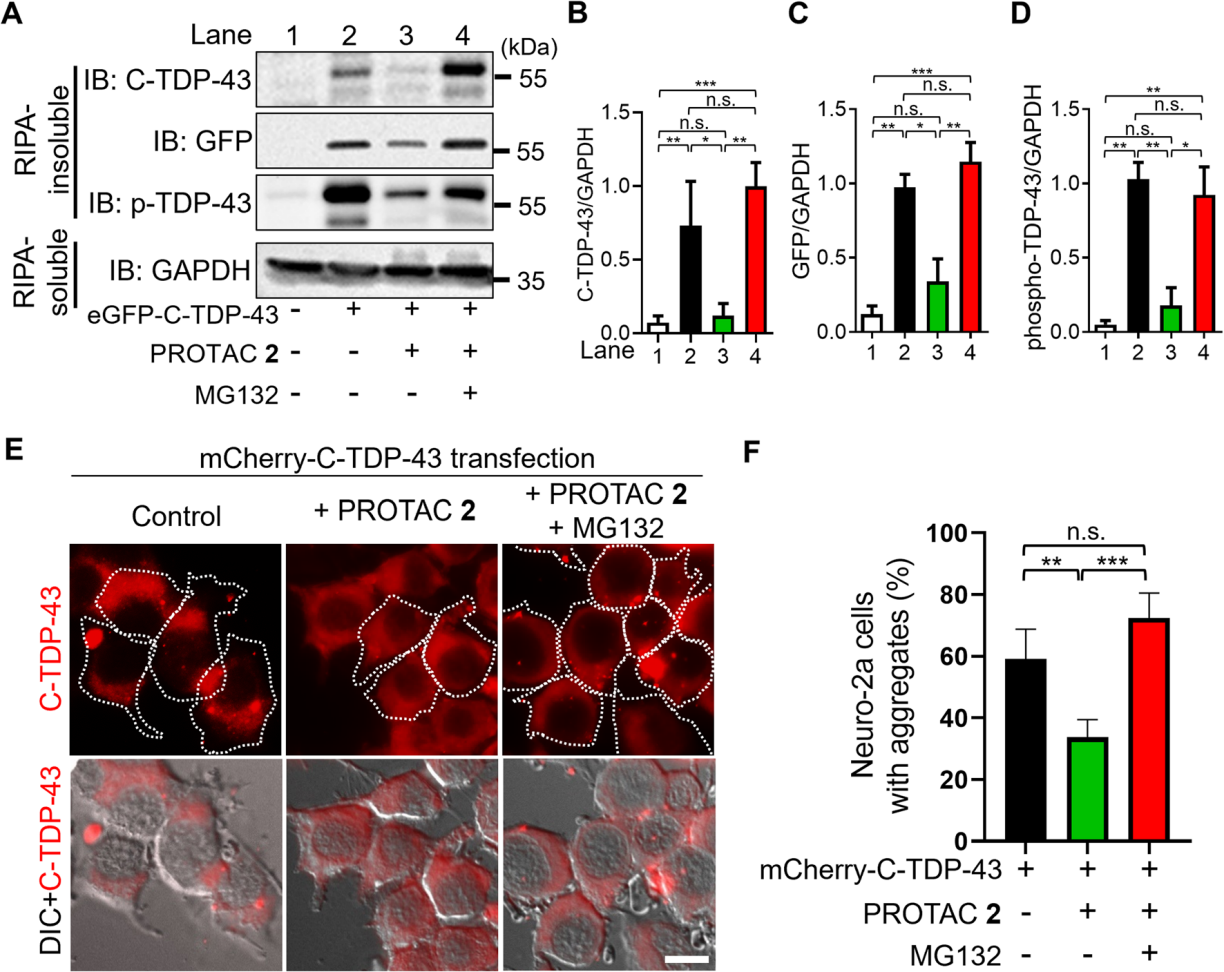
PROTAC **2** decreased insoluble C-TDP-43 aggregates via UPS. **A** Western blot of eGFP-C-TDP-43 harboring Neuro-2a cells with or without PROTAC **2** (5 μM) or/and MG132 (2 μM) treatment. The blots of RIPA-insoluble and RIPA-soluble cell lysates were demonstrated by SDS-PAGE and probed with C-TDP-43, GFP, phospho-TDP-43 (Ser409/410), and GAPDH antibodies. **B**–**D** Quantification of blots of C-TDP-43 (**B**), GFP (**C**), and phospho-TDP-43 (**D**) in panel A. **E** Representative images of mCherry-C-TDP-43 expressed Neuro-2a cells with or without PROTAC **2** (5 μM) or/and MG132 (2 μM). To morphologically monitor the C-TDP-43 puncta upon drug treatment, the cells with mCherry-C-TDP-43 puncta larger than 0.1 μm^2^ were considered as the aggregate-positive cells (featured with dash line). Scale bar = 10 μm. **F** Quantification of the percentage of aggregate-positive Neuro-2a cells among total cells in panel E. All the statistic results were quantified by ImageJ and shown as mean ± SD (n ≥ 3). Data were analyzed by one-way ANOVA with Tukey post-hoc test (*P < 0.05, **P < 0.01, ***P < 0.001)

Since the endogenous TDP-43 could also precipitated in the presence of C-TDP-43 [69], we employed TDP-43 (C-terminal) antibody to identify endogenous TDP-43 (43 kDa) and GFP antibody for eGFP-C-TDP-43 (53 kDa) in the RIPA-insoluble fraction. According to the representative blot images, RIPA-insoluble eGFP-C-TDP-43 and endogenous TDP-43 aggregates were visualized in the control group (Fig. 3A–C, Lane 2) but not in the mock group (Fig. 3A–C, Lane 1). Unlike PROTAC **2** reduced the amount of insoluble C-TDP-43 (Fig. 3A–C, Lane 3), MG132 reversed the effect of PROTAC **2**. As the insoluble proteins persisted in the presence of MG132 (Fig. 3A–C, Lane 4), the PROTAC **2**-mediated degradation of insoluble C-TDP-43 was dependent of UPS pathway. Since the phosphorylated TDP-43 aggregates were enriched in ALS patients [35], we also employed phospho-TDP-43 antibody in this experiment to assess the phospho-TDP-43 accumulation. The phospho-TDP-43 antibody staining showed the pattern similar to that in TDP-43 (C-terminal) antibody staining, confirming PROTAC **2** could also degrade pathological TDP-43 aggregates (Fig. 3A and D). In contrast, the truncated compounds derived from PROTAC **2** lacking either POM moiety (*i.e.* JMF4576 in Additional file 1: Fig. S5A) or BTA moiety (*i.e.* JMF4565 in Additional file 1: Fig. S5A) failed to degrade insoluble eGFP-C-TDP-43, supporting the essential roles of POM and BTA in bridging the UPS and C-TDP-43 aggregates (Additional file 1: Fig. S5B, C).

In addition to biochemical methods, we also monitored the impact of PROTAC **2** on C-TDP-43 aggregates through epifluorescence microscopy. While mCherry-C-TDP-43 formed aggregates in the cytosol of Neuro-2a cells, the treatment of PROTAC **2** significantly reduced the aggregates (Fig. 3E). Notably, the reduction of aggregates is sensitive to MG132, which was consistent with the western blotting results in Fig. 3A. Statistically, the percentage of cells with cytosolic mCherry-C-TDP-43 puncta (the cells featured with dash line) increased significantly in the double-treated group (PROTAC **2** + MG132) (72%) compared to PROTAC **2** (33%) and the mock (59%) (Fig. 3F). Taken together, we have confirmed that PROTAC **2** targeted and decreased C-TDP-43 aggregates through UPS.

### PROTAC 2 decreases the compactness and population of C-TDP-43 oligomers in cells

**Fig. 4:**
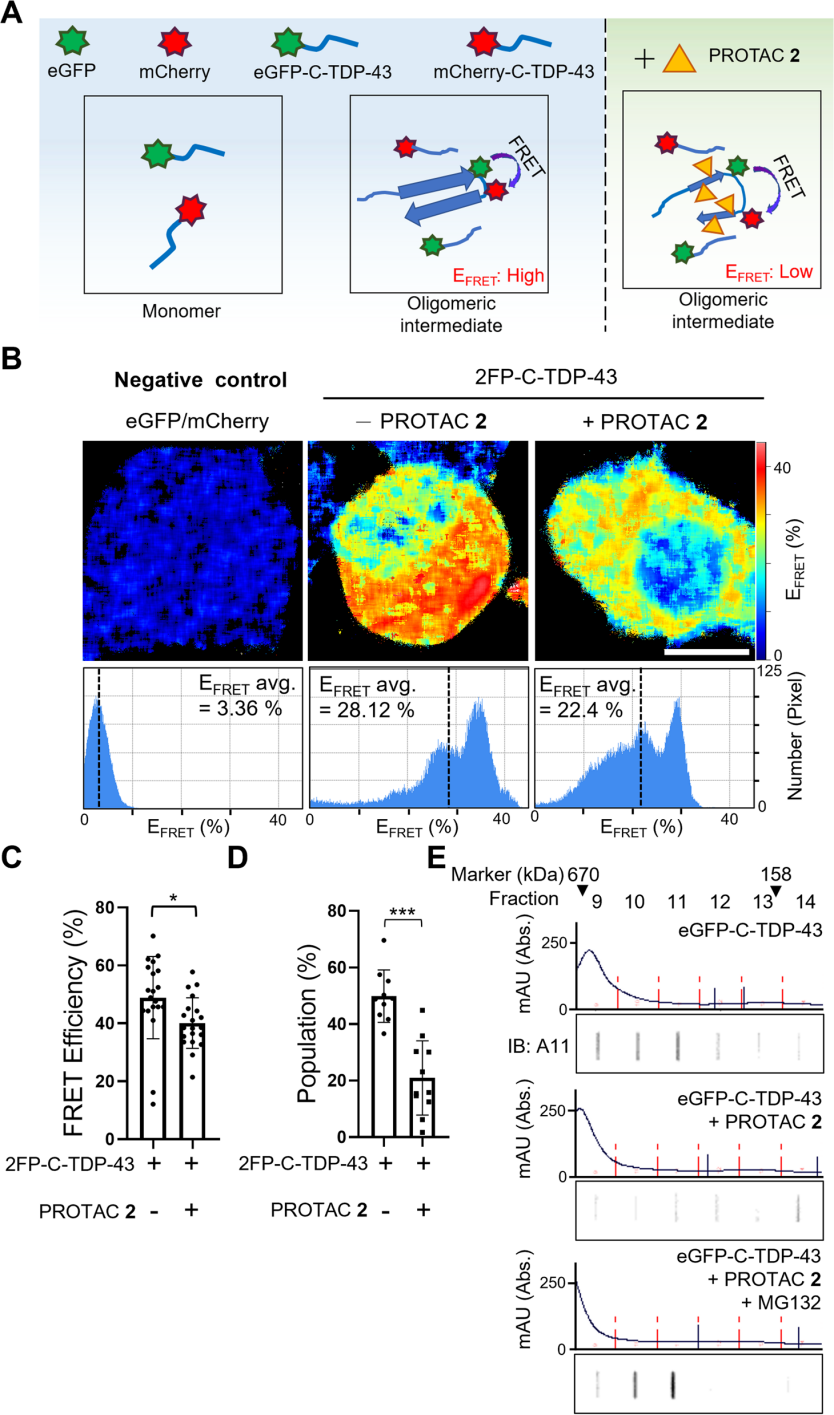
PROTAC **2** decreased the compactness of oligomeric intermediates C-TDP-43 and reduced the high molecular weight oligomers in Neuro-2a cells. **A** Schematic illustration of FLIM-FRET analysis on the 2FP-C-TDP-43 oligomeric intermediates in the cytoplasm of Neuro-2a cells with or without PROTAC **2**. (2FP-C-TDP-43 represents co-expressing eGFP-C-TDP-43 and mCherry-C-TDP-43.) **B** The color-coded images of the E_FRET_ distribution throughout Neuro-2a cells (“frame” fitting model, upper panel) and its per-pixel distribution histograms (lower panel). The palette (color coding on the upper-right) corresponded to the E_FRET_ levels of overall C-TDP-43 species (monomers + oligomeric intermediates + aggregates). **C**, **D** The average E_FRET_ (**C**) and the population (**D**) of C-TDP-43 oligomeric intermediates in 2FP-C-TDP-43 expressed Neuro-2a cells with or without PROTAC **2** (5 μM) by applying “highlighted-pixel” fitting model. Each cell was arbitrarily selected (n = 20) and calculated according to the region average lifetime on a pixel-by-pixel basis. Statistic results were shown as mean ± SD (n ≥ 3). Data were analyzed by two-tailed unpaired t-test (*P < 0.05, ***P < 0.001). **E** Neuro-2a cells expressing eGFP-C-TDP-43 with or without treatment of PROTAC **2** (5 μM) or MG132 (2 μM) were fractionated by applying FPLC on the size exclusion column (SEC). The elution of proteins was monitored by absorbance at 280 nm and fractions were collected every 1 mL. Fractions 9–14 were further loaded on NC membrane and probed with A11 antibody. The elution times of two standards, 670 kDa and 158 kDa, were marked as arrowheads

In addition to TDP-43 aggregates, cumulative studies have also disclosed the pathological role of oligomeric TDP-43 in neurodegenerative diseases [70]. As PROTAC **2** could reduce TDP-43 aggregates, we further explored its possible interference with TDP-43 oligomers. To address this issue, fluorescence lifetime imaging microscopy FRET (FLIM-FRET) was applied to monitor the population of C-TDP-43 oligomer. Since FLIM-FRET reflects the energy transfer through dipole–dipole coupling of a fluorescent donor and acceptor [71], we further created a co-expressing system featuring both eGFP-C-TDP-43 (donor) and mCherry-C-TDP-43 (acceptor) in Neuro-2a cells (Fig. 4A).

Before the lifetime measurement, we first confirmed that eGFP-C-TDP-43 and mCherry-C-TDP-43 (denoted as 2FP-C-TDP-43) had similar expression yield (Additional file 1: Fig. S6). We also showed that the lifetime of eGFP donor stayed consistent in the presence (τ = 2.447 ns, lower panel) or absence (τ = 2.550 ns, upper panel) of fluorescent PROTAC **2** (Additional file 1: Fig. S7). To compare the changes of overall C-TDP-43 species with or without PROTAC **2** treatment, we utilized frequency domain (FD) lifetime fitting analysis (frame model) to obtain the efficiency of FRET (E_FRET_) maps and per-pixel distribution histograms from the FLIM image (Fig. 4B and Additional file 1: Fig. S8A). As a negative control, co-expressing eGFP and mCherry exhibited little or none E_FRET_ (average E_FRET_ = 3.36%) in Neuro-2a cells (Fig. 4B, left panel). On contrary, expressing 2FP-C-TDP-43 displayed higher E_FRET_ (average E_FRET_ = 28.12%) value in the cytosol (Fig. 4B, middle panel). Since PROTAC **2** treatment alleviated the E_FRET_ of 2FP-C-TDP-43 (average E_FRET_ = 22.4%) (Fig. 4B, right panel), we concluded that PROTAC **2** was capable of inhibiting C-TDP-43 aggregation process.

To focus on lifetime changes of C-TDP-43 oligomeric intermediates, we limited the fitting target from overall C-TDP-43 species (aggregates + oligomers + monomers) to the soluble ones (oligomers + monomers). By setting the threshold of the photon counts, we defined the 2FP-C-TDP-43 species which emitted high photon counts (red pixels) as “aggregated C-TDP-43” (Additional file 1: Fig. S8A, middle panel) and other regions exhibited less photon counts as “soluble C-TDP-43” (Additional file 1: Fig. S8A, right panel). The soluble C-TDP-43 regions (highlighted in purple color) plotted on the phasor space was ready for FD lifetime fitting analysis (Additional file 1: Fig. S8B). By applying 2-exponential lifetime fitting under “highlighted-pixel” model [52], we obtained the averaged lifetime of selected regions, which denoted C-TDP-43 oligomeric intermediates (Additional file 1: Fig. S8C). Subsequently, we converted the averaged lifetime values into E_FRET_ (Details in “Materials and methods” section). According to the FD lifetime fitting data of Additional file 1: Fig. S8C, PROTAC **2** significantly decreased the E_FRET_ of the C-TDP-43 oligomeric intermediates from 48.92% to 40.11% (Fig. 4C). Meanwhile, PROTAC **2** also significantly reduced C-TDP-43 oligomeric intermediates among soluble C-TDP-43 species population (Fig. 4D). To confirm the result of Fig. 4D, we further examined the abundance of C-TDP-43 oligomeric intermediates by size-exclusion chromatography (SEC) along with staining of oligomer-specific antibody (A11). The lysate of eGFP-C-TDP-43 expressed Neuro-2a cells in treatment with PROTAC **2** was fractionated, and then analyzed by slot blot assay with oligomer-specific antibody A11 (Fig. 4E). Using the markers of 670 and 158 kDa, eGFP-C-TDP-43 oligomeric intermediates were shown to occur mostly in fractions 9–11 with relatively high molecular weights (Fig. 4E, upper panel). In contrast, the addition of PROTAC **2** significantly reduced the intensity of A11 signal in fractions 9–11 (Fig. 4E, middle panel), indicating that PROTAC **2** has lowered the content of C-TDP-43 oligomeric intermediates. Again, the addition of both PROTAC **2** and MG132 failed to reduce the content of oligomeric intermediates (Fig. 4E, lower panel). Collectively, our results have verified the dual-targeting capacity of PROTAC **2** against both protein aggregates and oligomers.

### PROTAC 2 degrades C-TDP-43 aggregates and improves the motility of *C. elegans*

**Fig. 5:**
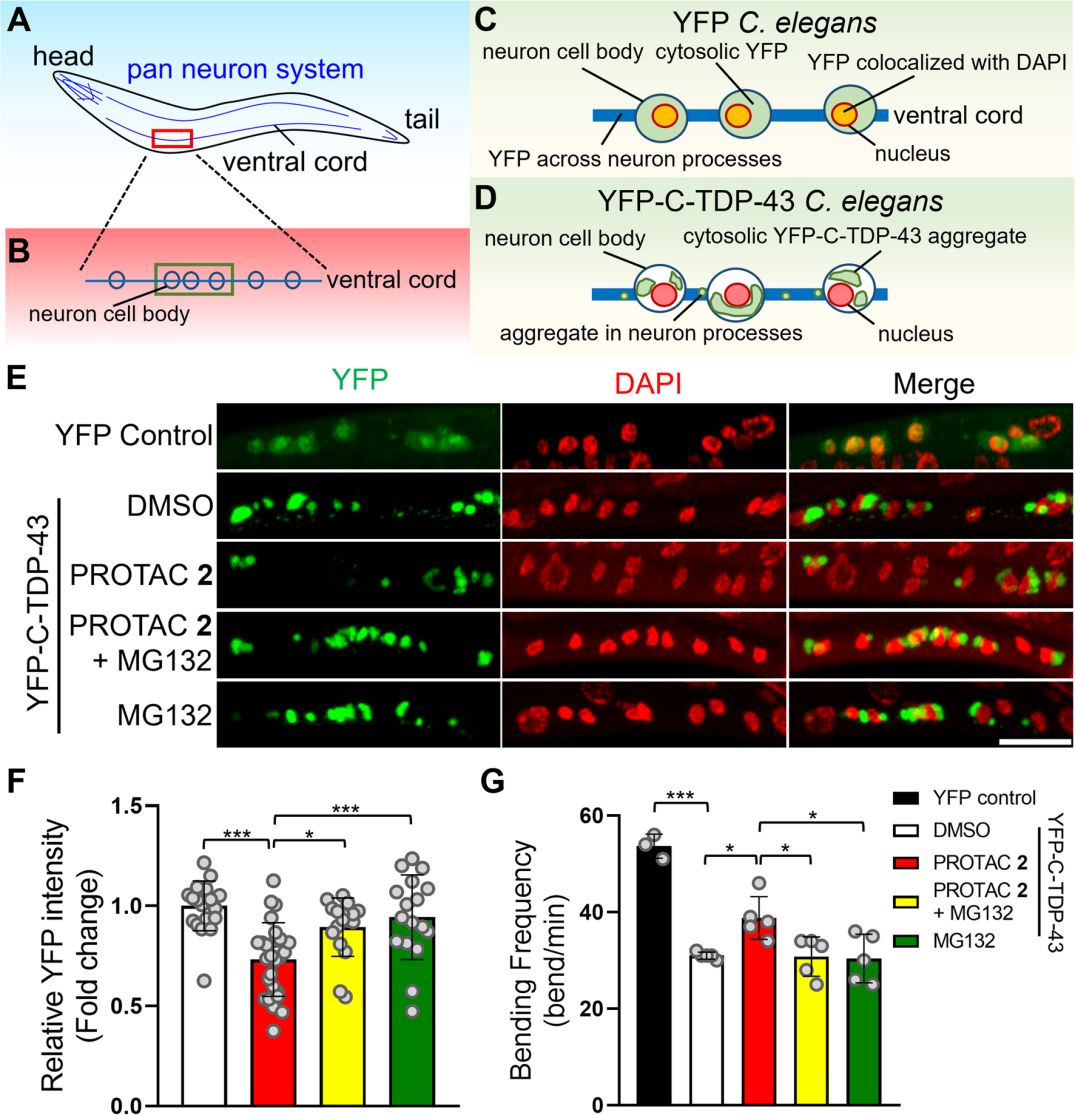
PROTAC **2** reduced C-TDP-43 aggregation and improved the motility of the neuronal YFP-C-TDP-43 transgenic *C. elegans*. **A**, **B** Schematic drawings of neuronally expressing *C. elegans* and its ventral cord. **C**, **D** Illustration of YFP (**C**) and YFP-C-TDP-43 (**D**)expression pattern within the region of interest in panel B. **E** Representative images of either YFP control or YFP-C-TDP-43 transgenic *C. elegans* with or without PROTAC **2** (5 μM) or/and MG132 (5 μM). While the cytosolic aggregates within the ventral cord were visualized in YFP channel (green), the nuclei of neuron cell bodies were monitored in DAPI channel (pseudo red color). Scale bar = 10 μm. **F** The relative YFP intensity of YFP-C-TDP-43 strain in panel E. **G** The bending frequency of YFP-C-TDP-43 transgenic *C. elegans* with or without PROTAC **2** (5 μM) or/and MG132 (5 μM) and YFP control. Each dot represents an independent experiment containing at least 15 worms with three repeat videos. All the statistic results were quantified by ImageJ and shown as mean ± SD (n ≥ 3). Data were analyzed by one-way ANOVA with Tukey post-hoc test (*P < 0.05, ***P < 0.001)

Progressive behavior impairment is the hallmark of ALS pathology [66]. Other than cell culture model, we utilized a *C. elegans* animal model to investigate the potential impact of PROTAC **2**-induced C-TDP-43 aggregates degradation. Because of the well-studied nervous system, accessible genetic manipulation, optical transparency, quantifiable locomotion, *C. elegans* was applied in our initial study for the beneficial effects evaluation [72]. Herein, we employed YFP (hereafter YFP control) and YFP-TDP-43_219–414_ (hereafter YFP-C-TDP-43) transgenic *C. elegans* strains, which ectopically express either YFP alone or the YFP fused with a 25 kDa C-terminal fragment of human TDP-43 in the pan-neuron system [54] (Fig. 5A–D). As shown in Fig. 5E, YFP-C-TDP-43 strain developed cytosolic aggregates with bright fluorescence intensity in the neurons. On the contrary, the YFP control strain displayed weaker fluorescence intensity across the neurons. Phenotypically, the YFP control strain displayed faster swimming movement compared to the YFP-C-TDP-43 strain [73]. To monitor the beneficial effect of PROTAC **2**, we recorded the cytosolic aggregates and kept tracing the thrashing frequency of YFP-C-TDP-43 *C. elegans* in the presence or absence of PROTAC **2**.

Since PROTAC **2** did not affect the larval growth and neuronal cell number in the YFP-C-TDP-43 strain (Additional file 1: Fig. S9A–C), we further characterized the YFP-C-TDP-43 aggregates in the neuronal bodies and processes in the ventral cord to facilitate phenotype scoring. Our data showed that the PROTAC **2-**treated YFP-C-TDP-43 strain exhibited fewer aggregates with significant reduction of YFP fluorescence intensity compared to the DMSO control group or MG132-treated group (Fig. 5E, F). The PROTAC **2-**induced reduction of C-TDP-43 aggregates in neuron bodies was UPS-dependent as YFP-C-TDP-43 aggregates persisted in the combined treatment (PROTAC **2** + MG132). Furthermore, we utilized YFP control strain to confirm the degradation specificity of PROTAC **2**. As shown in Additional file 1: Fig. S9D, E, the YFP control strain displayed consistent fluorescence signal across the neuronal cells in the presence and absence of PROTAC **2**. Conclusively, PROTAC **2** only decreased the cytosolic aggregates in YFP-C-TDP-43 strain (Fig. 5E, F) but not that in YFP control strain (Additional file 1: Fig. S9D, E).

Furthermore, we examined the effect of PROTAC **2** on relieving the locomotive defects of YFP-C-TDP-43 strain by monitoring *C. elegans* thrashing in buffer as a phenotypical assessment of the drug efficacy (Fig. 5G, Additional file 2, Additional file 3 and Additional file 4). We observed that the YFP-C-TDP-43 strain (Fig. 5G, white bar) exhibited severe locomotive defect compared to YFP control strain (Fig. 5G, black bar). This phenomenon may indicate that the neuronal toxicity mainly results from the C-TDP-43 aggregates. Thus, treatment of PROTAC **2** significantly increased the bending frequency of YFP-C-TDP-43 strain by 25.1% (Fig. 5G, red bar) compared to the DMSO and MG132-treated groups. The beneficial effect of PROTAC **2** diminished in the combined treatment with MG132 (Fig. 5G, yellow bar). Our study revealed that PROTAC **2** has remarkable effects in reducing C-TDP-43 aggregates and improving the motility of the C-TDP-43 transgenic *C. elegans*.

## Discussion

PROTAC technology is known for inducing targeted-protein degradation and has been utilized in the field of cancer, immunity, and virus studies [7–17]. Recently, it has also been applied for the degradation of misfolding proteins, which has long been considered as undruggable by inhibitors, agonists, or antagonists in conventional therapeutic strategies [9, 17, 29–31]. As demonstrated in this study, we have successfully designed a series of PROTACs against the misfolding TDP-43 in neuronal cells and *C. elegans* to evaluate their efficacy in relieving cytotoxicity by inducing degradation of target protein. PROTAC **2** was constructed by three components, including pomalidomide (POM) as a CRBN binder, a benzothiazole-aniline (BTA) as a binder of C-TDP-43 protein in β-sheet structures, and a tetraethyleneglycol (TEG) linker to promote formation of the POM–TEG–BTA tertiary complex and thus enable degradation of C-TDP-43 aggregates via ubiquitin proteasome system. Compared to the positively charged thioflavin T (ThT), the electrically neutral BTA may have better cell permeability [46] and higher binding affinity [58] to amyloid aggregates and fibrils, which are often found in neurodegenerative diseases. In addition to the PEG-linked PROTACs, we also synthesized POM–BTA compounds (e.g. **S13**) having aliphatic linkers (Supplementary Scheme S3) and a lenalidomide–BTA compound **S18** that is connected by a triazole-containing linker (Supplementary Scheme S4). However, these compounds rendered low water solubility as the hydrophobicity of linkers increased, and their use as PROTACs was not further examined.

During the PROTACs design and evaluation process, we found the length of linker was correlated with the degradation ability and cytotoxicity. Generally, the linker should be long enough to avoid steric hindrance between the target protein and E3 ligase, while the linker cannot be too long to cause futile transfer of ubiquitins [24]. Zorba and coworker have shown that the PROTACs with longer PEG linkers exhibit higher binding ability to CRBN and promote formation of the ternary complex [74]. However, some PROTACs with shorter linkers still have potent efficiency [75, 76]. During our optimizing process, we found the candidate with the shortest linker (PROTAC **1**) significantly lost its TDP-43-degrading capability (Fig. 2B). It was hypothesized that PROTAC **1** may already lose its ability to form the ternary complex with the target protein and E3 ligase. Increasing the length of the linker further increased the degradation efficiency against TDP-43 aggregates as shown in the case of PROTAC **2–4**. However, we had also shown that the cytotoxicity was positively correlated with the linker length (PROTAC **3** and **4**). Taken together, optimizing the length of linker is essential for the development of PROTACs in order to gain the potent efficacy and drug safety.

Though PROTAC is designed for targeted protein degradation by hijacking the endogenous E3 ligase and ubiquitin proteasome system (UPS), unpredictable side effect of PROTAC may sometimes occur by occupying endogenous E3 ligase [77, 78]. Another possible side effect of PROTAC could result from unpredictable off-targeting degradation. In the reported cases of PROTAC-CRBN binary complex, some possible off-targeting proteins (*e.g.* GSPT1) have just been identified [79]; however, we found that PROTAC **2** did not induce GSPT1 off-targeting degradation (Additional file 1: Fig. S2C). In the future, structural modification of PROTAC **2** and computational compounds screening could be carried out to reduce the possible side effect.

Although PROTACs appear to have many advantageous features for potential therapeutic uses, there are still many obstacles to be overcome, such as pharmacokinetics in the human body and blood–brain-barrier (BBB) penetration for treatment of CNS diseases. Lipinski’s rule of five (Ro5) [80] has been widely used to predict the pharmacokinetics of drug molecules. However, whether Ro5 can be applied to PROTACs is still not convincing. A recent report [81] indicated that XL01126, a PROTAC degrader of leucine-rich repeat kinase 2 (LRRK2), could penetrate the BBB regardless of its unfavorable in vitro pharmacokinetics and violation of Ro5 and/or RoCNS [82]. Some studies also revealed that PROTACs even with high molecular mass could still cross the blood–brain barrier to induce the degradation of target proteins in brain regions [29, 83, 84]. For example, C004019 is a PROTAC having a molecular mass of 1035 Dalton; however, subcutaneous administration of C004019 was effective to decrease tau levels in the brains of mice [84]. Although each PROTAC was made for thorough degradation of a specific target, the possible cytotoxicity caused by off-targeting should be closely monitored along the drug development.

It is also worthy to note that though PROTAC **2** significant reduced C-TDP-43 aggregates, the beneficial effect on C-TDP-43 cytotoxicity in Neuro-2a and motor neuron defects in *C. elegans* were relatively minor. This disparity between degradation efficiency and beneficial results may arise from the loss of function of TDP-43 [85]. Increasing evidence has suggested overexpressing of N-terminally truncated TDP-43 caused nuclear clearance of the endogenous TDP-43, which resulted in cytotoxicity accompanied with neuronal death [86]. Conclusively, extensive nuclear clearance of TDP-43 driven by the C-TDP-43 expressing system may lessen the beneficial effect of PROTAC **2** treatment. Furthermore, while PROTAC **2** has been reported to degrade the misfolding protein aggregates, we provided new evidence to demonstrate PROTAC **2** could decrease both the amount and the compactness of cytosolic oligomers synchronously. As progressive transition of aggregation reflects the intrinsic energy states in aberrant amyloid protein [87], reduction of E_FRET_ of C-TDP-43 oligomeric intermediate in the aforementioned experiment suggested PROTAC **2** could also decrease the intrinsic energy state and interfere with the conformation of C-TDP-43 oligomers. The similar case has been reported in the other amyloid protein binding peptides (*i.e.* peptides against mutant Huntingtin protein). [52]

As far as we know, PROTAC **2** is the first case which can effectively degrade C-TDP-43 oligomers in addition to its protein aggregates, which provides an alternatively therapeutic strategy against TDP-43 proteinopathy in ALS. To further examine the detailed therapeutic effects of PROTAC **2**, the advanced studies of other animal models, including rodents with the mutant TDP-43 aggregates [88, 89], would be considered.

## Conclusions

**Fig. 6:**
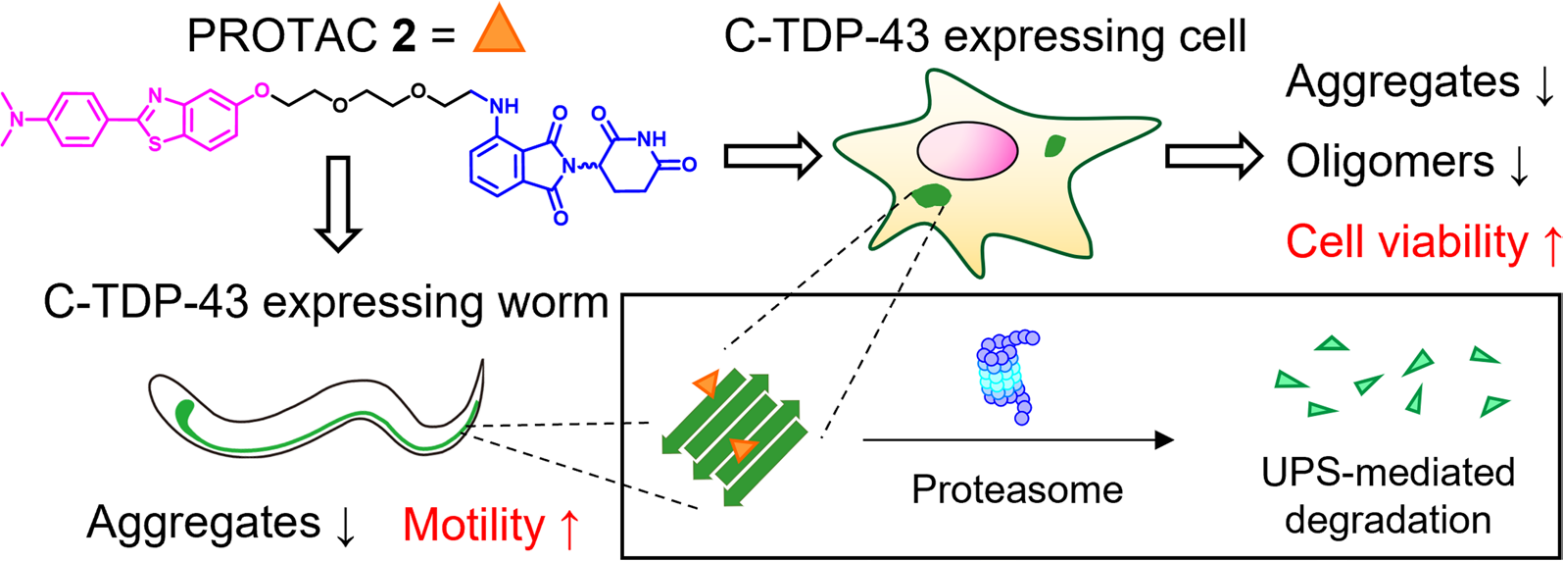
A schematic summary of the results. PROTAC **2** reduced C-TDP-43 aggregation and oligomerization, increased viability in the cell model, and improved the motility of the neuronal C-TDP-43 transgenic* C. elegans*

Herein, we demonstrated that PROTAC **2** (JMF4560) significantly reduced C-TDP-43 aggregates and alleviated C-TDP-43-induced toxicity through the proteasomal degradation (Fig. 6). This degradation occurred without affecting endogenous full-length TDP-43. By applying transgenic *C. elegans*, we also observed that PROTAC **2** was capable of reducing C-TDP-43 aggregates in the nervous system and exhibited beneficial effect on its motility (Fig. 6). Furthermore, we revealed that PROTAC **2** could reduce the compactness of C-TDP-43 oligomeric intermediates and decrease their population. By demonstrating the efficiency of our newly-designed PROTACs on the degradation of C-TDP-43 aggregates and oligomers, we wish to develop these small molecules as new drugs against misfolding proteins in neurodegenerative diseases.

## Supplementary Information

**Additional file 1**. Figures S1–S9, synthetic schemes S1–S4, synthetic procedures and characterization of compounds, 1H and 13C NMR, absorption, and fluorescence spectra, and HPLC diagrams.

**Additional file 2**. Video of YFP transgenic C. elegans during motility assay.

**Additional file 3**. Video of YFP-C-TDP-43 transgenic C. elegans during motility assay.

**Additional file 4**. Video of YFP-C-TDP-43 transgenic C. elegans upon PROTAC 2 treatment during motility assay.

## Data Availability

The datasets used and/or analyzed during the current study are available from the corresponding author on reasonable request.

## Abbreviations

*ALS*: Amyotrophic lateral sclerosis
*BBB*: Blood–Brain Barrier
*BTA*: Benzothiazole-aniline
*CA*: Cellulose acetate
*cIAP*: Cellular inhibitor of apoptosis protein
*CNS*: Central nervous system
*CRBN*: Cereblon
*C-TDP-43*: C-terminal TDP-43
*DDB1*: DNA damage-binding protein 1
*E*_*FRET*_: FRET efficiency
*FLIM*: Fluorescence lifetime imaging microscopy
*2FP-C-TDP-43*: Co-expressing eGFP-C-TDP-43 and mCherry-C-TDP-43
*FTLD*: Frontotemporal lobar degeneration
*GSPT1*: G1 To S Phase Transition 1
*MDM2*: Murine double minute 2
*PROTACs*: Proteolysis targeting chimeras
*PEG*: Polyethylene glycol
*Pan-*: All (specially used in *C. elegans*)
*SEC*: Size-exclusion chromatography
*TDP-43*: TAR DNA-binding protein 43
*ThT*: Thioflavin T
*TR-FRET*: Time-resolved fluorescence resonance energy transfer
*UPS*: Ubiquitin proteasome system
*VHL*: Von Hippel-Lindau
