# Supplementary material for: Degradation of neurodegenerative disease-associated TDP-43 aggregates and oligomers via a proteolysis-targeting chimera
Source: J Biomed Sci. 2023 Apr 26;30:27. doi: 10.1186/s12929-023-00921-7 (PMC10131537; doi:10.1186/s12929-023-00921-7)
Supplement: Supplementary file 1 — Additional file 1. Figures S1–S9, synthetic schemes S1–S4, synthetic procedures and characterization of compounds, 1H and 13C NMR, absorption, and fluorescence spectra, and HPLC diagrams. [file 12929_2023_921_MOESM1_ESM.docx]

**Additional file 1**

**Degradation of Neurodegenerative Disease-associated TDP-43 Aggregates and Oligomers via a Proteolysis-Targeting Chimera**

Yu-Ling Tseng,^†,§^ Po-Chao Lu,^‡,‖,⊥,§^ Chi-Chang Lee,^‡^ Ruei-Yu He,^‡^ Yung-An Huang,^‡^ Ying-Chen Tseng,^&^ Rachel Ting-Jen Cheng,^&^ Joseph Jen-Tse Huang,^‡, ⁋,#,^ ^∇,^* and Jim-Min Fang^†,^*

| **Contents** | **Pages** |
| --- | --- |
| Figure S1. Examination on cytotoxicity and potential regulatory mechanism of PROTAC molecules in Neuro-2a cells. | S2–S3 |
| Figure S2. Examination on the potential degradation target of PROTAC molecules in Neuro-2a cells. | S4 |
| Figure S3. Measuring the binding affinity between PROTACs **1–4** and cereblon. | S5 |
| Figure S4. The interaction between PROTAC **2** and C-TDP-43 aggregates. | S6-S7 |
| Figure S5. Linker–BTA and linker–POM failed to decrease insoluble C-TDP-43 aggregates. | S8 |
| Figure S6. Respective expressivity of eGFP, mCherry, eGFP-C-TDP-43, and mCherry-C-TDP-43 in Neuro-2a cells. | S9 |
| Figure S7. Fluorescent PROTAC **2** exhibited neglect crosstalk with eGFP donor lifetime. | S10 |
| Figure S8. Lifetime analysis of C-TDP-43 oligomeric intermediates in Neuro-2a cells. | S11 |
| Figure S9. The growing rate and YFP fluorescence signal remained stable upon 5 µM PROTAC 2 treatment in the transgenic *C. elegans*. | S12–S13 |
| Scheme S1. Synthesis of compounds **9a**–**9d** and PROTACs **1–4** | S14 |
| Scheme S2. Synthesis of compound **S8** (JMF4576), a POM-PEG derivative | S14 |
| Scheme S3. Synthesis of POM–BTA PROTACs having linkers of aliphatic chains | S15 |
| Scheme S4. Synthesis of LEN–BTA PROTAC with a triazole-containing linker | S15 |
| Synthetic procedures and characterization of compounds | S16–S30 |
| Supplementary References | S31 |
| ^1^H, ^13^C and ^19^F NMR, absorption, fluorescence spectra and HPLC diagrams | S32–S64 |


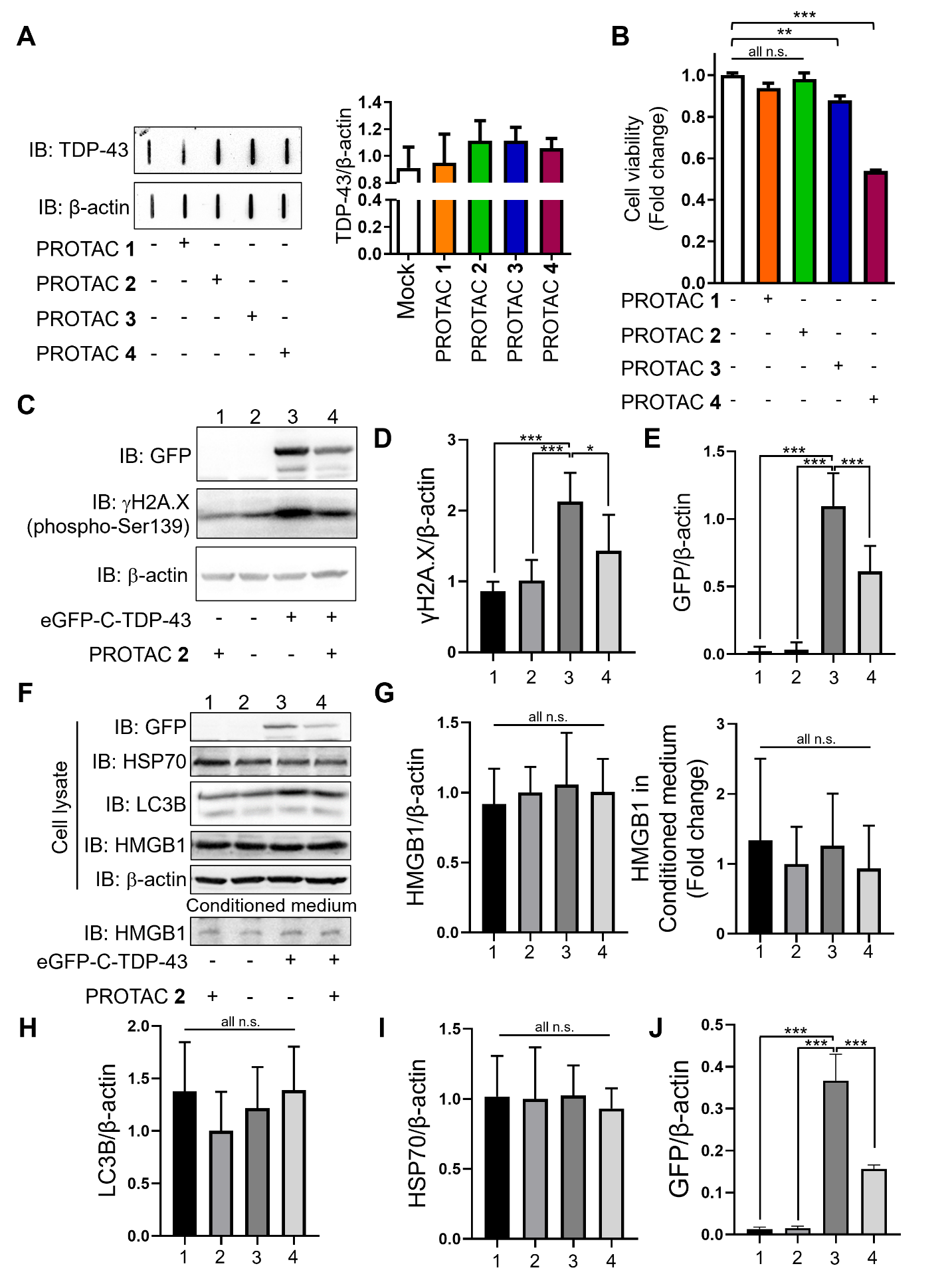


**Figure S1. Examination on cytotoxicity and potential regulatory mechanism of PROTAC molecules in Neuro-2a cells.** (A) Slot blot assay (left) examining on the protein among of endogenous TDP-43 (probed with TDP-43 antibody) in PROTACs **1**–**4** (5 μM) treated Neuro-2a cells accompanied with the quantification figure (right). The protein level of endogenous TDP-43 was normalized with β-actin (loading control). (B) AlamarBlue assay of PROTACs **1**–**4** (5 μM) treated Neuro-2a cells. (C) Western blot of eGFP-C-TDP-43 transfected Neuro-2a cells upon treatment of PROTAC **2** at 5 μM concentrations. (D-E) The quantification of blots in panel C showed the ratio of probed protein (GFP for eGFP-C-TDP-43, γH2A.X as an apoptosis-associated marker) to the loading control (β-actin). (F) Western blot of eGFP-C-TDP-43 transfected Neuro-2a cells upon treatment of PROTAC **2** at 5 μM concentrations. (G-J) The quantification of blots in panel F showed the ratio of probed protein (GFP for eGFP-C-TDP-43, HSP70 regulates TDP-43 protein re-folding, HMGB1 as a necrosis marker, and LC3B as an autophagy marker) to the loading control (β-actin). Statistic results are shown as mean ± SD (n ≥ 3). The slot blot assay and viability data were analyzed by one-way ANOVA with Dunnett post-hoc test (***P* < 0.01, ****P* < 0.001). The western blotting data were analyzed by one-way ANOVA with Tukey post-hoc test (**P* < 0.05, ****P* < 0.001).


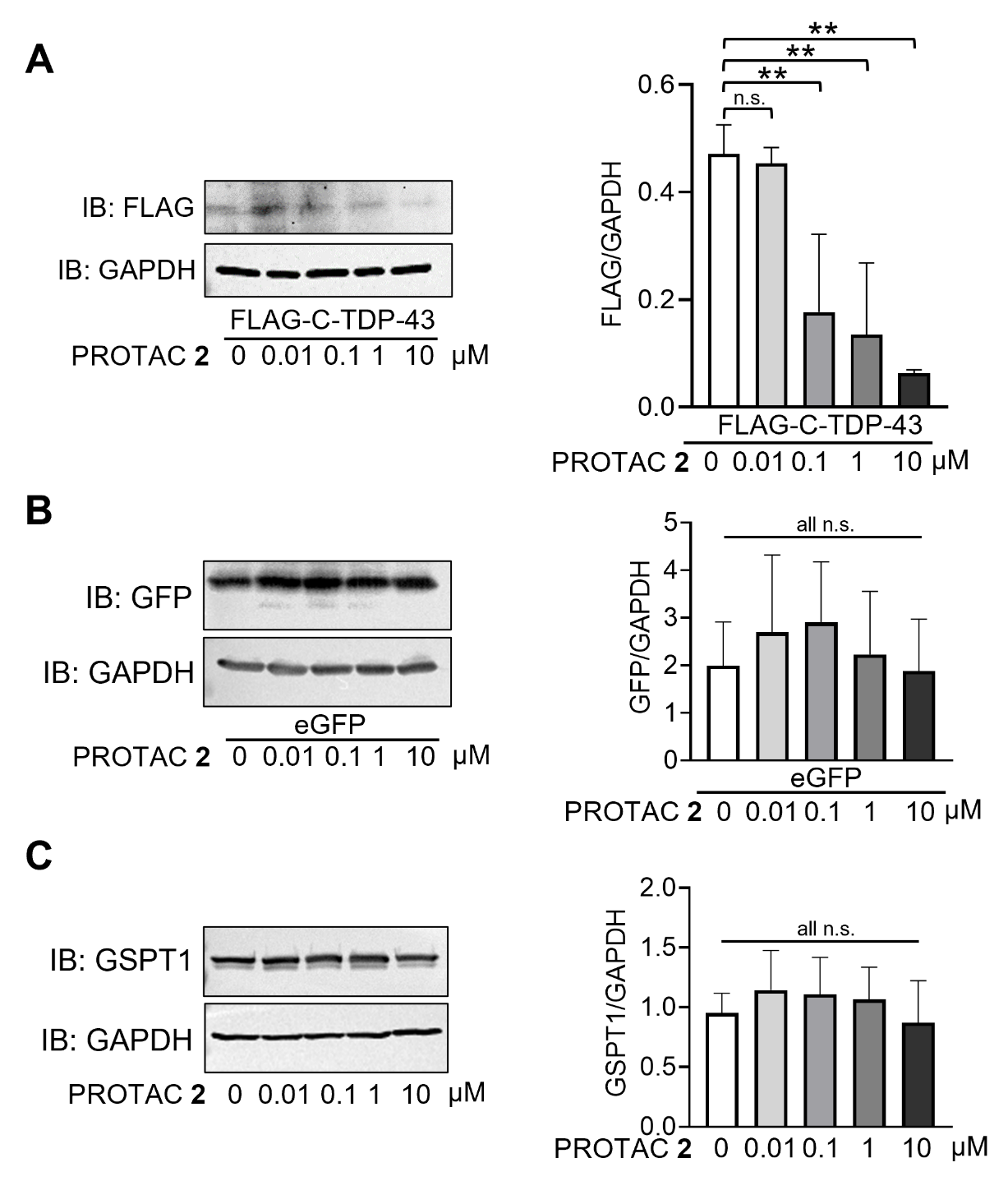


**Figure S2. Examination on the** **potential degradation target of PROTAC molecules in Neuro-2a cells.** (A-C) Western blot of FLAG-C-TDP-43 transfected (A), eGFP transfected (B), or none transfected (C) Neuro-2a cells upon treatment of PROTAC **2** at various concentrations (left panels). The quantification of blots (right panels) in panel A, B, and C showed the ratio of FLAG-C-TDP-43 aggregates (flag M2 antibody), eGFP fluorophore (GFP antibody), and GSPT1 protein (GSPT1 antibody) to GAPDH (loading control), respectively. Statistic results are shown as mean ± SD (n ≥ 3). Data were analyzed by one-way ANOVA with Dunnett post-hoc test (***P* < 0.01).

**
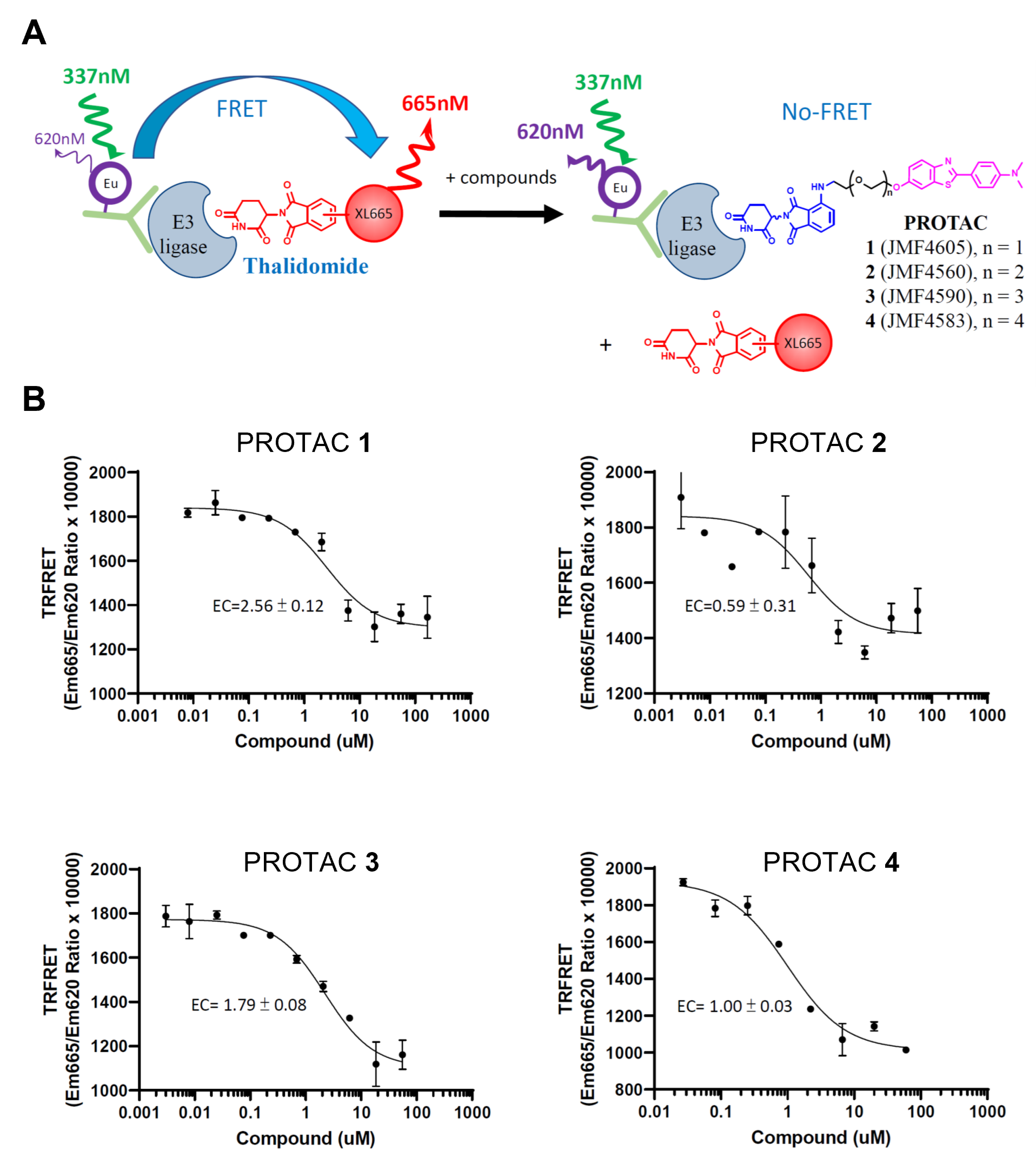
**

**Figure S3**. **Measuring the binding affinity between PROTACs 1–4 and cereblon.** (A) The binding to E3 ligase (cereblon) is monitored by time-resolved fluorescent resonance energy transfer. The assay employs XL665-conjugated thalidomide and europium cryptate-conjugated glutathione-*S*-transferase (GST) antibody that can bind to GST-tagged cereblon protein. Once the complex forms, the emission light at 620 nm by europium cryptate is absorbed by XL665, which emits at 665 nm. Upon the presence of other E3 ligase binders (PROTACs **1**–**4**), XL665-conjugated thalidomide is released and thus the emission light at 620 nm by europium cryptate can be detected. (B) The dose-dependent response of pomalidomide (as a control) and PROTACs **1**–**4** to E3 ligase binding. The ratio of the acceptor (XL665, 665 nm) and the donor (europium-cryptate, 620 nm) emission signals were used for calculation of EC_50_ values by using a nonlinear fit model (GraphPad Prism Software). Data are presented as means ± standard deviation (*n* = 3).


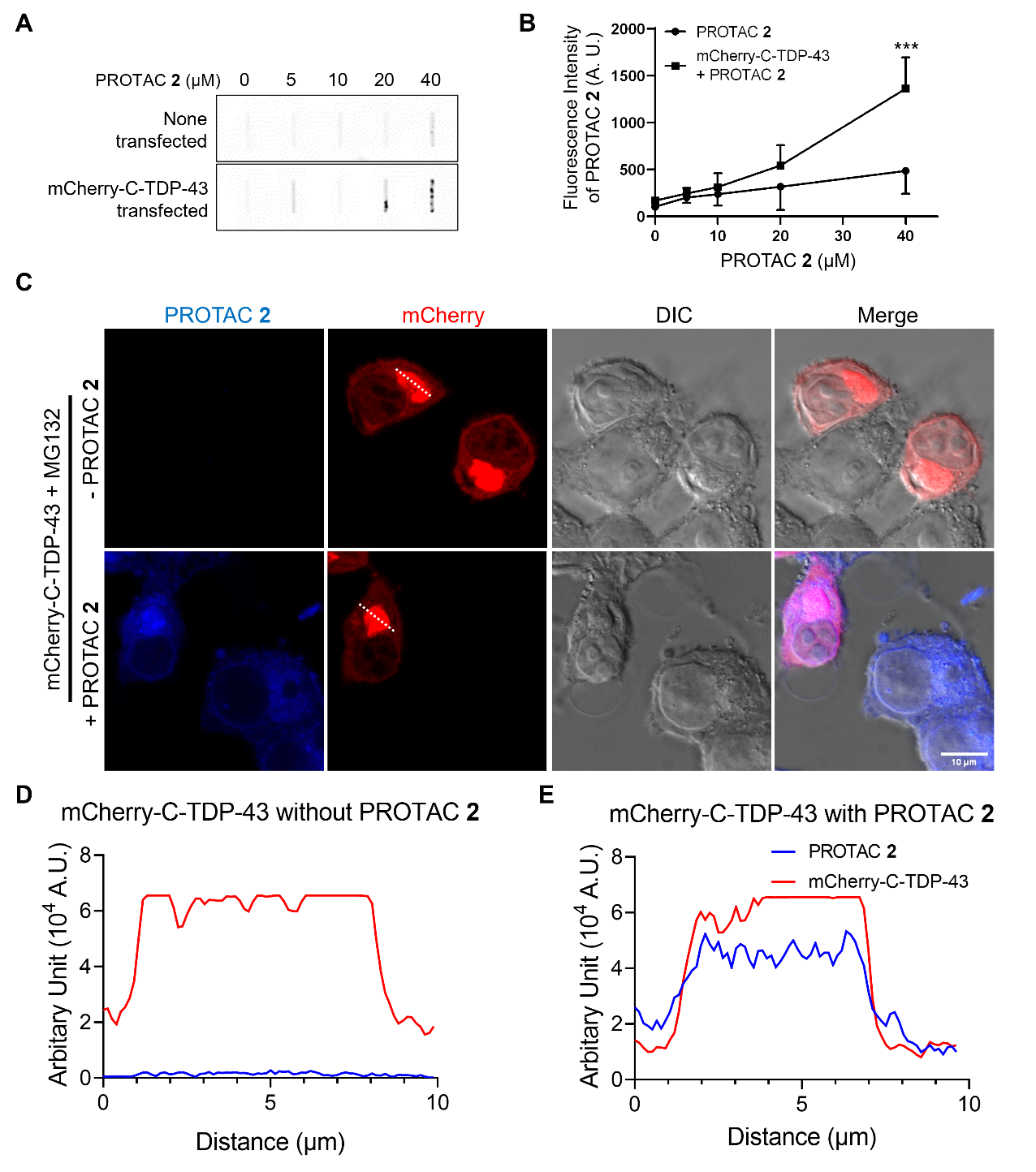


**Figure S4. The interaction between PROTAC 2 and C-TDP-43 aggregates.** (A) *In vitro* binding assay showing the binding event between PROTAC **2** and mCherry-C-TDP-43 aggregates. In this assay, mCherry-C-TDP-43 aggregates were firstly mixed to various concentration of PROTAC **2** and subsequently retained on the CA membrane using filter trap assay. The signal of PROTAC **2** was visualized by applying Typhoon9410 Variable Mode Imager. (λ_ex_ = 457 nm, λ_em_ = 488 nm) (B) The quantification of blots in A. Statistic results are shown as mean ± SD (n ≥ 3). Data were analyzed by two-way ANOVA with False Discovery Rate post-hoc test (*** *P* < 0.001). (C) Confocal images of mCherry-C-TDP-43 aggregates and PROTAC **2** distribution in Neuro-2a cells with or without 5 µM PROTAC **2** (14 hr after MG132 pretreatment). To facilitate the visualization of the TDP-43 aggregates, MG132 (2 μM, 2 hr after transfection) was pretreated in the both conditions. (D) Intensity profiles of mCherry-C-TDP-43 (red) along the dashed lines (mCherry channel) in upper panel C. (E) Intensity profiles of PROTAC 2 (blue) and mCherry-C-TDP-43 (red) along the dashed lines (mCherry channel) in lower panel C.


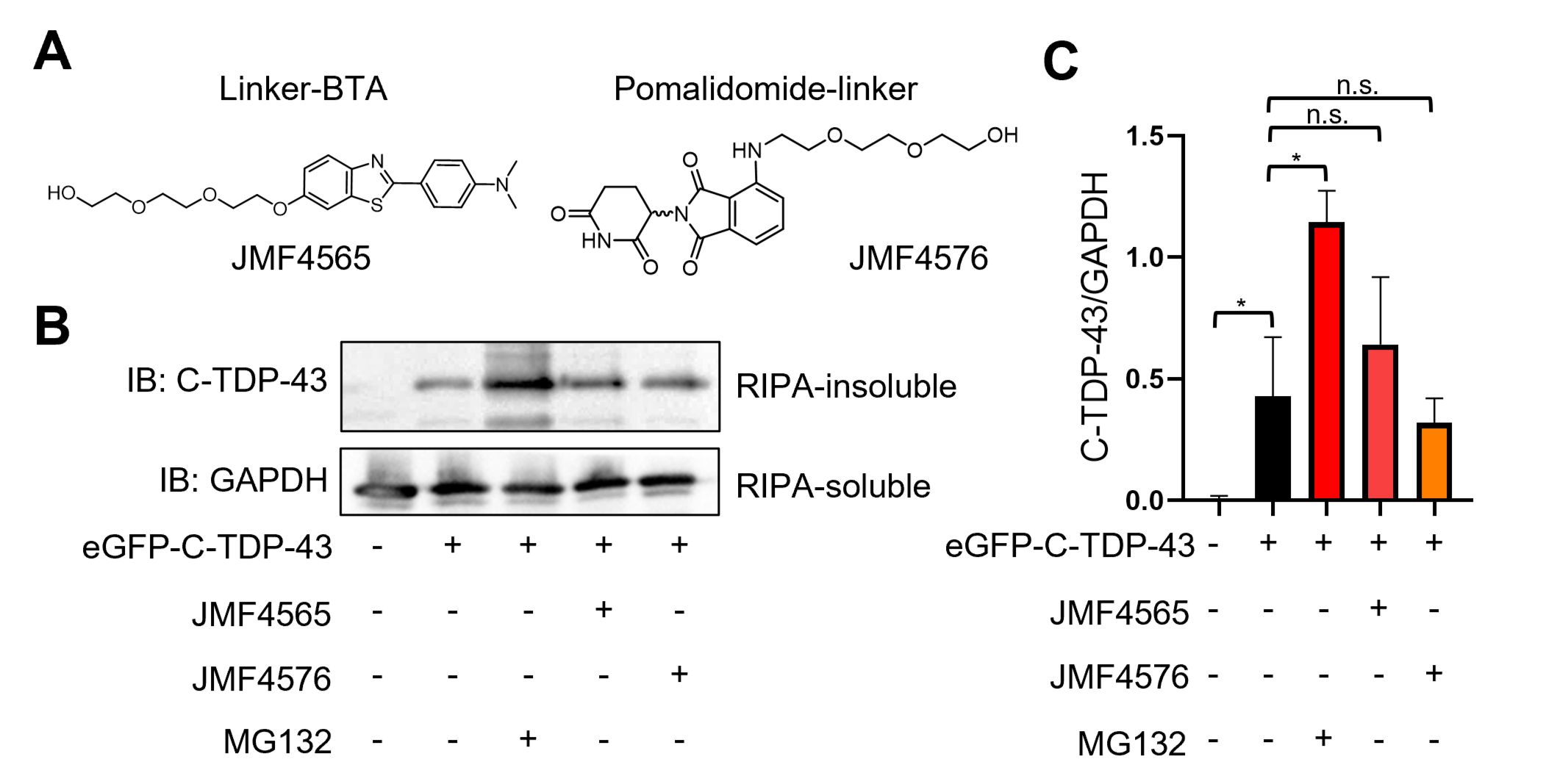


**Figure S5. Linker-BTA (JMF4565) and linker-pomalidomide (JMF4576) failed to decrease insoluble C-TDP-43 aggregates.** (A) Molecular structures of JMF4565 (compound **S5a**) and JMF4576 (compound **S8**). (B) Western blot of eGFP-C-TDP-43 transfected Neuro-2a cells upon MG132 (2 μM), JMF4565 (linker-BTA, 5 μM), or JMF4576 (linker-pomalidomide, 5 μM) treatment. The data were demonstrated by SDS-PAGE and probed with TDP-43 (C-terminal) antibody and GAPDH antibody (loading control). (C) Quantification of blots in panel B. Statistic results are shown as mean ± SD (n ≥ 3). Data were analyzed by one-way ANOVA with Dunnett post-hoc test (**P* < 0.05).


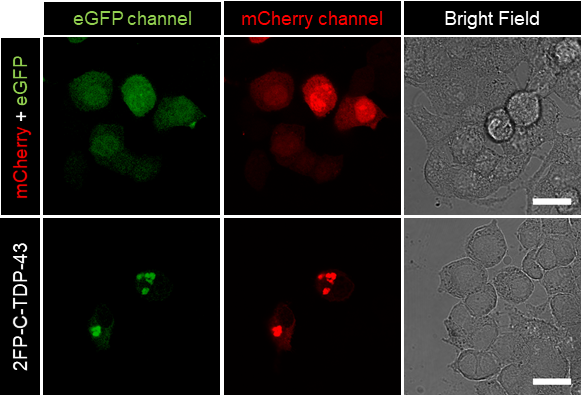


**Figure S6. Respective expressivity of eGFP, mCherry, eGFP-C-TDP-43, and mCherry-C-TDP-43 in Neuro-2a cells.** Representative images of Neuro-2a cells co-expressing either (mCherry + eGFP) or eGFP-C-TDP-43 + mCherry-C-TDP-43 (2FP-C-TDP-43) as visualized by confocal microscopy. Both eGFP-C-TDP-43 and mCherry-C-TDP-43 share similar expressivity and aggregation-prone properties. Scale bar = 10 μm.


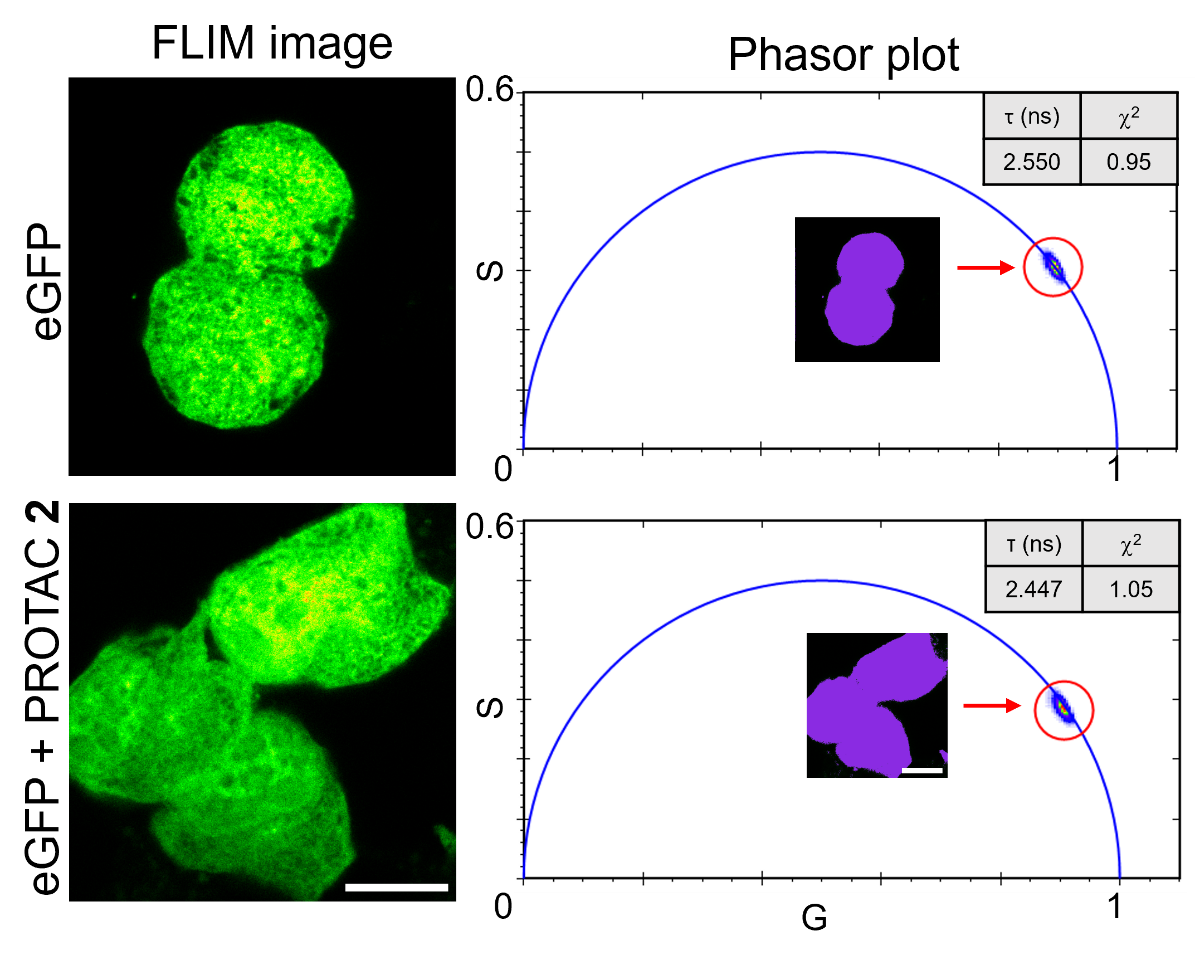


**Figure S7. PROTAC 2 exhibited negligible lifetime crosstalk with eGFP donor.** To rule out the possible lifetime crosstalk between eGFP donor and fluorescent PROTAC **2**, we checked the lifetime of eGFP alone and eGFP + PROTAC **2** in Neuro-2a cells. Left panel: representative FLIM images of eGFP transfected Neuro-2a cells with or without PROTAC **2** treatment. Right panel: the eGFP lifetime corresponding to the pixels of FLIM images were plotted on the phasor plot. Similar location of the highlighted pixels indicated that both groups displayed similar eGFP lifetime. Scale bar = 10 μm.


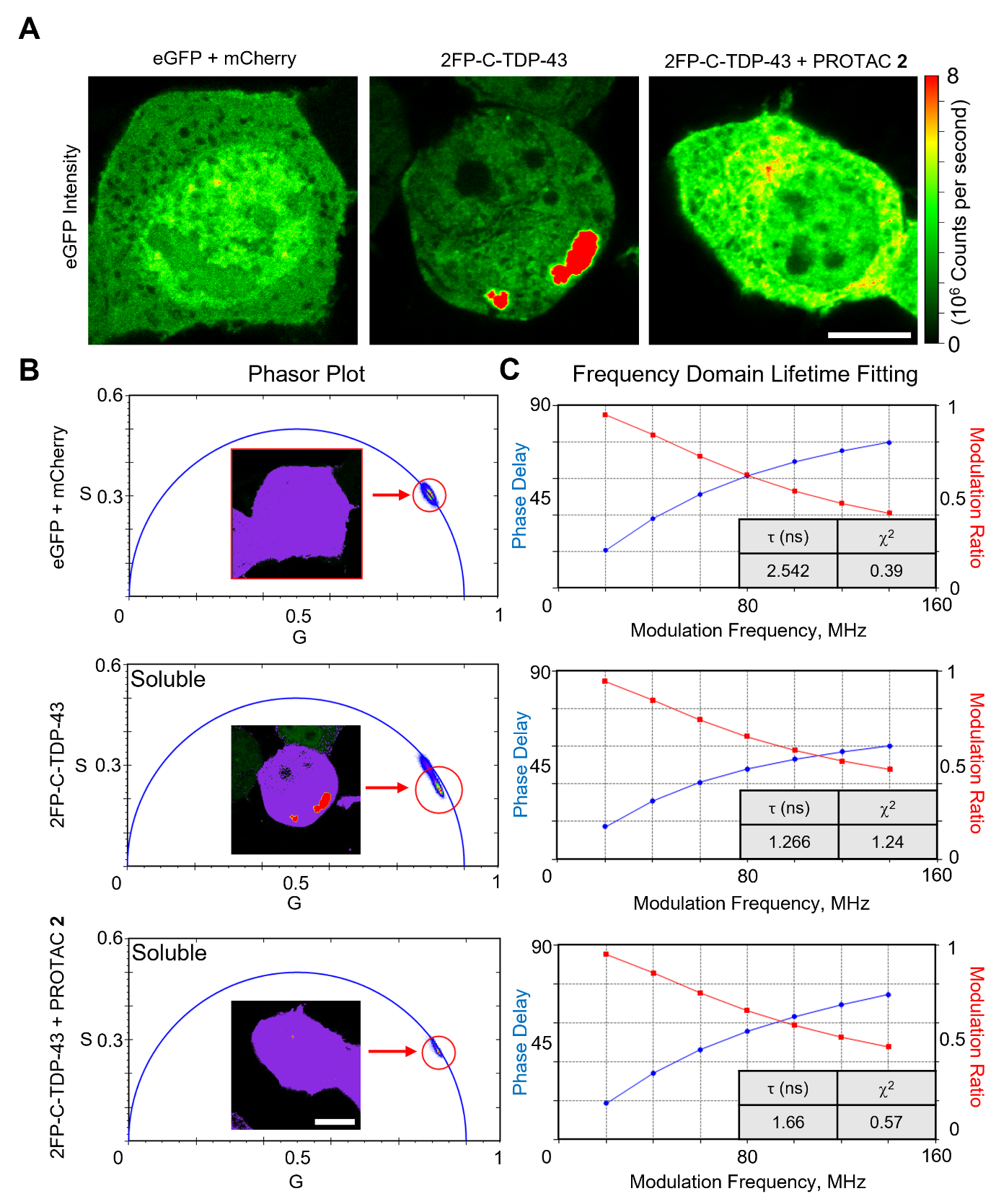


**Figure S8. Lifetime analysis of oligomeric intermediates in Neuro-2a cells.** (A) The color-coded FLIM image of photon counts (counts per second, CPS) distribution throughout Neuro-2a cells. The aggregated C-TDP-43 is shown in red. (B) Soluble C-TDP-43 species shown in panel A were selected (highlighted in purple) and further plotted on phasor plot corresponding to the lifetime. (C) Purple highlighted region from panel B was fitted by applying “highlighted-pixel” model with two-exponential fitting. 2FP-C-TDP-43 represents co-expressing eGFP-C-TDP-43 and mCherry-C-TDP-43. Scale bar = 10 μm.


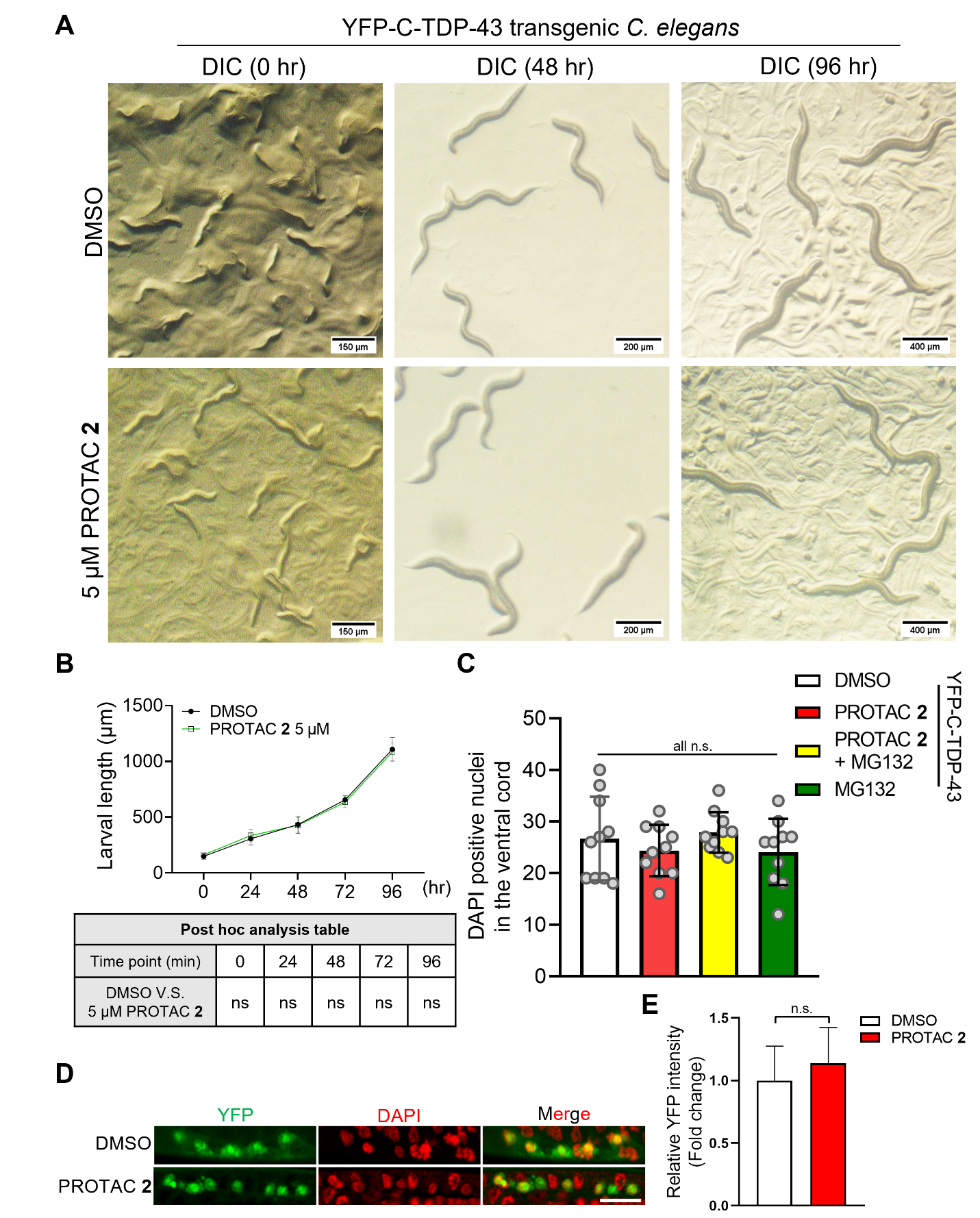


**Figure S9. The growing rate and YFP fluorescence signal remained stable upon 5 µM PROTAC 2 treatment in the transgenic *C. elegans*.** (A) The representative bright field images of *C. elegans* upon 5 μM PROTAC **2** treatments at 0, 48, and 96 hr time points. (B) The quantification of larval length in A. Statistic results are shown as mean ± SD (n ≥ 45). Data were analyzed by two-way ANOVA. ns is not significant. (C) The quantification of neuronal cells (DAPI positive nuclei) in the ventral cord region of the YFP-C-TDP-43 transgenic *C. elegans*. Statistic results are shown as mean ± SD (n ≥ 10). Data were analyzed by one-way ANOVA. (D) The confocal images of YFP *C. elegans* in the ventral cord. Instead of forming cytosolic aggregates, YFP fluorophore stayed in both cytosol and nucleus [colocalized with DAPI channel (pseudo red color)] of neuron bodies according to the YFP channel. Scale bar = 10 μm. (E) No significant change in the relative YFP intensity of the L4 stage *C. elegans* with or without treatment of PROTAC **2**. The concentration of PROTAC **2** was 5 μM. Statistic results are shown as mean ± SD (n ≥ 3). Data were analyzed by two-tailed unpaired t-test.

**Scheme S1.** Synthesis of compounds **9a**–**9d** and PROTACs **1–4**.

**Scheme S2.** Synthesis of compound **S8** (JMF4576), a POM-PEG derivative.

**Scheme S3.** Exemplified synthesis of POM–BTA compounds having linkers of aliphatic chains.

**Scheme S4.** Synthesis of lenalidomide–BTA compound with a triazole-containing linker.

**Synthetic procedures and characterization of compounds**

**2-Bromo-6-methoxybenzo[*d*]thiazole (5)^[S1]^**

Copper bromide (5.58 g, 25 mmol) and *tert*-butyl nitrite (2.96 mL, 25 mmol) were added to a solution of 2-amino-6-methoxybenzothiazole (3 g, 16.6 mmol) in anhydrous acetonitrile (75 mL) under an atmosphere of argon. The mixture was stirred at 65 °C for 1.5 h, and then concentrated under reduced pressure. The mixture was diluted with CH_2_Cl_2_, and washed successively with 1 M HCl_(aq)_, H_2_O, and brine. The organic phase was dried over MgSO_4_, filtered, and concentrated under reduced pressure to give bromo compound **5** (3.54 g, 14.5 mmol, 87% yield). C_8_H_6_BrNOS; brown solid; mp 47.5–49.0 °C; TLC (EtOAc/hexane = 1:2) *R_f_* = 0.7; IR ν_max_ (neat) 2924, 2852, 1602, 1486, 1456, 1436, 1425, 1271, 1258, 980 cm^–1^; ^1^H NMR (400 MHz, CDCl_3_) δ 7.76 (d, *J* = 8.9 Hz, 1 H), 7.14 (d, *J* = 2.6 Hz, 1 H), 6.97 (dd, J = 8.9, 2.6 Hz, 1 H), 3.84 (s, 3 H); ^13^C NMR (100 MHz, CDCl_3_) δ 157.9, 146.6, 138.5, 135.2, 123.1, 115.6, 103.5, 55.7. ESI-HRMS calcd for C_8_H_7_NOSBr^79^: 243.9432, found: *m/z* 243.9455 [M + H]^+^. C_8_H_7_NOSBr^81^: 245.9432, found: *m/z* 245.9437 [M + H]^+^.

**4-(6-Methoxybenzo[*d*]thiazol-2-yl)-*N,N*-dimethylaniline (7)^[S2]^**

A mixture of bromo compound **5** (100 mg, 0.41 mmol), 4-(dimethylamino)phenylboronic acid (82 mg, 0.50 mmol) and K_2_CO_3_ (373 mg, 2.7 mmol) in anhydrous DMF (2 mL) was bubbled with argon for 1 h, followed by addition of Pd(dppf)Cl_2_∙CH_2_Cl_2_ (22 mg, 0.027 mmol). The mixture was stirred at 80 °C for 23 h, and concentrated under reduced pressure. The mixture was extracted with CH_2_Cl_2_ and H_2_O. The organic phase was dried over MgSO_4_, filtered, concentrated under reduced pressure, and purified by flash chromatography on a silica gel column with elution of CH_2_Cl_2_/hexane (3:1) to give compound **7** (70 mg, 60% yield). C_16_H_16_N_2_OS; brown solid; mp 179.5–181.5 °C; TLC (CH_2_Cl_2_/hexane = 3:1) *R_f_* = 0.38; IR ν_max_ (neat) 2920, 2849, 1609, 1495, 1464, 1264, 1225, 818 cm^–1^; ^1^H NMR (400 MHz, CDCl_3_) δ 7.88 (d, *J* = 8.6 Hz, 2 H), 7.85 (d, *J* = 9.0 Hz, 1 H), 7.29 (d, *J* = 2.5 Hz, 1 H), 7.02 (dd, *J* = 9.0, 2.5 Hz, 1 H), 6.72 (d, *J* = 8.6 Hz, 2 H), 3.85 (s, 3 H), 3.01 (s, 6 H); ^13^C NMR (100 MHz, CDCl_3_) δ 166.4, 157.1, 151.9, 148.8, 135.7, 128.5 (2 ×), 122.6, 121.5, 114.9, 111.7 (2 ×), 104.3, 55.7, 40.1(2 ×). ESI-HRMS calcd for C_16_H_17_N_2_OS: 285.1062, found: *m/z* 285.1064 [M + H]^+^.

**2-(4-(Dimethylamino)phenyl)benzo[*d*]thiazol-6-ol (8)^[S3]^**

A solution of compound **7** (1.4 g, 4.9 mmol) in anhydrous CH_2_Cl_2_ (75 mL) was added BBr_3_ (5.61 mL, 60 mmol) dropwise at 0 °C under an atmosphere of argon. The mixture was stirred at room temperature for 24 h. The reaction was quenched by addition of H_2_O, and the solution was adjusted to pH 6–7 by addition of NaOH_(aq)_. The orange precipitate was collected by vacuum filtration. The crude product was recrystallized from MeOH/Et_2_O to give pure compound **8** (1.3 g, 98% yield). C_15_H_14_N_2_OS; orange solid; mp 227.0–228.0 °C; TLC (EtOAc/hexane = 1:2) *R_f_* = 0.33; IR ν_max_ (neat) 3359, 3189, 2920, 2850, 1659, 1634, 1470, 1410, 1133, 734 cm^–1^; ^1^H NMR (400 MHz, DMSO-*d*_6_) δ 9.72 (s, 1 H), 7.79 (d, *J* = 8.9 Hz, 2 H), 7.72 (d, *J* = 8.8 Hz, 1 H), 7.33 (d, *J* = 2.4 Hz, 1 H), 6.93 (dd, *J* = 8.8, 2.4 Hz, 1 H), 6.79 (d, *J* = 8.9 Hz, 2 H), 2.99 (s, 6 H). ^13^C NMR (100 MHz, DMSO-*d*_6_) δ 164.2, 155.0, 151.7, 147.4, 135.2 (2 ×), 127.9, 122.4, 120.6, 115.5, 111.9 (2 ×), 106.7, 39.5 (2 ×). ESI-HRMS calcd for C_15_H_15_N_2_OS: 271.0905, found: *m/z* 271.0904 [M + H]^+^.

**2-(2-Azidoethoxy)ethyl methanesulfonate (S1a)^[S4]^**

A mixture of 2-(2-chloroethoxy)ethanol (1.24 g, 10 mmol) and NaN_3_ (1.65 g, 25 mmol) in H_2_O (10 mL) was stirred at 80 °C for 21 h. The reaction was quenched by addition of 5% NaOH_(aq)_, and then extracted with Et_2_O. The combined organic phase was dried over MgSO_4_, filtered, and concentrated under reduced pressure to give 2-(2-azidoethoxy)ethanol (1.08 g, 83% yield).

The above-prepared compound (1.08 g, 8.3 mmol) and Et_3_N (3.5 mL, 25 mmol) were dissolved in anhydrous CH_2_Cl_2_ (17 mL). Methanesulfonyl chloride (1.3 mL, 17 mmol) was added dropwise at 0 °C under an atmosphere of argon. The mixture was stirred at room temperature for 2 h. The reaction was quenched by addition of H_2_O. The organic phase was collected, and the aqueous phase was extracted with EtOAc. The combined organic phase was dried over MgSO_4_, filtered, concentrated under reduced pressure, and purified by flash chromatography on a silica gel column with elution of EtOAc/hexane (1:1) to give the mesylated compound **S1a** (1.7 g, 98% yield).

2-(2-Azidoethoxy)ethanol: C_4_H_9_N_3_O_2_; colorless liquid; TLC (CH_2_Cl_2_/MeOH = 9:1) *R_f_* = 0.5; IR ν_max_ (neat) 3396, 2930, 2872, 2110, 1443, 1286, 1127, 1066, 922, 888 cm^–1^; ^1^H NMR (400 MHz, CDCl_3_) δ 3.72 (t, *J* = 4.5 Hz, 2 H), 3.67 (t, *J* = 5.0 Hz, 2 H), 3.58 (t, *J* = 4.5 Hz, 2 H), 3.38 (t, *J* = 5.0 Hz, 2 H), 2.16–2.09 (m, 1 H); ^13^C NMR (100 MHz, CDCl_3_) δ 72.4, 70.0, 61.7, 50.7.

Compound **S1a**: C_5_H_11_N_3_O_4_S yellow liquid; TLC (EtOAc/hexane = 1:1) *R_f_* = 0.43; IR ν_max_ (neat) 2939, 2113, 1350, 1174, 1134, 1018, 973, 922, 799 cm^–1^; ^1^H NMR (400 MHz, CDCl_3_) δ 4.35 (t, *J* = 4.4 Hz, 2 H), 3.76 (t, *J* = 4.9 Hz, 2 H), 3.67 (t, *J* = 4.4 Hz, 2 H), 3.38 (t, *J* = 4.9 Hz, 2 H), 3.04 (s, 3 H); ^13^C NMR (100 MHz, CDCl_3_) δ 70.2, 68.9, 68.7, 50.6, 37.5. ESI-HRMS calcd for C_5_H_11_N_3_NaO_4_S: 232.0362, found: *m/z* 232.0367 [M + Na]^+^.

**2-(2-(2-(2-Azidoethoxy)ethoxy)ethoxy)ethyl methanesulfonate (S1b)^[S5]^**

To a solution of tetraethylene glycol (3 g, 15 mmol) and Et_3_N (6.4 mL, 46 mmol) in anhydrous CH_2_Cl_2_ (30 mL) was added methanesulfonyl chloride (3.5 mL, 45 mmol) dropwise at 0 °C under an atmosphere of argon. The mixture was stirred at room temperature for 18 h. The reaction was quenched by addition of H_2_O. The organic phase was collected, and the aqueous phase was extracted with EtOAc. The combined organic phase was dried over MgSO_4_, filtered and concentrated under reduced pressure to give the dimesylated compound (5.1 g, 97% yield).

A mixture of the above-prepared dimesylated compound (350 mg, 1 mmol) and NaN_3_ (97.5 mg, 1.5 mmol) in CH_3_CN (10 mL) was heated under reflux for 16 h. The mixture was concentrated under reduced pressure, and extracted with EtOAc and H_2_O. The combined organic phase was dried over MgSO_4_, filtered, concentrated under reduced pressure, and then purified by flash chromatography on a silica gel column with elution of EtOAc/hexane (2:1) to give the azido compound **S1b** (131 mg, 44% yield).

Dimesylated compound: C_10_H_22_O_9_S_2_; red liquid; TLC (EtOAc) *R_f_* = 0.38; IR ν_max_ (neat) 3031, 2919, 1645, 1349, 1173, 1136, 1015, 921, 805 cm^–1^; ^1^H NMR (400 MHz, CDCl_3_) δ 4.37– 4.33 (m, 4 H), 3.76–3.72 (m, 4 H), 3.65–3.61 (m, 8 H), 3.05 (s, 6 H); ^13^C NMR (100 MHz, CDCl_3_) δ 70.6 (2 ×), 70.5 (2 ×), 69.2 (2 ×), 69.0 (2 ×), 37.6 (2 ×). ESI-HRMS calcd for C_10_H_22_NaO_9_S_2_: 373.0597, found: *m/z* 373.0592 [M + Na]^+^.

Compound **S1b**: C_9_H_19_N_3_O_6_S; yellow liquid; TLC (EtOAc/hexane = 2:1) *R_f_* = 0.43; IR ν_max_ (neat) 2919, 2858, 2109, 1699, 1349, 1192, 1174, 1120, 928, 785 cm^–1^; ^1^H NMR (400 MHz, CDCl_3_) δ 4.32 (t, *J* = 4.5 Hz, 2 H), 3.71 (t, *J* = 4.5 Hz, 2 H), 3.64–3.57 (m, 10 H), 3.33 (t, *J* = 5.0 Hz, 2 H), 3.01 (s, 3 H); ^13^C NMR (100 MHz, CDCl_3_) δ 70.5 (2 ×), 70.4 (2 ×), 69.9, 69.2, 68.8, 50.5, 37.5. ESI-HRMS calcd for C_9_H_19_N_3_NaO_6_S: 320.0887, found: *m/z* 320.0883 [M + Na]^+^

**4-(6-(2-(2-Azidoethoxy)ethoxy)benzo[*d*]thiazol-2-yl)-*N*,*N*-dimethylaniline (S2a)**

To a solution of compound **8** (100 mg, 0.37 mmol) in anhydrous DMF (3 mL) was added K_2_CO_3_ (102 mg, 0.74 mmol). The mixture was stirred for 30 min at room temperature, and compound **S1a** (116 mg, 0.56 mmol) was added. The mixture was stirred at 80 °C for 21 h, cooled, and concentrated under reduced pressure. The mixture was extracted with EtOAc and H_2_O. The organic phase was dried over MgSO_4_, filtered, concentrated under reduced pressure, and purified by flash chromatography on a silica gel column with elution of CH_2_Cl_2_ to give compound **S2a** (66 mg, 51% yield). C_19_H_21_N_5_O_2_S; yellow solid; mp 96.0–97.0 °C; TLC (CH_2_Cl_2_) *R_f_* = 0.53; IR ν_max_ (neat) 2920, 2849, 2096, 1608, 1451, 1286, 1263, 1225, 1069, 943, 819 cm^–1^; ^1^H NMR (400 MHz, CDCl_3_) δ 7.86 (dd, *J* = 8.9 Hz, 8.7 Hz, 3 H), 7.28 (s, 1 H), 7.03 (dd, *J* = 8.9, 2.2 Hz, 1 H), 6.68 (d, *J* = 8.7 Hz, 2 H), 4.13 (t, *J* = 4.5 Hz, 2 H), 3.83 (t, *J* = 4.5 Hz, 2 H), 3.70 (t, *J* = 5.0 Hz, 2 H), 3.37 (t, *J* = 5.0 Hz, 2 H), 2.98 (s, 6 H); ^13^C NMR (100 MHz, CDCl_3_) δ 166.5, 156.1, 151.8, 148.8, 135.5, 128.4 (2 ×), 122.5, 121.3, 115.4, 111.6 (2 ×), 105.4, 70.1, 69.6, 68.0, 50.6, 40.0 (2 ×). ESI-HRMS calcd for C_19_H_22_N_5_O_2_S: 384.1489, found: *m/z* 384.1473 [M + H]^+^.

**4-(6-(2-(2-(2-(2-Azidoethoxy)ethoxy)ethoxy)ethoxy)benzo[*d*]thiazol-2-yl)-*N*,*N*-dimethylaniline (S2b)**

To a solution of compound **8** (242 mg, 0.9 mmol) in anhydrous DMF (8 mL) was added K_2_CO_3_ (248 mg, 1.8 mmol). The mixture was stirred for 30 min at room temperature, and compound **S1b** (400 mg, 1.35 mmol) was added. The mixture was stirred at 80 °C for 24 h, cooled, and concentrated under reduced pressure. The mixture was extracted with CH_2_Cl_2_ and H_2_O. The organic phase was dried over MgSO_4_, filtered, concentrated under reduced pressure, and purified by flash chromatography on a silica gel column with elution of EtOAc/CH_2_Cl_2_ (1:9) to give compound **S2b** (249 mg, 59% yield). C_23_H_29_N_5_O_4_S; yellow solid; mp 48.0–49.5°C; TLC (EtOAc/CH_2_Cl_2_ = 1:9) *R_f_* = 0.38; IR ν_max_ (neat) 3357, 3190, 2919, 2849, 2090, 1659, 1634, 1470, 1190, 1055 cm^–1^; ^1^H NMR (400 MHz, CDCl_3_) δ 7.87 (d, *J* = 8.9 Hz, 2 H), 7.83 (d, *J* = 8.9 Hz, 1 H), 7.29 (d, *J* = 2.4 Hz, 1 H), 7.02 (dd, *J* = 8.9, 2.4 Hz, 1 H), 6.69 (d, *J* = 8.9 Hz, 2 H), 4.15 (t, *J* = 4.8 Hz, 2 H), 3.85 (t, *J* = 4.8 Hz, 2 H), 3.72–3.69 (m, 2 H), 3.68–3.65 (m, 2 H), 3.64–3.61 (m, 6 H), 3.33 (t, *J* = 5.1 Hz, 2 H), 2.99 (s, 6 H); ^13^C NMR (100 MHz, CDCl_3_) δ 166.5, 156.2, 151.9, 148.7, 135.5, 128.5 (2 ×), 122.5, 121.3, 115.4, 111.7 (2 ×), 105.4, 70.8, 70.6 (3 ×), 69.9, 69.6, 68.1, 50.6, 40.2 (2 ×). ESI-HRMS calcd for C_23_H_30_N_5_O_4_S: 472.2013, found: *m/z* 472.1992 [M + H]^+^

**2,2,3,3-Tetramethyl-4,7,10-trioxa-3-siladodecan-12-yl methanesulfonate (S3a)^[S6]^**

To a solution of triethylene glycol (1 g, 6.65 mmol), imidazole (1.36 g, 19.95 mmol) and 4-dimethylaminopyridine (DMAP) (81 mg, 0.07 mmol) in anhydrous CH_2_Cl_2_/DMF (27 mL/ 3mL) was added *tert*-butyldimethylsilyl chloride (1 g, 6.65 mmol) dropwise at 0 °C. The mixture was stirred at room temperature for 11 h. The reaction was quenched by addition of H_2_O, and the solution was adjusted to pH 4–6 by addition of 1 M HCl_(aq)_. The organic phase was collected, and the aqueous phase was extracted with EtOAc. The combined organic phase was dried over MgSO_4_, filtered, concentrated under reduced pressure, and purified by flash chromatography on a silica gel column with elution of CH_2_Cl_2_/MeOH (25:1) to give mono-silylated compound (1.13 g, 64% yield).

To a solution of the above-prepared mono-silylated compound (2.4 g, 9 mmol) and Et_3_N (3.8 mL, 27 mmol) in anhydrous CH_2_Cl_2_ (50 mL) was added methanesulfonyl chloride (1.1 mL, 14 mmol) dropwise at 0 °C under an atmosphere of argon. The mixture was stirred at room temperature for 2 h. The reaction was quenched by addition of H_2_O. The organic phase was collected, and the aqueous phase was extracted with EtOAc. The combined organic phase was dried over MgSO_4_, filtered, concentrated under reduced pressure, and purified by flash chromatography on a silica gel column with elution of EtOAc/hexane (2:3) to give mesylated compound **S3a** (2.36 g, 76% yield).

Triethylene glycol mono-silylated compound: C_12_H_28_O_4_Si; colorless liquid; TLC (CH_2_Cl_2_/MeOH = 5:1) *R_f_* = 0.5; IR ν_max_ (neat) 3419, 2929, 2858, 1472, 1255, 1106, 836, 777 cm^–1^; ^1^H NMR (400 MHz, CDCl_3_) δ 3.75 (t, *J* = 5.3 Hz, 2 H), 3.71 (t, *J* = 4.5 Hz, 2 H), 3.65 (s, 4 H), 3.58 (t, *J* = 4.5 Hz, 2 H), 3.55 (t, *J* = 5.3 Hz, 2 H), 1.90 (br, 1 H), 0.87 (s, 9 H), 0.05 (s, 6 H). ^13^C NMR (100 MHz, CDCl_3_) δ 72.7, 72.5, 70.8, 70.5, 62.7, 61.8, 25.9 (3 ×), 18.4, –5.3 (2 ×). ESI-HRMS calcd for C_12_H_28_NaO_4_Si: 287.1649, found: *m/z* 287.1656 [M + Na]^+^.

Compound **S3a**: C_13_H_30_O_6_SSi; yellow liquid; TLC (EtOAc/hexane = 1:2) *R_f_* = 0.5; IR ν_max_ (neat) 3359, 3186, 2919, 2848, 1646, 1348, 1173, 923 cm^–1^; ^1^H NMR (400 MHz, CDCl_3_) δ 4.38–4.31 (m, 2 H), 3.78–3.68 (m, 4 H), 3.63 (s, 4 H), 3.52 (t, *J* = 5.3 Hz, 2 H), 3.04 (s, 3 H), 0.86 (s, 9 H), 0.04 (s, 6 H); ^13^C NMR (100 MHz, CDCl_3_) δ 72.7, 70.7 (2 ×), 69.2, 69.0, 62.7, 37.7, 25.9 (3 ×), 18.3, –5.3 (2 ×). ESI-HRMS calcd for C_13_H_30_NaO_6_SSi: 365.1425, found: *m/z* 365.1430 [M + Na]^+^.

**2,2,3,3-Tetramethyl-4,7,10,13,16-pentaoxa-3-silaoctadecan-18-yl methanesulfonate (S3b)^[S6]^**

To a solution of pentaethylene glycol (3 g, 13 mmol), imidazole (2.6 g, 38 mmol) and 4-dimethylaminopyridine (DMAP) (152 mg, 1.25 mmol) in anhydrous CH_2_Cl_2_/DMF (80 mL/ 5 mL) was added t*ert*-butyldimethylsilyl chloride (2.3 g, 15 mmol) dropwise at 0 °C. The mixture was stirred at room temperature for 17 h. The reaction was quenched by addition of H_2_O, and the pH was adjusted to 4–6 by addition of 1 M HCl_(aq)_. The organic phase was collected, and the aqueous phase was extracted with EtOAc. The combined organic phase was dried over MgSO_4_, filtered, concentrated under reduced pressure, and then purified by flash chromatography on a silica gel column with elution of EtOAc/hexane (4:1) to give mono-silylated compound (3.1 g, 70% yield).

To a solution of the above-prepared mono-silylated compound (4.2 g, 12 mmol) and Et_3_N (5 mL, 37 mmol) in anhydrous CH_2_Cl_2_ (30 mL) was added methanesulfonyl chloride (1.8 mL, 24 mmol) dropwise at 0 °C under an atmosphere of argon. The mixture was stirred at room temperature for 2 h. The reaction was quenched by addition of H_2_O. The organic phase was collected, and the aqueous phase was extracted with EtOAc. The combined organic phase was dried over MgSO_4_, filtered, concentrated under reduced pressure, and purified by flash chromatography on a silica gel column with elution of EtOAc/hexane (5:2) to give the mesylated compound **S3b** (4.9 g, 93% yield).

Pentaethylene glycol mono-silylated compound: C_16_H_36_O_6_Si; colorless liquid; TLC (EtOAc/hexane = 4:1) *R_f_* = 0.38; IR ν_max_ (neat) 3422, 2918, 1643, 1458, 1354, 1093, 947 cm^–1^; ^1^H NMR (400 MHz, CDCl_3_) δ 3.74 (t, *J* = 5.4 Hz, 2 H), 3.70 (t, *J* = 4.5 Hz, 2 H), 3.66–3.61 (m, 12 H), 3.56 (t, *J* = 4.5 Hz, 2 H), 3.53 (t, *J* = 5.4 Hz, 2 H), 2.02 (br, 1 H), 0.87 (s, 9 H), 0.04 (s, 6 H); ^13^C NMR (100 MHz, CDCl_3_) δ 72.7, 72.5, 70.7, 70.6 (4 ×), 70.3, 62.7, 61.8, 25.9 (3 ×), 18.4, –5.3 (2 ×). ESI-HRMS calcd for C_16_H_37_O_6_Si: 353.2354, found: *m/z* 353.2354 [M + H]^+^.

Compound **S3b**: C_17_H_38_O_8_SSi; yellow liquid; TLC (EtOAc/hexane = 5:2) *R_f_* = 0.5; IR ν_max_ (neat) 3397, 2876, 1723, 1646, 1456, 1350, 1174, 1099, 923 cm^–1^; ^1^H NMR (400 MHz, CDCl_3_) δ 4.29 (t, *J* = 4.5 Hz, 2 H), 3.71–3.63 (m, 4 H), 3.62–3.50 (m, 12 H), 3.46 (t, *J* = 5.4, 2 H), 2.99 (s, 3 H), 0.80 (s, 9 H), -0.03 (s, 6 H); ^13^C NMR (100 MHz, CDCl_3_) δ 72.4, 70.5, 70.4 (4 ×), 70.3, 69.2, 68.8, 62.5, 37.5, 25.7 (3 ×), 18.1, –5.5 (2 ×). ESI-HRMS calcd for C_17_H_39_O_8_SSi: 431.2129, found: *m/z* 431.2106 [M + H]^+^.

***N,N*-Dimethyl-4-(6-((2,2,3,3-tetramethyl-4,7,10-trioxa-3-siladodecan-12-yl)oxy)benzo[*d*]thiazol-2-yl)aniline (S4a)**

To a solution of compound **8** (250 mg, 0.92 mmol) in anhydrous DMF (4.5 mL) was added K_2_CO_3_ (254 mg, 1.84 mmol). The mixture was stirred for 30 min at room temperature, and the mesylated compound **S3a** (800 mg, 2.50 mmol) was added. The mixture was stirred at 90 °C for 24 h, cooled, and concentrated under reduced pressure. The mixture was extracted with EtOAc and H_2_O. The organic phase was dried over MgSO_4_, filtered, concentrated under reduced pressure, and purified by flash chromatography on a silica gel column with elution of EtOAc/hexane (1:3) to give compound **S4a** (319 mg, 67% yield). C_27_H_40_N_2_O_4_SSi; yellow solid; mp 92.0–93.5 °C; TLC (EtOAc/hexane = 1:2) *R_f_* = 0.35; IR ν_max_ (neat) 2925, 2855, 1608, 1493, 1450.8, 1226, 1071,836, 819 cm^–1^; ^1^H NMR (400 MHz, CDCl_3_) δ 7.86 (d, *J* = 8.9 Hz, 2 H), 7.83 (d, *J* = 8.9 Hz, 1 H), 7.30 (d, *J* = 2.5 Hz, 1 H), 7.03 (dd, *J* = 8.9, 2.5 Hz, 1 H), 6.71 (d, *J* = 8.9 Hz, 2 H), 4.16 (t, *J* = 4.8 Hz, 2 H), 3.87 (t, *J* = 4.8 Hz, 2 H), 3.75 (t, *J* = 5.4 Hz, 2 H),3.79–3.60 (m, 4 H), 3.55 (t, *J* = 5.4 Hz, 2 H), 3.01 (s, 6 H), 0.87 (s, 9 H), 0.04 (s, 6 H); ^13^C NMR (100 MHz, CDCl_3_) δ 166.6, 156.2, 151.9, 149.0, 135.7, 128.5 (2 ×), 122.7, 121.6, 115.4, 111.7 (2 ×), 105.4, 72.7, 70.9, 70.8, 69.7, 68.1, 62.7, 40.1 (2 ×), 25.9 (3 ×), 18.3, –5.3 (2 ×). ESI-HRMS calcd for C_27_H_41_N_2_O_4_SSi: 517.2551, found: *m/z* 517.2564 [M + H]^+^.

***N*,*N*-Dimethyl-4-(6-((2,2,3,3-tetramethyl-4,7,10,13,16-pentaoxa-3-silaoctadecan-18-yl)oxy)benzo[*d*]thiazol-2-yl)aniline (S4b)**

To a solution of compound **8** (50 mg, 0.19 mmol) in anhydrous DMF (4 mL) was added K_2_CO_3_ (38 mg, 0.28 mmol). The mixture was stirred for 30 min at room temperature, and compound **S3b** (96 mg, 0.22 mmol) was added. The mixture was stirred at 80 °C for 21 h, cooled, and concentrated under reduced pressure. The mixture was extracted with EtOAc and H_2_O. The organic phase was dried over MgSO_4_, filtered, concentrated under reduced pressure, and purified by flash chromatography on a silica gel column with elution of EtOAc/hexane (2:3) to give compound **S4b** (62 mg, 59% yield). C_31_H_48_N_2_O_6_SSi; yellow liquid; TLC (EtOAc/hexane = 2:3) *R_f_* = 0.25; IR ν_max_ (neat) 2920, 2850, 1668, 1606, 1456, 1114, 1069, 820 cm^–1^; ^1^H NMR (400 MHz, CDCl_3_) δ 7.88 (d, *J* = 8.8 Hz, 2 H), 7.82 (d, *J* = 8.9 Hz, 1 H), 7.29 (d, *J* = 2.1 Hz, 1 H), 7.02 (dd, *J* = 8.9, 2.1 Hz, 1 H), 6.70 (d, *J* = 8.8 Hz, 2 H), 4.15 (t, *J* = 4.5 Hz, 2 H), 3.85 (t, *J* = 4.5 Hz, 2 H), 3.72 (q, *J* = 5.4 Hz, 4 H), 3.65–3.59 (m, 10 H), 3.52 (t, *J* = 5.4 Hz, 2 H), 3.00 (s, 6 H), 0.86 (s, 9 H), 0.03 (s, 6 H); ^13^C NMR (100 MHz, CDCl_3_) δ 166.6, 156.3, 151.9, 148.7, 135.5, 128.5 (2 ×), 122.6, 121.4, 115.5, 111.7 (2 ×), 105.4, 72.6, 70.8, 70.7, 70.6 (4 ×), 69.7, 68.1, 62.7, 40.2 (2 ×), 25.9 (3 ×), 18.3, –5.3 (2 ×). ESI-HRMS calcd for C_31_H_49_N_2_O_6_SSi: 605.3075, found: *m/z* 605.3080 [M + H]^+^.

**2-(2-(2-((2-(4-(Dimethylamino)phenyl)benzo[*d*]thiazol-6-yl)oxy)ethoxy)ethoxy) ethanol (S5a, JMF4565)**

A mixture of silylated compound **S4a** (250 mg, 0.48 mmol) and tetra-*n*-butylammonium fluoride (0.72 mL of 1M THF solution, 0.72 mmol) was stirred at room temperature for 6 h. The mixture was concentrated under reduced pressure, and extracted with CH_2_Cl_2_ and H_2_O. The combined organic phase was dried over MgSO_4_, filtered, concentrated under reduced pressure, and purified by flash chromatography on a silica gel column with elution of EtOAc to give alcohol compound **S5a** (193 mg, 99% yield). The purity of compound **S5a** was 99.8% as shown by HPLC on a silica column (Dikma, 10 × 250 mm, 10 μm particle size), elution: EtOAc/MeOH = 97:3 at a flow rate of 3.0 mL/min, *t*_R_ = 10.8 min. C_21_H_26_N_2_O_4_S; white solid; mp 99.0–100 °C; TLC (EtOAc) *R_f_* = 0.45; IR ν_max_ (neat) 3359, 3190, 2920, 2849, 1646, 1607, 1109, 1055, 823 cm^–1^; ^1^H NMR (400 MHz, CDCl_3_) δ 7.87 (d, *J* = 8.8 Hz, 2 H), 7.82 (d, *J* = 8.9 Hz, 1 H), 7.29 (d, *J* = 2.5 Hz, 1 H), 7.02 (dd, *J* = 8.9, 2.5 Hz, 1 H), 6.69 (d, *J* = 8.8 Hz, 2 H), 4.14 (t, *J* = 4.6 Hz, 2 H), 3.84 (t, *J* = 4.6 Hz, 2 H), 3.74–3.63 (m, 6 H), 3.58 (t, *J* = 4.6 Hz, 2 H), 2.99 (s, 6 H), 2.7 (br, 1 H); ^13^C NMR (100 MHz, CDCl_3_) δ 166.6, 156.1, 151.9, 149.0, 135.6, 128.4 (2 ×), 122.6, 121.5, 115.3, 111.7 (2 ×), 105.4, 72.5, 70.8, 70.3, 69.7, 68.0, 61.7, 40.1 (2 ×). ESI-HRMS calcd for C_21_H_27_N_2_O_4_S: 403.1686, found: *m/z* 403.1690 [M + H]^+^.

**14-((2-(4-(Dimethylamino)phenyl)benzo[*d*]thiazol-6-yl)oxy)-3,6,9,12-tetraoxatetradecan-1-ol (S5b)**

A mixture of silylated compound **S4b** (464 mg, 0.77 mmol) and tetra-*n*-butylammonium fluoride (1.25 mL of 1M THF solution, 1.25 mmol) was stirred at room temperature for 4 h. The mixture was concentrated under reduced pressure, and extracted with CH_2_Cl_2_ and H_2_O. The combined organic phase was dried over MgSO_4_, filtered, concentrated under reduced pressure, and purified by flash chromatography on a silica gel column with elution of CH_2_Cl_2_/MeOH (30:1) to give alcohol compound **S5b** (302 mg, 80% yield). C_25_H_34_N_2_O_6_S; yellow solid; mp 77.5–78.5°C; TLC (CH_2_Cl_2_/MeOH = 30:1) *R_f_* = 0.25; IR ν_max_ (neat) 3389, 3342, 3181, 2919, 2849, 1646, 1607, 1470, 1447, 1117, 1067, 816 cm^–1^; ^1^H NMR (400 MHz, CDCl_3_) δ 7.87 (d, *J* = 8.9 Hz, 2 H), 7.83 (d, *J* = 8.9 Hz, 1 H), 7.31 (d, *J* = 2.5 Hz, 1 H), 7.03 (dd, *J* = 8.9, 2.5 Hz, 1 H), 6.71 (d, *J* = 8.9 Hz, 2 H), 4.17 (t, *J* = 4.8 Hz, 2 H), 3.86 (t, *J* = 4.8 Hz, 2 H), 3.73–3.66 (m, 6 H), 3.62 (d, *J* = 5.0 Hz, 8 H), 3.56 (t, *J* = 4.5 Hz, 2 H), 3.01 (s, 6 H), 2.69 (br, 1 H); ^13^C NMR (100 MHz, CDCl_3_) δ 166.6, 156.2, 151.9, 148.9, 135.6, 128.5 (2 ×), 122.6, 121.5, 115.5, 111.7 (2 ×), 105.4, 72.5, 70.8, 70.6 (4 ×), 70.3, 69.7, 68.1, 61.7, 40.1 (2 ×). ESI-HRMS calcd for C_25_H_35_N_2_O_6_S: 491.2210, found: *m/z* 491.2218 [M + H]^+^.

**2-(2-(2-((2-(4-(Dimethylamino)phenyl)benzo[*d*]thiazol-6-yl)oxy)ethoxy)ethoxy)ethyl 4-methylbenzenesulfonate (S6a)**

To a solution of alcohol compound **S5a** (50 mg, 0.12 mmol) and 4-toluenesulfonyl chloride (47 mg, 0.25 mmol) in CH_2_Cl_2_ (1.5 mL) was added KOH (28 mg, 0.50 mmol) at 0 °C. The mixture was stirred at room temperature for 4 h. The reaction was quenched by addition of H_2_O, and then extracted with CH_2_Cl_2_. The combined organic phase was dried over MgSO_4_, filtered, concentrated under reduced pressure, and purified by flash chromatography on a silica gel column with elution of EtOAc/hexane (2:1) to give the tosylated compound **S6a** (60 mg, 87% yield). C_28_H_32_N_2_O_6_S_2_; yellow solid; mp 113.5–115.0 °C; TLC (EtOAc/CH_2_Cl_2_ = 1:5) *R_f_* = 0.45; IR ν_max_ (neat) 3381, 3166, 2920, 2849, 1647, 1632, 1606, 1189, 1175, 555.7 cm^–1^; ^1^H NMR (400 MHz, CDCl_3_) δ 7.87 (d, *J* = 8.8 Hz, 2 H), 7.82 (d, *J* = 8.9 Hz, 1 H), 7.76 (d, *J* = 8.1 Hz, 2 H), 7.33–7.23 (m, 3 H), 7.01 (dd, *J* = 8.9, 2.6 Hz, 1 H), 6.69 (d, *J* = 8.8 Hz, 2 H), 4.19–4.09 (m, 4 H), 3.81 (t, *J* = 4.7 Hz, 2 H), 3.75–3.61 (m, 4 H), 3.61–3.55 (m, 2 H), 3.00 (s, 6 H), 2.37 (s, 3 H); ^13^C NMR (100 MHz, CDCl_3_) δ 166.5, 156.1, 151.8, 149.0, 144.7, 135.6, 132.8, 129.7 (2 ×), 128.4 (2 ×), 127.8 (2 ×), 122.6, 121.4, 115.3, 111.6 (2 ×), 105.3, 70.7 (2 ×), 69.7, 69.2, 68.6, 68.0, 40.1 (2 ×), 21.5. ESI-HRMS calcd for C_28_H_33_N_2_O_6_S_2_: 557.1775, found: *m/z* 557.1790 [M + H]^+^.

**14-((2-(4-(Dimethylamino)phenyl)benzo[*d*]thiazol-6-yl)oxy)-3,6,9,12-tetraoxatetradecyl 4-methylbenzenesulfonate (S6b)**

To a solution of compound **S5b** (700 mg, 1.4 mmol) and 4-toluenesulfonyl chloride (543 mg, 2.8 mmol) in CH_2_Cl_2_ (1 mL) was added KOH (320 mg, 5.7 mmol) at 0 °C. The mixture was stirred at room temperature for 14 h. The reaction was quenched by addition of H_2_O, and then extracted with CH_2_Cl_2_. The combined organic phase was dried over MgSO_4_, filtered, concentrated under reduced pressure, and purified by flash chromatography on a silica gel column with elution of EtOAc/CH_2_Cl_2_ (1:4) to give the tosylated compound **S6b** (881 mg, 96% yield). C_32_H_40_N_2_O_8_S_2_; yellow solid; mp 54.0–55.5°C; TLC (EtOAc/DCM = 1:4) *R_f_* = 0.43; IR ν_max_ (neat) 3362, 3189, 2920, 2849, 1606, 1456, 1189, 1176, 1122, 817 cm^–1^; ^1^H NMR (400 MHz, CDCl_3_) δ 7.86 (d, *J* = 8.7 Hz, 2 H), 7.81 (d, *J* = 8.9 Hz, 1 H), 7.74 (d, *J* = 8.0 Hz, 2 H), 7.3–7.25 (m, 3 H), 7.01 (dd, *J* = 8.9, 2.2 Hz, 1 H), 6.68 (d, *J* = 8.7 Hz, 2 H), 4.14 (t, *J* = 4.8 Hz, 2 H), 4.10 (t, *J* = 4.8 Hz, 2 H), 3.83 (t, *J* = 4.8 Hz, 2 H), 3.69 (t, *J* = 4.5 Hz, 2 H), 3.66– 3.55 (m, 8 H), 3.53 (s, 4 H), 2.99 (s, 6 H), 2.37 (s, 3 H); ^13^C NMR (100 MHz, CDCl_3_) δ 166.4, 156.2, 151.8, 148.8, 144.6, 135.5, 132.9, 129.7 (2 ×), 128.4 (2 ×), 127.8 (2 ×), 122.5, 121.3, 115.4, 111.6 (2 ×), 105.3, 70.7, 70.6, 70.5 (3 ×), 70.4, 69.6, 69.1, 68.5, 68.0, 40.0 (2 ×), 21.5. ESI-HRMS calcd for C_32_H_41_N_2_O_8_S_2_: 645.2299, found: *m/z* 645.2308 [M + H]^+^.

**2,2,3,3-Tetramethyl-4,7,10-trioxa-3-siladodecan-12-amine (S7)**

A mixture of mesylated compound **S3a** (200 mg, 0.58 mmol) and NaN_3_ (76 mg, 1.2 mmol) in EtOH (3 mL) was heated under reflux for 24 h. The mixture was concentrated under reduced pressure, and then extracted with EtOAc and H_2_O. The combined organic phase was dried over MgSO_4_, filtered, and concentrated under reduced pressure to afford an azido compound. Without further purification, the azido compound in THF (3 mL) was stirred with PPh_3_ (460 mg, 1.75 mmol) and H_2_O (31 µL, 1.75 mmol) at room temperature for 27 h. The mixture was concentrated under reduced pressure, and purified by flash chromatography on a silica gel column with elution of CH_2_Cl_2_/MeOH (20:1) to give amino compound **S7** (112 mg, 73% yield for 2 steps). C_12_H_29_NO_3_Si; colorless liquid; TLC (CH_2_Cl_2_/MeOH = 9:1) *R_f_* = 0.15; IR ν_max_ (neat) 3358, 3194, 2920, 2850, 1659, 1649, 1632, 1470, 1413, 1140 cm^–1^; ^1^H NMR (400 MHz, CDCl_3_) δ 3.73 (t, *J* = 5.3 Hz, 2 H), 3.64–3.56 (m, 4 H), 3.52 (t, *J* = 5.3 Hz, 2 H), 3.48 (t, *J* = 5.2 Hz, 2 H), 2.84 (s, 2 H), 2.02 (br, 2 H), 0.85 (s, 9 H), 0.03 (s, 6 H); ^13^C NMR (100 MHz, CDCl_3_) δ 73.1, 72.6, 70.6, 70.3, 62.7, 41.6, 25.8 (3 ×), 18.3, –5.3 (2 ×). ESI-HRMS calcd for C_12_H_30_NO_3_Si: 264.1989, found: *m/z* 264.1994 [M + H]^+^.

**2-(2,6-Dioxopiperidin-3-yl)-4-((2-(2-(2-hydroxyethoxy)ethoxy)ethyl)amino) isoindoline-1,3-dione (S8**, **JMF4576)**

A mixture of compound **10** (50 mg, 0.18 mmol), compound **S7** (53 mg, 0.2 mmol) and DIPEA (63 µL, 0.36 mmol) in NMP (1 mL) was stirred at 90 °C for 23 h. The mixture was extracted with EtOAc and H_2_O. The combined organic phase was dried over MgSO_4_, filtered, concentrated under reduced pressure and purified by flash chromatography on a silica gel column with elution of EtOAc/hexane (1:1) to give a POM-PEG mono-silylated compound (32 mg, 34% yield).

A solution of the above-prepared mono-silylated compound (60 mg, 0.12 mmol) and 1 M tetra-*n*-butylammonium fluoride in THF (0.35 mL, 0.35 mmol) was stirred at room temperature for 17 h. The mixture was concentrated under reduced pressure, and extracted with CH_2_Cl_2_ and H_2_O. The combined organic phase was dried over MgSO_4_, filtered, concentrated under reduced pressure, and then purified by flash chromatography on a silica gel column with elution of EtOAc to give compound **S8** (36 mg, 77% yield).

POM-PEG mono-silylated compound: C_25_H_37_N_3_O_7_Si; yellow solid; mp 84.0–85.0 °C; TLC (EtOAc/ CH_2_Cl_2_ = 1:1) *R_f_* = 0.33; IR ν_max_ (neat) 3357, 3198, 2920, 2850, 1696, 1654, 1636, 1632, 1470, 1410, 1362, 1267 1116 cm^–1^; ^1^H NMR (400 MHz, CDCl_3_) δ 8.33 (s, 1 H), 7.45 (dd, *J* = 8.5, 7.2 Hz, 1 H), 7.07 (d, *J* = 7.2 Hz, 1 H), 6.90 (d, *J* = 8.5 Hz, 1 H), 6.45 (t, *J* = 5.6 Hz, 1 H), 4.89 (q, *J* = 5.4 Hz, 1 H), 3.73 (t, *J* = 5.4 Hz, 2 H), 3.69 (t, *J* = 5.5, 2 H), 367–3.60 (m, 4 H), 3.53 (t, *J* = 5.4 Hz, 2 H), 3.44 (q, *J* = 5.5 Hz, 2 H), 2.89–2.65 (m, 3 H), 2.14–2.04 (m, 1 H), 0.86 (s, 9 H), 0.03 (s, 6 H); ^13^C NMR (100 MHz, CDCl_3_) δ 171.3, 169.2, 168.5, 167.5, 146.8, 135.9, 132.4, 116.7, 111.5, 110.2, 72.6, 70.7, 70.6, 69.5, 62.6, 48.8, 42.3, 31.3, 25.8 (3 ×), 22.7, 18.3, –5.3 (2 ×). ESI-HRMS calcd for C_25_H_37_N_3_NaO7Si: 542.2293, found: *m/z* 542.2296 [M + Na]^+^.

Compound **S8**: C_19_H_23_N_3_O_7_; orange solid; mp 71.5–72.0 °C; TLC (EtOAc) *R_f_* = 0.25; IR ν_max_ (neat) 3359, 3190, 2919, 2850, 1695, 1659, 1632, 1470, 1410, 1358 cm^–1^; ^1^H NMR (400 MHz, CDCl_3_) δ 8.59 (s, 1 H), 7.45 (dd, *J* = 8.5, 7.2 Hz, 1 H), 7.06 (d, *J* = 7.2 Hz, 1 H), 6.87 (d, *J* = 8.5 Hz, 1 H), 6.52 (s, 1 H), 4.89 (q, *J* = 5.9 Hz, 1 H), 3.72– 3.67 (m, 4 H), 3.65 (s, 4 H), 3.57 (t, *J* = 4.5 Hz, 2 H), 3.44 (q, *J* = 4.5 Hz, 2 H), 2.85–2.67 (m, 3 H), 2.10–2.05 (m, 1 H), 1.99 (br, 1 H); ^13^C NMR (100 MHz, CDCl_3_) δ 171.3, 169.4, 168.5, 167.6, 146.8, 136.0, 132.5, 116.7, 111.7, 110.3, 72.6, 70.6, 70.3, 69.2, 61.7, 48.9, 42.2, 31.4, 22.7. ESI-HRMS calcd for C_19_H_24_N_3_O_7_: 406.1609, found: *m/z* 406.1637 [M + H]^+^. The purity of **JMF4576** was 97.1% as shown by HPLC on a silica column (Dikma, 10 × 250 mm, 10 μm particle size), elution: EtOAc/MeOH = 97:3 at a flow rate of 3.0 mL/min, *t*_R_ = 12.8 min.

**4-(6-(2-(2-Aminoethoxy)ethoxy)benzo[*d*]thiazol-2-yl)-*N*,*N*-dimethylaniline (9a)**

A solution of azido compound **S2a** (202 mg, 0.53 mmol) in THF (3 mL) was stirred with PPh_3_ (415 mg, 1.6 mmol) and H_2_O (30 µL, 1.6 mmol) at room temperature for 24 h. The mixture was concentrated under reduced pressure, and purified by flash chromatography on a silica gel column with elution of CH_2_Cl_2_/MeOH (10:1) to give the amino compound **9a** (195 mg, 99% yield). C_19_H_23_N_3_O_2_S; yellow solid; mp 66.5–67.5°C; TLC (CH_2_Cl_2_/MeOH = 7:1) *R_f_* = 0.13; IR ν_max_ (neat) 3358, 3190, 2919, 2849, 1659, 1653, 1634, 1470, 1423, 1264, 828 cm^–1^; ^1^H NMR (400 MHz, CDCl_3_) δ 7.83 (d, *J* = 8.8 Hz, 2 H), 7.80 (d, *J* = 8.9 Hz, 1 H), 7.26 (d, *J* = 2.1 Hz, 1 H), 7.00 (dd, *J* = 8.9, 2.1 Hz, 1 H), 6.65 (d, *J* = 8.8 Hz, 2 H), 4.10 (t, *J* = 4.4 Hz, 2 H), 3.77 (t, *J* = 4.4 Hz, 2 H), 3.52 (t, *J* = 4.9 Hz, 2 H), 2.95 (s, 6 H), 2.83 (s, 2 H), 1.91 (br, 2 H); ^13^C NMR (100 MHz, CDCl_3_) δ 166.4, 156.0, 151.7, 148.9, 135.6, 128.3 (2 ×), 122.5, 121.3, 115.2, 111.5 (2 ×), 105.2, 73.3, 69.3, 67.9, 41.5, 39.9 (2 ×). ESI-HRMS calcd for C_19_H_24_N_3_O_2_S: 358.1584, found: *m/z* 358.1570 [M + H]^+^.

**4-(6-(2-(2-(2-Aminoethoxy)ethoxy)ethoxy)benzo[*d*]thiazol-2-yl)-*N,N*-dimethyl aniline (9b)**

A mixture of tosylated compound **S6a** (55 mg, 0.10 mmol) and NaN_3_ (13 mg, 0.20 mmol) in EtOH (2 mL) was heated under reflux for 16 h. The mixture was concentrated under reduced pressure, and extracted with CH_2_Cl_2_ and H_2_O. The combined organic phase was dried over MgSO_4_, filtered, and concentrated under reduced pressure to afford an azido compound. Without further purification, the azido compound in THF (2 mL) was stirred with PPh_3_ (39 mg, 0.15 mmol) and H_2_O (2.7 µL, 0.15 mmol) at room temperature for 14 h. The mixture was concentrated under reduced pressure, and purified by flash chromatography on a silica gel column with elution of CH_2_Cl_2_/MeOH (9:1) to give the amino compound **9b** (31 mg, 77% overall yield). C_21_H_27_N_3_O_3_S; yellow solid; mp 86.5–88.5 °C; TLC (CH_2_Cl_2_/MeOH = 9:1) *R_f_* = 0.13; IR ν_max_ (neat) 3358, 3181, 2920, 2850, 1659, 1632, 1470, 1447, 812, 628 cm^–1^; ^1^H NMR (400 MHz, CD_3_OD) δ 7.74 (dd, *J* = 8.8, 2.6 Hz, 3 H), 7.32 (d, *J* = 2.6 Hz, 1 H), 7.04 (dd, *J* = 8.8, 2.6 Hz, 1 H), 6.69 (d, *J* = 8.8 Hz, 2 H), 4.85 (br, 2 H), 4.11 (t, *J* = 4.2 Hz, 2 H), 3.82 (m, *J* = 4.2 Hz, 2 H), 3.77–3.54 (m, 6 H), 3.00–2.88 (m, 8 H); ^13^C NMR (100 MHz, CD_3_OD) δ 168.9, 157.9, 153.8, 149.7, 136.7, 129.4 (2 ×), 123.1, 121.9, 116.8, 112.9 (2 ×), 106.5, 71.7, 71.3, 70.8, 69.2, 69.1, 41.0, 40.3 (2 ×). ESI-HRMS calcd for C_21_H_28_N_3_O_3_S: 402.1846, found: *m/z* 402.1835 [M + H]^+^.

**4-(6-(2-(2-(2-(2-Aminoethoxy)ethoxy)ethoxy)ethoxy)benzo[*d*]thiazol-2-yl)-*N*,*N*-dimethylaniline (9c)**

A solution of azido compound **S2b** (377 mg, 0.8 mmol) in THF (13 mL) was stirred with PPh_3_ (405 mg, 1.6 mmol) and H_2_O (30 µL, 1.6 mmol) at room temperature for 27 h. The mixture was concentrated under reduced pressure, and purified by flash chromatography on a silica gel column with elution of CH_2_Cl_2_/MeOH (20:1) to give the amino compound **9c** (311 mg, 87% yield).

C_23_H_31_N_3_O_4_S; yellow solid; mp 66.5–67.5°C; TLC (CH_2_Cl_2_/MeOH = 5:1) *R_f_* = 0.25; IR ν_max_ (neat) 3401, 3354, 3197, 2920, 2848, 1646, 1634, 1469, 1126 cm^–1^; ^1^H NMR (400 MHz, CDCl_3_) δ 7.84 (d, *J* = 9.0 Hz, 2 H), 7.80 (d, *J* = 8.9 Hz, 1 H), 7.29 (d, *J* = 2.5 Hz, 1 H), 7.00 (dd, *J* = 8.9, 2.5 Hz, 1 H), 6.67 (d, *J* = 9.0 Hz, 2 H), 4.14 (t, *J* = 4.8 Hz, 2 H), 3.83 (t, *J* = 4.8 Hz, 2 H), 3.71–3.67 (m, 2 H), 3.65–3.59 (m, 4 H), 3.58–3.55 (m, 2 H), 3.47 (t, *J* = 5.1 Hz, 2 H), 2.98 (s, 6 H), 2.83 (s, 2 H), 2.48 (br, 2 H); ^13^C NMR (100 MHz, CDCl_3_) δ 166.2, 155.9, 151.5, 148.7, 135.4, 128.1 (2 ×), 122.3, 121.1, 115.1, 111.3 (2 ×), 105.0, 70.4, 70.2 (3 ×), 69.9, 69.4, 67.7, 39.8 (3 ×). ESI-HRMS calcd for C_23_H_32_N_3_O_4_S: 446.2108, found: *m/z* 446.2097 [M + H]^+^.

**14-((2-(4-(Dimethylamino)phenyl)benzo[*d*]thiazol-6-yl)oxy)-3,6,9,12-tetraoxa tetradecan-1-amine (9d)**

A mixture of tosylated compound **S6b** (881 mg, 1.4 mmol) and NaN_3_ (177 mg, 2.7 mmol) in EtOH (13 mL) was heated under reflux for 22 h. The mixture was concentrated under reduced pressure, and then extracted with CH_2_Cl_2_ and H_2_O. The combined organic phase was dried over MgSO_4_, filtered, and concentrated under reduced pressure to afford an azido compound. Without further purification, the azido compound in THF (13 mL) was stirred with PPh_3_ (1.1 g, 4.1 mmol) and H_2_O (75 µL, 4.1 mmol) at room temperature for 27 h. The mixture was concentrated under reduced pressure, and purified by flash chromatography on a silica gel column with elution of CH_2_Cl_2_/MeOH (15:1) to give the amino compound **9d** (612 mg, 92% overall yield). C_25_H_35_N_3_O_5_S; yellow solid; mp 82.0–83.5°C; TLC (CH_2_Cl_2_/MeOH = 9:1) *R_f_* = 0.15; IR ν_max_ (neat) 3362, 3201, 2919, 2849, 1659, 1634, 1470, 1275, 769, 764, 750 cm^–1^; ^1^H NMR (400 MHz, CDCl_3_) δ 7.86 (d, *J* = 8.9 Hz, 2 H), 7.81 (d, *J* = 8.9 Hz, 1 H), 7.31 (d, *J* = 2.5 Hz, 1 H), 7.02 (dd, *J* = 8.9, 2.5 Hz, 1 H), 6.70 (d, *J* = 8.9 Hz, 2 H), 4.16 (t, *J* = 4.8 Hz, 2 H), 3.85 (t, *J* = 4.8 Hz, 2 H), 3.73–3.68 (m, 2 H), 3.67–3.56 (m, 10 H), 3.47 (t, *J* = 5.2 Hz, 2 H), 3.00 (s, 6 H), 2.83 (s, 2 H), 2.18 (br, 2 H); ^13^C NMR (100 MHz, CDCl_3_) δ 166.6, 156.2, 151.9, 149.0, 135.7, 128.4 (2 ×), 122.6, 121.5, 115.4, 111.7 (2 ×), 105.4, 72.9, 70.8, 70.5 (4 ×), 70.2, 69.7, 68.1, 41.6, 40.0 (2 ×). ESI-HRMS calcd for C_25_H_36_N_3_O_5_S: 490.2370, found: *m/z* 490.2385 [M + H]^+^.

**2-(2,6-Dioxopiperidin-3-yl)-4-fluoroisoindoline-1,3-dione (10)^[S7]^**

A mixture of 3-fluorophthalic anhydride (100 mg, 0.6 mmol), 2,6-dioxopiperidin-3-amine hydrochloride (99 mg, 0.6 mmol) and NaOAc∙3H_2_O (98 mg, 0.72 mmol) in AcOH (3 mL) was heated under reflux for 12 h. The mixture was concentrated under reduced pressure, and purified by flash chromatography on a silica gel column with elution of CH_2_Cl_2_/MeOH (100:1) to give compound **10** (155 mg, 93% yield). The purity of compound **10** was 98.9% as shown by HPLC on a silica column (Dikma, 10 × 250 mm, 10 μm particle size), elution: EtOAc/hexane = 4:1 at a flow rate of 3.0 mL/min, *t*_R_ = 8.1 min. C_13_H_9_FN_2_O_4_; white solid; mp 255.5–257.0 °C; TLC (CH_2_Cl_2_/MeOH = 9:1) *R_f_* = 0.5; IR ν_max_ (neat) 3252, 3107, 2927, 2845, 1717, 1393, 1261, 1199, 746, 597 cm^-1^; ^1^H NMR (400 MHz, DMSO-*d*_6_) δ 11.14 (s, 1 H), 7.95 (td, *J* = 7.9, 4.4 Hz, 1 H), 7.79 (d, *J* = 7.3 Hz, 1 H), 7.73 (t, *J* = 8.9 Hz, 1 H), 5.16 (dd, *J* = 12.9, 5.4 Hz, 1 H), 2.89 (ddd, *J* = 17.0, 14.0, 5.4 Hz, 1 H), 2.66–2.44 (m, 2H), 2.12–2.01 (m, 1H); ^13^C NMR (100 MHz, DMSO-*d*_6_) δ 172.8, 169.8, 166.2, 164.0, 156.9 (d, *J*_C-F_ = 260.9 Hz), 138.1 (d, *J*_C-F_ = 8.1 Hz), 133.5, 123.1 (d, *J*_C-F_ = 19.2 Hz), 120.1, 117.1 (d, *J*_C-F_ = 12.1 Hz), 49.2, 31.0, 21.9; ^19^F NMR (376 MHz, DMSO-*d*_6_) δ –115.52 (t, *J* = 4.4 Hz); ESI-HRMS calcd for C_13_H_9_FN_2_NaO_4_: 299.0439, found: *m/z* 299.0430 [M + Na]^+^.

**4-((2-(2-((2-(4-(Dimethylamino)phenyl)benzo[*d*]thiazol-6-yl)oxy)ethoxy)ethyl)amino)-2- (2,6-dioxopiperidin-3-yl)isoindoline-1,3-dione (1)**

A mixture of compound **9a** (181 mg, 0.66 mmol), compound **10** (180 mg, 0.50 mmol) and diisopropylethylamine (DIPEA, 180 µL, 1.01 mmol) in 1-methyl-2-pyrrolidone (NMP) (2.5 mL) was stirred at 90 °C for 18 h. The mixture was extracted with EtOAc and H_2_O. The combined organic phase was dried over MgSO_4_, filtered, concentrated under reduced pressure, and purified by flash chromatography on a silica gel column with elution of EtOAc/CH2Cl2 (1:2) to give the desired compound **1** (100 mg, 32% yield). The purity of compound **1** was 96.4% as shown by HPLC on a silica column (Dikma, 10 × 250 mm, 10 μm particle size), elution: EtOAc/hexane = 3:1 at a flow rate of 3.0 mL/min, *t*_R_ = 12.2 min. C_32_H_31_N_5_O_6_S; yellow solid; mp 157.0–158.0 °C; TLC (EtOAc/ CH_2_Cl_2_ = 1:2) *R_f_* = 0.63; IR ν_max_ (neat) 3359, 3182, 2919, 2849, 1699, 1695, 1657, 1557, 1538, 1471 cm^–1^; ^1^H NMR (400 MHz, CDCl_3_) δ 8.15 (s, 1 H), 7.97–7.80 (m, 3 H), 7.44 (t, *J* = 7.8 Hz, 1 H), 7.30 (s, 1 H), 7.10–7.00 (m, 2 H), 6.90 (d, *J* = 8.5 Hz, 1 H), 6.74 (d, *J* = 8.5 Hz, 2 H), 6.50 (s, 1 H), 4.86 (q, *J* = 5.4 Hz, 1 H), 4.19 (t, *J* = 4.3 Hz, 2 H), 3.87 (t, *J* = 4.3, 2 H), 3.79 (t, *J* = 5.1 Hz, 2 H), 3.48 (q, *J* = 5.1 Hz, 2 H), 3.03 (s, 6 H), 2.86–2.67 (m, 3 H), 2.08–2.00 (m, 1 H); ^13^C NMR (125 MHz, DMSO-*d_6_*) δ 172.9, 170.2, 169.0, 167.4, 165.5, 156.0, 151.9, 148.3, 146.5, 136.3, 135.2, 132.1, 128.2 (2 ×), 122.4, 120.5, 117.5, 115.7, 112.0 (2 ×), 110.8, 109.3, 105.8, 69.0, 68.8, 67.8, 48.6, 41.7 (2 ×), 31.1, 22.2, 18.6. ESI-HRMS calcd for C_32_H_32_N_5_O_6_S: 614.2068, found: *m/z* 614.2031 [M + H]^+^.

**4-((2-(2-(2-((2-(4-(Dimethylamino)phenyl)benzo[*d*]thiazol-6-yl)oxy)ethoxy) ethoxy)ethyl)amino)-2-(2,6-dioxopiperidin-3-yl)isoindoline-1,3-dione (2)**

By a procedure similar to that for compound **1**, the substitution reaction of **10** (74 mg, 0.26 mmol) with **9b** (54 mg, 0.13 mmol) gave a crude product, which was purified by flash chromatography on a silica gel column with elution of EtOAc/CH_2_Cl_2_ (1:1) to give the desired compound **2** (46 mg, 52% yield). The purity of compound **2** was 97.0% as shown by HPLC on a silica column (Dikma, 10 × 250 mm, 10 μm particle size), elution: EtOAc/hexane = 3:1 at a flow rate of 3.0 mL/min, *t*_R_ = 17.9 min. C_34_H_35_N_5_O_7_S; yellow solid; mp 128.0–129.0 °C; TLC (EtOAc/ CH_2_Cl_2_ = 1:1) *R_f_* = 0.5; IR ν_max_ (neat) 3357, 3197, 2920, 2849, 1653, 1632, 1471 cm^–1^; ^1^H NMR (400 MHz, CDCl_3_) δ 8.35 (s, 1 H), 7.88 (d, *J* = 8.8 Hz, 2 H), 7.82 (d, *J* = 8.9 Hz, 1 H), 7.41 (t, *J* = 7.8 Hz, 1 H), 7.29 (d, *J* = 2.4 Hz, 1 H), 7.01–6.99 (m, 2 H), 6.84 (d, *J* = 8.5 Hz, 1 H), 6.71 (d, *J* = 8.8 Hz, 2 H), 6.44 (t, *J* = 5.4 Hz, 1 H), 4.85 (dd, *J* = 12.1, 5.3 Hz, 1 H), 4.15 (t, *J* = 4.7 Hz, 2 H), 3.86 (t, *J* = 4.7 Hz, 2 H), 3.78–3.64 (m, 6 H), 3.40 (q, *J* = 5.4 Hz, 2 H), 3.01 (s, 6 H), 2.87–2.59 (m, 3 H), 2.09–2.00 (m, 1 H); ^13^C NMR (100 MHz, CHCl_3_) δ 171.1, 169.2, 168.4, 167.6, 166.6, 156.2, 151.9, 149.0, 146.7, 136.0, 135.6, 132.4, 128.5 (2 ×), 122.6, 121.5, 116.7, 115.4, 111.7 (2 ×), 111.6, 110.2, 105.4, 70.9, 70.7, 69.8, 69.5, 68.1, 48.8, 42.3, 40.14 (2 ×), 31.3, 22.7. ESI-HRMS calcd for C_34_H_36_N_5_O_7_S: 658.2330, found: *m/z* 658.2307 [M + H]^+^.

**4-((2-(2-(2-(2-((2-(4-(Dimethylamino)phenyl)benzo[*d*]thiazol-6yl)oxy)ethoxy) ethoxy)ethoxy)ethyl)amino)-2-(2,6-dioxopiperidin-3-yl)isoindoline-1,3-dione (3)**

By a procedure similar to that for compound **1**, the substitution reaction of **10** (88 mg, 0.32 mmol) with **9c** (110 mg, 0.25 mmol) gave a crude product, which was purified by flash chromatography on a silica gel column with elution of EtOAc/CH_2_Cl_2_ (1:1) to give the desired compound **3** (70 mg, 40% yield). The purity of compound **3** was 95.1% as shown by HPLC on a silica column (Dikma, 10 × 250 mm, 10 μm particle size), elution: EtOAc/hexane = 9:1 at a flow rate of 3.0 mL/min, *t*_R_ = 15.9 min. C_36_H_39_N_5_O_8_S; yellow solid; mp 87.5–89.0 °C; TLC (EtOAc/CH_2_Cl_2_ = 1:1) *R_f_* = 0.38; IR ν_max_ (neat) 3358, 3197, 2919, 2849, 1661, 1645, 1622, 1471, 1407 cm^–1^; ^1^H NMR (400 MHz, CDCl_3_) δ 8.55 (t, *J* = 13.5 Hz, 1 H), 7.86 (d, *J* = 8.6 Hz, 2 H), 7.81 (d, *J* = 8.9 Hz, 1 H), 7.39 (t, *J* = 7.8 Hz, 1 H), 7.27 (s, 1 H), 7.05–6.96 (m, 2 H), 6.82 (d, *J* = 8.5 Hz, 1 H), 6.68 (d, *J* = 8.6 Hz, 2 H), 6.42 (t, *J* = 5.1 Hz, 1 H), 4.86 (q, *J* = 4.3 Hz, 1 H), 4.13 (t, *J* = 3.9 Hz, 2 H), 3.84 (t, *J* = 4.6, 2 H), 3.72–3.67 (m, 2 H), 3.67–3.59 (m, 8 H), 3.38 (q, *J* = 5.2 Hz, 2 H), 2.99 (s, 6 H), 2.80–2.60 (m, 3 H), 2.04 (t, *J* = 6.3 Hz,1 H); ^13^C NMR (100 MHz, CDCl_3_) δ 171.2, 169.2, 168.5, 167.5, 166.5, 156.2, 151.9, 148.6, 146.7, 135.9, 135.4, 132.4, 128.5 (2 ×), 122.5, 121.2, 116.7, 115.4, 111.7 (2 ×), 111.5, 110.1, 105.4, 70.7, 70.6 (3 ×), 69.6, 69.4, 68.1, 48.8, 42.3, 40.1 (2 ×), 31.3, 22.6. ESI-HRMS calcd for C_36_H_40_N_5_O_8_S: 702.2592, found: *m/z* 702.2589 [M + H]^+^.

**4-((14-((2-(4-(Dimethylamino)phenyl)benzo[*d*]thiazol-6-yl)oxy)-3,6,9,12-tetraoxatetradecyl)amino)-2-(2,6-dioxopiperidin-3-yl)isoindoline-1,3-dione (4)**

By a procedure similar to that for compound **1**, the substitution reaction of **10** (102 mg, 0.37mmol) with **9d** (90 mg, 0.18 mmol) gave a crude product, which was purified by flash chromatography on a silica gel column with elution of EtOAc/CH_2_Cl_2_(2:1) to give the desired compound **4** (89 mg, 65% yield). The purity of compound **4** was 99.3% as shown by HPLC on a silica column (Dikma, 10 × 250 mm, 10 μm particle size), elution: EtOAc/MeOH = 99:1 at a flow rate of 3.0 mL/min, *t*_R_ = 11.3 min. C_38_H_43_N_5_O_9_S; yellow solid; mp 77.5–79.0 °C; TLC (EtOAc/ CH_2_Cl_2_ = 2:1) *R_f_* = 0.38; IR ν_max_ (neat) 3356, 3197, 2921, 2851, 1653, 1634, 1470, 1456, 1368, 742, 701 cm^–1^; ^1^H NMR (400 MHz, CDCl_3_) δ 8.66 (s, 1 H), 7.85 (d, *J* = 8.8 Hz, 2 H), 7.80 (d, *J* = 8.9 Hz, 1 H), 7.40 (t, *J* = 7.8 Hz, 1 H), 7.27 (d, *J* = 2.4 Hz, 1 H), 7.05–6.96 (m, 2 H), 6.83 (d, *J* = 8.6 Hz, 1 H), 6.68 (d, *J* = 8.8 Hz, 2 H), 6.42 (t, *J* = 5.4 Hz, 1 H), 4.85 (q, *J* = 5.2 Hz, 1 H), 4.14 (t, *J* = 4.7 Hz, 2 H), 3.84 (t, *J* = 4.7, 2 H), 3.73–3.67 (m, 2 H), 3.67–3.57 (m, 12 H), 3.38 (q, *J* = 5.4 Hz, 2 H), 2.99 (s, 6 H), 2.85–2.60 (m, 3 H), 2.07–1.97 (m, 1 H); ^13^C NMR (100 MHz, CDCl_3_) δ 171.3, 169.1, 168.5, 167.5, 166.5, 156.1, 151.8, 148.7, 146.7, 135.9, 135.5, 132.4, 128.4 (2 ×), 122.5, 121.3, 116.7, 115.4, 111.7 (2 ×), 111.5, 110.1, 105.3, 70.8, 70.6, 70.5 (2 ×), 70.4 (2 ×), 69.6, 69.3, 68.0, 48.7, 42.2, 40.1 (2 ×), 31.3, 22.6. ESI-HRMS calcd for C_38_H_44_N_5_O_9_S: 746.2854, found: *m/z* 746.2888 [M + H]^+^.

**6-Azidohexyl methanesulfonate (S9)**

A mixture of 6-bromohexanol (827 mg, 4.5 mmol) and NaN_3_ (585 mg, 9 mmol) in DMF (23 mL) was stirred at 80 °C for 16 h. The mixture was concentrated under reduced pressure. The mixture was extracted with EtOAc and H_2_O. The organic phase was dried over MgSO_4_, filtered, concentrated under reduced pressure to give 6-azidohexanol. A solution of 6-azidohexanol in anhydrous CH_2_Cl_2_ (9 mL) at 0 °C was added Et_3_N (1.9 mL, 14 mmol) and methanesulfonyl chloride (0.7 mL, 9 mmol). The mixture was stirred at room temperature for 2 h. The mixture was extracted with EtOAc and H_2_O. The combined organic phase was dried over MgSO_4_, filtered, concentrated under reduced pressure and purified by flash chromatography on a silica gel column with elution of EtOAc/hexane (2:3) to give compound **S9** (626 mg, 63% yield for two steps). C_7_H_15_N_3_O_3_S; colorless liquid; TLC (EtOAc/hexane = 2:3) *R_f_* = 0.53; ^1^H NMR (400 MHz, CDCl_3_) δ 4.15 (t, *J* = 6.3 Hz, 2 H), 3.21 (t, *J* = 6.8 Hz, 2 H), 2.93 (s, 3 H), 1.69 (t, *J* = 6.7 Hz, 2 H), 1.54 (t, *J* = 6.7 Hz, 2H), 1.41−1.32 (m, 4 H); ^13^C NMR (100 MHz, CDCl_3_) δ 69.7, 51.0, 37.1, 28.8, 28.4, 25.9, 24.8. ESI-HRMS calcd for C_7_H_15_N_3_NaO_3_S: 244.0726, found m/z 244.0726 [M + Na]^+^.

**4-(6-((6-Aminohexyl)oxy)benzo[*d*]thiazol-2-yl)-*N*,*N*-dimethylaniline (S10)**

A mixture of compound **8** (200 mg, 0.74 mmol) and K_2_CO_3_ (204 mg, 1.48 mmol) in anhydrous DMF (7 mL) was stirred for 30 min at room temperature, and mesylated compound **S9** (269 mg, 1.11 mmol) was added to the solution. The mixture was stirred at 80 °C for 20 h, cooled, and extracted with EtOAc and H_2_O. The combined organic phase was dried over MgSO_4_, filtered, concentrated under reduced pressure, and purified by flash chromatography on a silica gel column with elution of CH_2_Cl_2_/hexane (2:1) to give the alkylation compound (182 mg).

The above-prepared compound (160 mg, 0.4 mmol) in THF (5 mL) was stirred with PPh_3_ (213 mg, 2 mmol) and H_2_O (0.02 mL, 1.2 mmol) at room temperature for 24 h. The mixture was concentrated under reduced pressure, and purified by flash chromatography on a silica gel column with elution of CH_2_Cl_2_/MeOH (1:9) to give compound **S10** (125 mg, 53% yield for two steps). C_21_H_27_N_3_OS; white solid; TLC (CH_2_Cl_2_/MeOH = 9:1) *R_f_* = 0.05; ^1^H NMR (400 MHz, CDCl_3_) δ 7.86 (d, *J* = 8.8 Hz, 2 H), 7.82 (d, *J* = 8.9 Hz, 1 H), 7.26 (d, *J* = 2.3 Hz, 1 H), 6.99 (dd, *J* = 8.9, 2.3 Hz, 1 H), 6.69 (d, *J* = 8.8 Hz, 2 H), 3.96 (t, *J* = 6.5 Hz, 2 H), 2.98 (s, 6 H), 2.67 (t, *J* = 6.1 Hz, 2 H), 1.78 (quin, *J* = 6.9 Hz, 2 H), 1.54 (s, 2 H), 1.49−1.35 (m, 6 H); ^13^C NMR (100 MHz, CDCl_3_) δ 166.2, 156.5, 151.8, 148.8, 135.7, 128.4 (2 ×), 122.6, 121.6, 115.3, 111.7 (2 ×), 105.1, 68.5, 42.0, 40.1 (2 ×), 33.6, 29.2, 26.6, 25.8. ESI-HRMS calcd for C_21_H_28_N_3_OS: 370.1948, found *m*/*z* 370.1942 [M + H]^+^.

**4-((6-((2-(4-(Dimethylamino)phenyl)benzo[d]thiazol-6-yl)oxy)hexyl)amino)-2-(2,6-dioxopiperidin-3-yl)isoindoline-1,3-dione (compound S11)**

A mixture of compound **S10** (50 mg, 0.14 mmol), compound **10** (41 mg, 0.15 mmol) and DIPEA (0.05 mL, 0.27 mmol) in NMP (1 mL) was stirred at 90 °C for 24 h. The mixture was extracted with EtOAc and H_2_O. The combined organic phase was dried over MgSO4, filtered, concentrated under reduced pressure, and purified by flash chromatography on a silica gel column with elution of EtOAc/CH_2_Cl_2_ (1:9) to give compound **S11** (33 mg, 39%). C_34_H_35_N_5_O_5_S; yellow solid; TLC (EtOAc/CH_2_Cl_2_ = 1:9) *R_f_* = 0.5; ^1^H NMR (400 MHz, CDCl_3_) δ 8.04 (s, 1H), 7.89 (d, *J* = 9.0 Hz, 2 H), 7.84 (d, *J* = 9.2 Hz, 1 H), 7.46 (t, *J* = 7.6 Hz, 1 H), 7.28 (s, 1 H), 7.06 (d, *J* = 7.8 Hz, 1 H), 7.01 (dd, *J* = 9.2, 1.6 Hz, 1 H), 6.86 (d, *J* = 7.8 Hz, 2 H), 6.22 (s, 1 H), 4.94−4.78 (m, 1 H), 4.00 (t, *J* = 2.2 Hz, 2 H), 3.27 (q, *J* = 5.6 Hz, 2 H), 3.03 (s, 6 H), 2.91−2.67 (m, 3 H), 2.09 (t, *J* = 6.0 Hz, 2 H), 1.82 (quin, *J* = 6.0 Hz, 2 H), 1.70 (quin, *J* = 6.5 Hz, 2 H), 1.56−1.45 (m, 4 H); ^13^C NMR (100 MHz, DMSO-*d_6_*) δ 172.9, 170.2, 169.0, 167.4, 165.4, 156.2, 152.0, 148.3, 146.5, 136.4, 135.3, 132.3, 128.1 (2 ×), 122.4, 120.5, 117.3, 115.6, 111.9 (2 ×), 110.5, 109.1, 105.6, 68.1 (2 ×), 48.6, 41.8 (2 ×), 31.0, 28.7, 28.6, 26.1, 25.4, 22.2. ESI-HRMS calcd for C_34_H_36_N_5_O_5_S: 626.2432, found *m*/*z* 626.2424 [M + H]^+^.

***N*,*N*-Dimethyl-4-(6-(prop-2-yn-1-yloxy)benzothiazol-2-yl)aniline (S13)**

A mixture of 6-hydroxy substituted BTA compound **8** (100 mg, 0.37 mmol) and potassium carbonate (102 mg, 0.74 mmol) in DMF (2 mL) was stirred for 15 min. Propargyl bromide (56 μL, 0.74 mmol) was added, and the mixture was heated at 70 ^o^C for 18 h. The reaction was quenched with 1 M HCl. The mixture was concentrated under reduced pressure. The organic phase was dried over MgSO_4_, filtered, and concentrated under reduced pressure to give compound **S13** (100 mg, 88% yield). C_18_H_16_N_2_OS; pale yellow solid; UV-vis (DMSO) λ_max_ = 362 nm (ε = 17805 M^–1^ cm^–1^); ^1^H NMR (400 MHz, CD_3_OD) δ 7.89 (2 H, d, *J* = 9.2 Hz), 7.87 (1 H, d, *J* = 8.9 Hz), 7.40 (1 H, d, *J* = 2.1 Hz), 7.07 (1 H, d, *J* = 8.9 Hz), 6.74 (2 H, d, *J* = 9.2 Hz), 4.74 (2 H, s), 3.02 (6 H, s), 2.53 (1 H, s); ^13^C NMR (100 MHz, CD_3_Cl) δ 167.1, 154.9, 151.9, 135.5, 128.6, 126.4, 122.7, 121.4, 115.6, 111.7, 111.6, 106.1, 78.4, 75.8, 56.5, 40.2(2×), 30.9; ESI–HRMS calcd for C_18_H_16_N_2_OS: 307.0904, found *m/z* 307.0899 [M + H]^+^.

***N*-(2-(2,6-Dioxopiperidin-3-yl)-1-oxoisoindolin-4-yl)-2-iodoacetamide (S16)**

Iodoacetyl chloride (34.3 μL, 0.386 mmol) was added dropwise to solution of lenalidomide (**S14)** (100 mg, 0.39 mmol) in anhydrous THF (2 mL). The mixture was stirred at room temperature for 1 h, washed with saturated NaHCO_3(aq)_, and triturated with Et_2_O. The solids were collected by suction filtration, and rinsed with Et_2_O to give compound **S16** (155 mg, 94% yield). C_15_H_14_IN_3_O_4_; yellow solid; mp 284–286 ^o^C; ^1^H NMR (400 MHz, CD_3_OD) δ 11.03 (1 H, s), 10.20 (1 H, s), 7.78 (1 H, d, *J* = 7.5 Hz), 7.53 (1 H, t, *J* = 7.5 Hz), 7.51 (1 H, s), 5.15 (1 H, dd, *J* = 13.2, 5.2 Hz), 3.88 (2 H, s), 4.41–4.28 (2 H, m), 2.92–2.90 (1 H, m), 2.48–2.43 (1 H, m), 2.11–2.08 (2 H, m); ^13^C NMR (100 MHz, DMSO-*d*_6_) δ 172.9, 171.1, 168.4, 167.8, 133.8, 133.4, 132.7, 128.7, 125.3, 119.3, 51.5, 46.5, 40.1, 29.1, 22.7; ESI–HRMS calcd for C_15_H_14_IN_3_O_4_: 425.995, found *m/z* 425.992 [M + H]^+^.

***N*-(2-(2,6-Dioxopiperidin-3-yl)-1-oxoisoindolin-4-yl) 2-azido-acetamide (S17)**

A mixture of compound **S16** (100 mg, 0.23 mmol) and sodium azide (30.4 mg, 0.47 mmol) in DMF (2 mL) was stirred at 80 ^o^C for 24 h. The mixture was concentrated under reduced pressure and then extracted with EtOAc and H_2_O.The organic phase was dried over MgSO_4_, filtered, and concentrated under reduced pressure to give compound **S17** (71 mg, 89% yield). C_15_H_14_N_6_O_4_; white solid; mp 172–174 ^o^C; UV-vis (DMSO) λ_max_ = 260 nm (ε = 6219 M^–1^ cm^–1^); ^1^H NMR (400 MHz, CD_3_OD) δ 11.03 (1 H, s), 10.09 (1 H, s), 7.83 (1 H, t, *J* = 7.5 Hz), 7.11 (1 H, d, *J* = 7.5 Hz), 6.91 (1 H, d, *J* = 7.5 Hz), 5.15 (1 H, dd, *J* = 13.2, 5.2 Hz), 4.42–4.32 (2 H, m), 4.10 (2 H, s), 2.92–2.90 (1 H, m), 2.80–2.79 (1 H, m), 2.36–2.33 (1 H, m), 2.04–2.01 (1 H, m); ^13^C NMR (100 MHz, DMSO-*d*_6_) δ 172.9, 171.1, 167.8, 166.7, 132.9, 132.8, 128.9, 125.5, 119.7, 116.9, 51.6, 51.1, 46.5, 31.3, 22.7; ESI–HRMS calcd for C_15_H_14_N_6_O_4_: 341.0998, found *m/z* 341.0997 [M + H]^+^.

**2-(4-(((2-(4-(Dimethylamino)phenyl)benzothiazol-6-yl)oxy)methyl)-1*H*-1,2,3-triazol-1-yl)acetamide (S18)**

A mixture of alkyne compound **S13** (50 mg, 0.16 mmol), azide compound **S17** (50 mg, 0.15 mmol), sodium ascorbate (26 mg, 0.13 mmol) and CuSO_4_ (7 mg, 0.044 mmol) in THF/H_2_O (2:1) was stirred at room temperature for 12 h. The mixture was concentrated under reduced pressure, and then triturated with H_2_O, hexane, EtOAc to give compound **S18** (84 mg, 88% yield). C_33_H_30_N_8_O_5_S; pale yellow solid; mp 223–238 ^o^C; UV-vis (DMSO) λ_max_ = 362 nm (ε = 19824 M^–1^ cm^–1^); ^1^H NMR (400 MHz, DMSO-*d*_6_) δ 11.03 (1 H, s), 10.40 (1 H, s), 8.30 (1 H, s), 7.85 (1 H, s), 7.84 (2 H, d, *J* = 9.2 Hz), 7.77 (1 H, d, *J* = 2.1 Hz), 7.55 (1 H, t, *J* = 7.5 Hz), 7.53 (1 H, d, *J* = 7.5 Hz), 6.80 (2 H, d, *J* = 7.5 Hz), 5.43 (2 H, d, *J* = 7.8 Hz), 5.25 (2 H, d, *J* = 8.2 Hz), 5.15 (1 H, dd, *J* = 13.2, 5.2 Hz), 4.38 (2 H, s), 4.46–4.35 (2 H, m), 3.01 (6 H, s), 2.93–2.87 (1 H, m), 2.76–2.74 (1 H, m), 2.35–2.32 (1 H, m), 2.08–2.03 (1 H, m); ^13^C NMR (100 MHz, DMSO-*d*_6_) δ 173.1, 171.2, 167.9, 165.8, 164.7, 155.6, 152.1, 148.6, 135.2, 133.8, 133.0, 132.9, 129.1 (2×), 128.5, 128.3, 126.7, 125.3, 122.6, 120.4, 119.8, 115.9, 111.9 (2×), 106.2, 72.3, 61.7, 60.4, 52.0, 51.7, 46.6, 31.3, 22.8; ESI–HRMS calcd for C_33_H_30_N_8_O_5_S: 649.1981, found *m/z* 649.1985 [M + H]^+^.

**Supplementary References**

1. Ioka, S.; Saitoh, T.; Iwano, S.; Suzuki, K.; Maki, S. A.; Miyawaki, A.; Imoto, M.; Nishiyama, S. Synthesis of Firefly Luciferin Analogues and Evaluation of the Luminescent Properties. *Chemistry* **2016**, *22*, 9330–9337.
2. Auld, D. S.; Zhang, Y. Q.; Southall, N. T.; Rai, G.; Landsman, M.; MacLure, J.; Langevin, D.; Thomas, C. J.; Austin, C. P.; Inglese, J. A Basis for Reduced Chemical Library Inhibition of Firefly Luciferase Obtained from Directed Evolution. *J. Med. Chem*. **2009**, *52*, 1450–1458.
3. Qin, L.; Vastl, J.; Gao, J. Highly sensitive amyloid detection enabled by thioflavin T dimers. *Mol. Biosyst*. **2010**, *6*, 1791–1795.
4. Yu, X.; Eymur, S.; Singh, V.; Yang, B.; Tonga, M.; Bheemaraju, A.; Cooke, G.; Subramani, C.; Venkataraman, D.; Stanley, R. J.; Rotello, V. M. Flavin as a Photo-Active Acceptor for Efficient Energy and Charge Transfer in a Model Donor-acceptor System. *Phys. Chem. Chem. Phys.* **2012**, *14*, 6749–6754.
5. Faust, A.; Waschkau, B.; Waldeck, J.; Holtke, C.; Breyholz, H. J.; Wagner, S.; Kopka, K.; Schober, O.; Heindel, W.; Schafers, M.; Bremer, C. Synthesis and Evaluation of a Novel Hydroxamate Based Fluorescent Photoprobe for Imaging of Matrix Metalloproteinases. *Bioconjug. Chem.* **2009**, *20*, 904–912.
6. Jensen, M.; Schmidt, S.; Fedosova, N. U.; Mollenhauer, J.; Jensen, H. H. Synthesis and Evaluation of Cardiac Glycoside Mimics as Potential Anticancer Drugs. *Bioorg. Med. Chem.* **2011**, *19*, 2407–2417.
7. Ishoey, M.; Chorn, S.; Singh, N.; Jaeger, M. G.; Brand, M.; Paulk, J.; Bauer, S.; Erb, M. A.; Parapatics, K.; Muller, A. C.; Bennett, K. L.; Ecker, G. F.; Bradner, J. E.; Winter, G. E. Translation Termination Factor GSPT1 is a Phenotypically Relevant Off-Target of Heterobifunctional Phthalimide Degraders. *ACS Chem. Biol.* **2018**, *13*, 553–560.


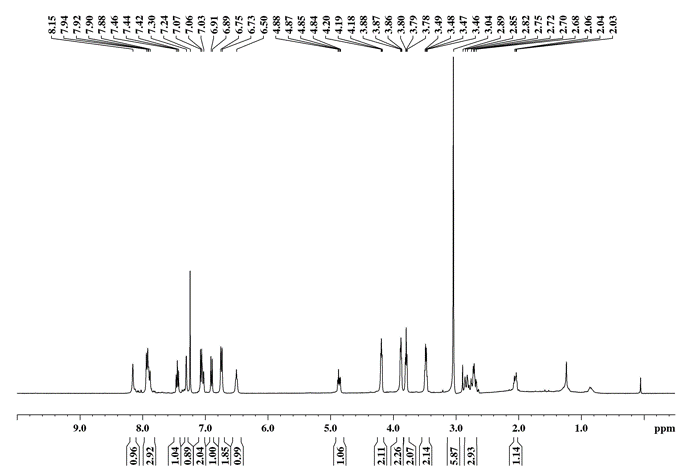


^1^H NMR spectrum of compound **1** (400 MHz, CDCl_3_)


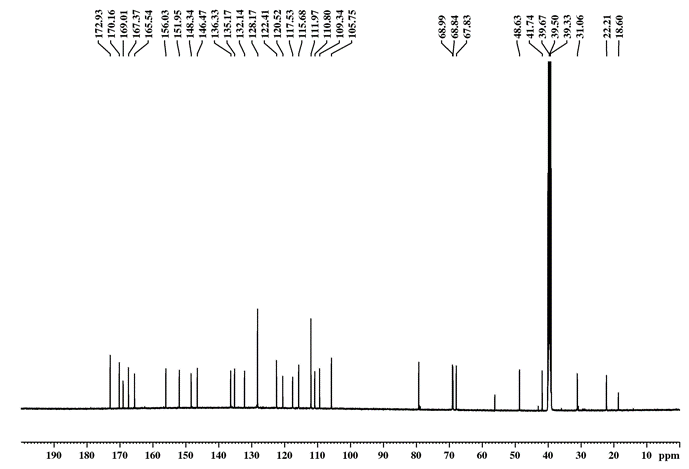


^13^C NMR spectrum of compound **1** (100 MHz, DMSO-*d*_6_)


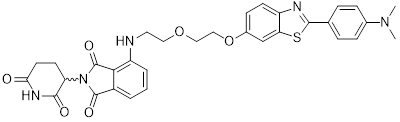

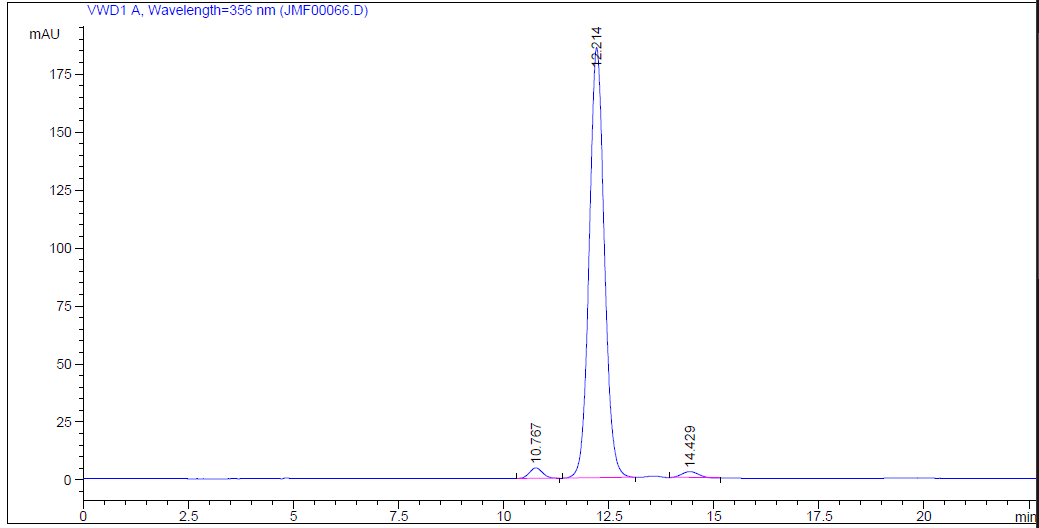


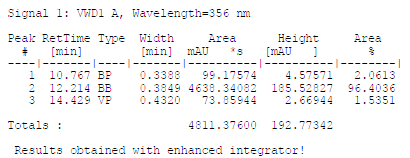


HPLC diagram of compound **1**. Silica column (Dikma, 10 × 250 mm, 10 μm particle size); elution: EtOAc/hexane = 3:1 at a flow rate of 3.0 mL/min, *t*_R_ = 12.2 min, purity 96.4%.


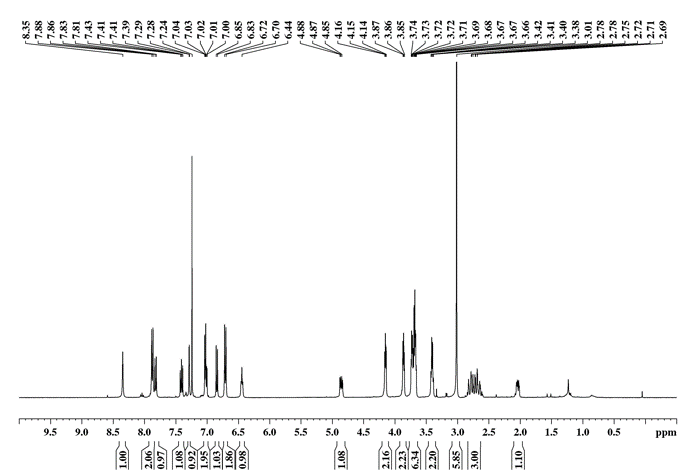


^1^H NMR spectrum of compound **2** (400 MHz, CDCl_3_)


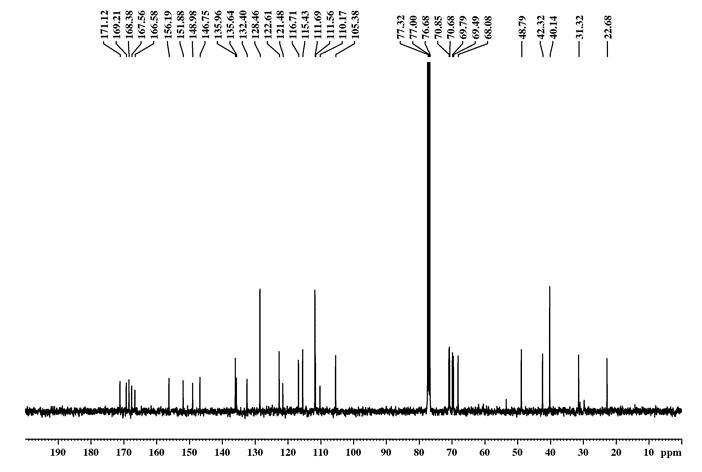


^13^C NMR spectrum of compound **2** (100 MHz, CDCl_3_)


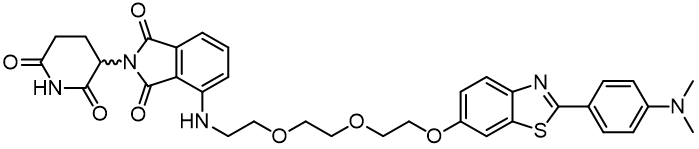

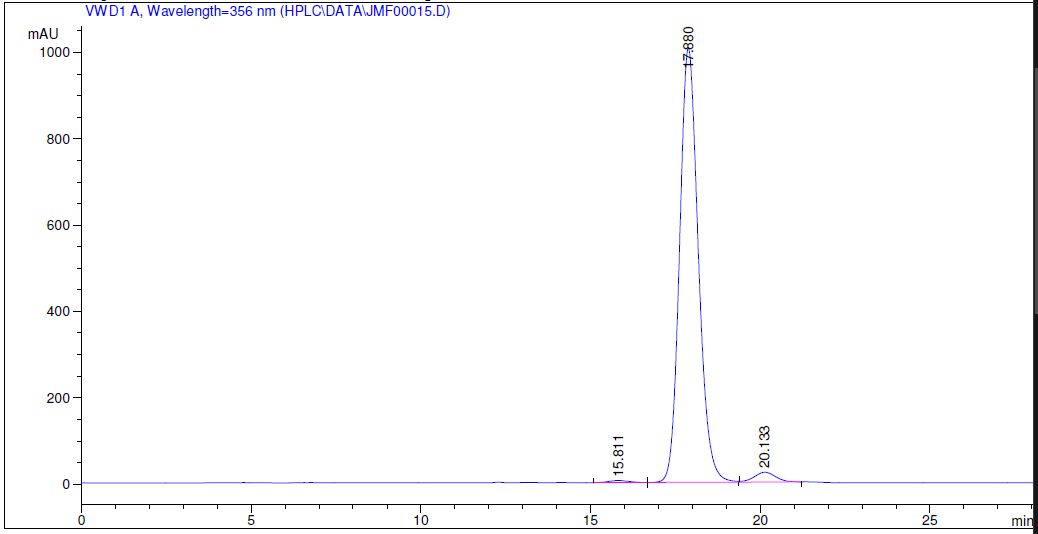


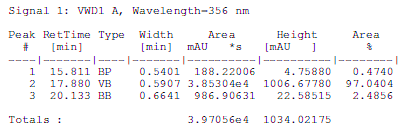


HPLC diagram of compound **2**. Silica column (Dikma, 10 × 250 mm, 10 μm particle size); elution: EtOAc/hexane = 3:1 at a flow rate of 3.0 mL/min, *t*_R_ = 17.9 min, purity 97.0%.


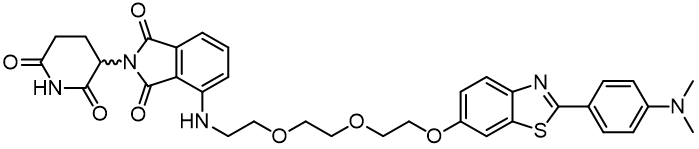


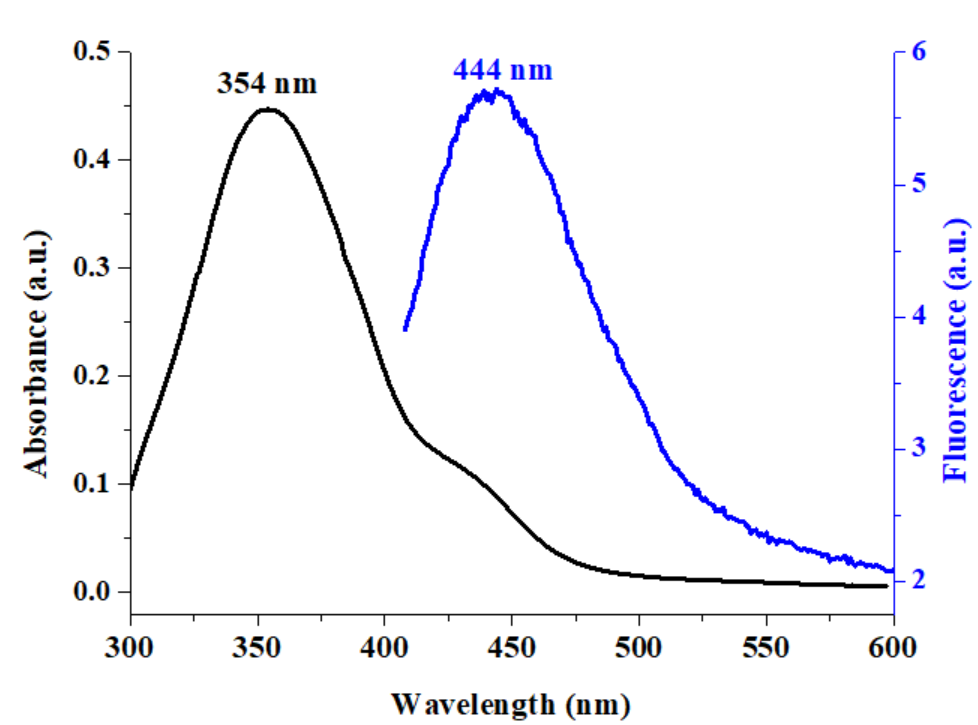


Absorption and fluorescence spectra (λ_ex_ = 375 nm) of compound **2**

at 17 μM in DMSO/H_2_O (1:100)


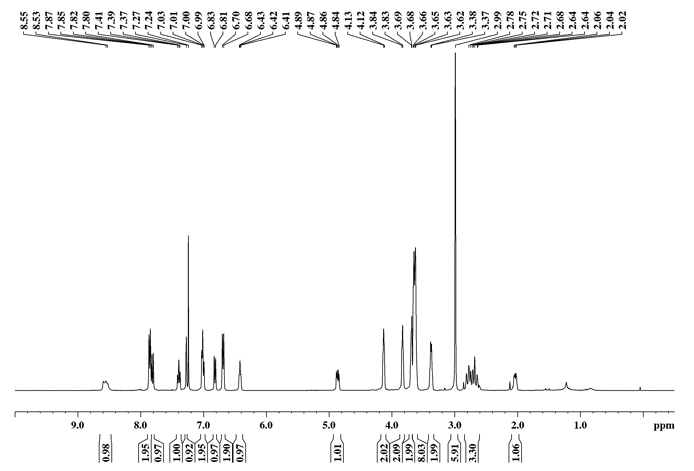


^1^H NMR spectrum of compound **3** (400 MHz, CDCl_3_)


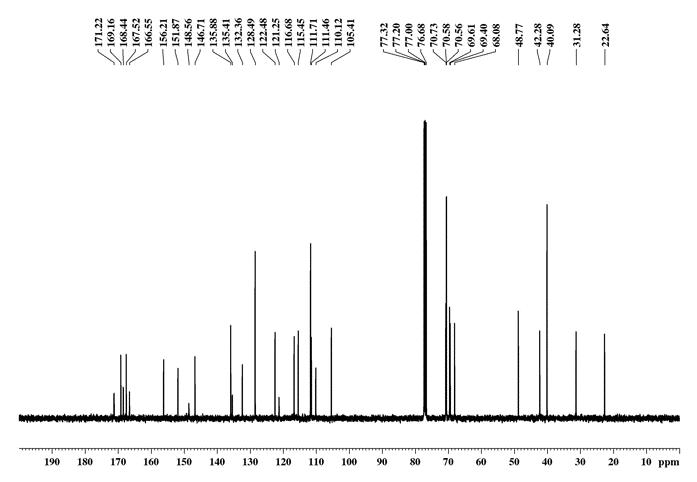


^13^C NMR spectrum of compound **3** (100 MHz, CDCl_3_)


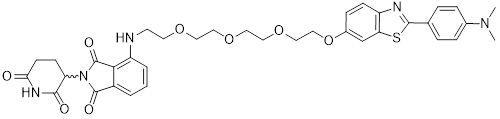

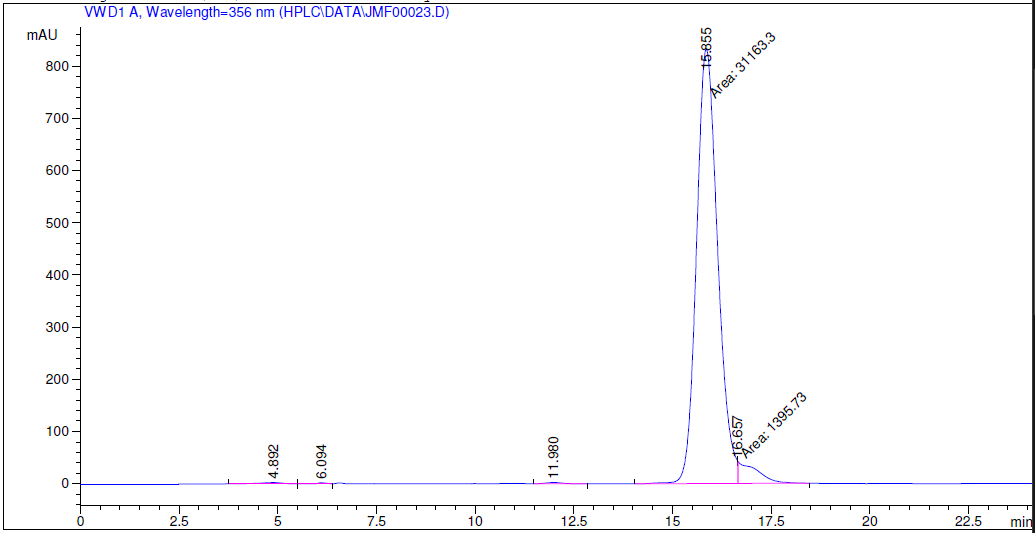


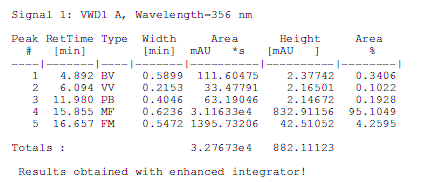


HPLC diagram of compound **3**. Silica column (Dikma, 10 × 250 mm, 10 μm particle size); elution: EtOAc/hexane = 9:1 at a flow rate of 3.0 mL/min, *t*_R_ = 15.9 min, purity 95.1%.


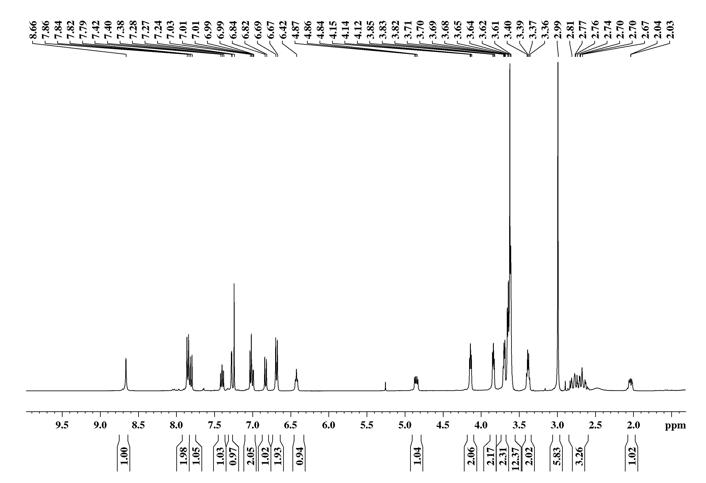


^1^H NMR spectrum of compound **4** (400 MHz, CDCl_3_)


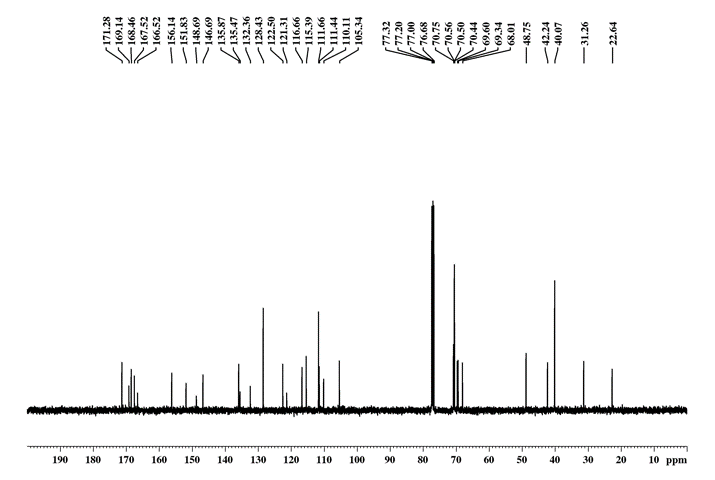


^13^C NMR spectrum of compound **4** (100 MHz, CDCl_3_)


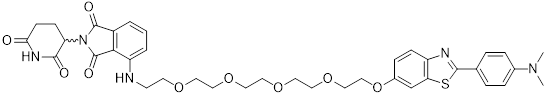

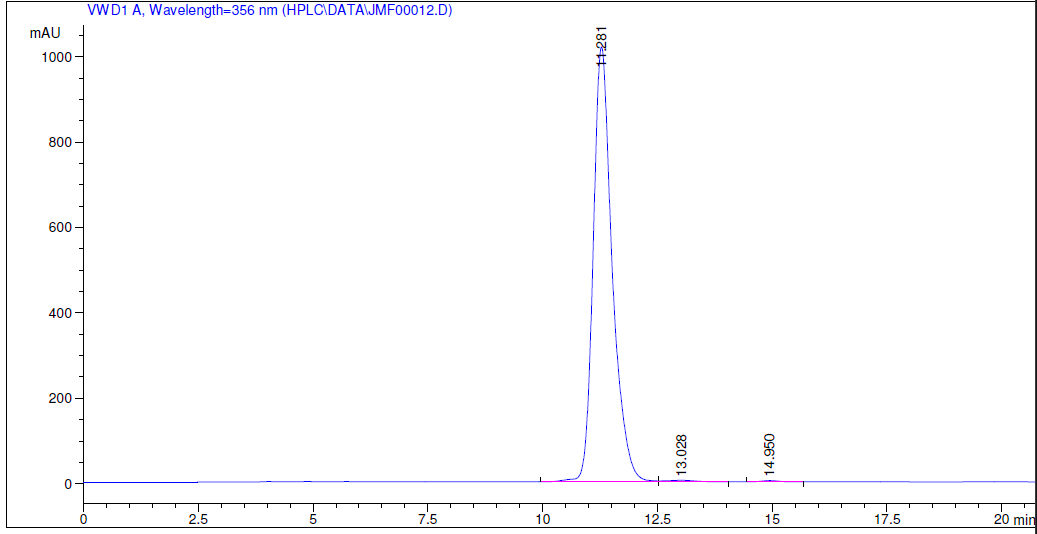


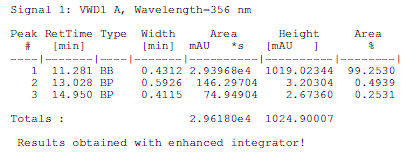


HPLC diagram of compound **4**. Silica column (Dikma, 10 × 250 mm, 10 μm particle size); elution: EtOAc/MeOH = 99:1 at a flow rate of 3.0 mL/min, *t*_R_ = 11.3 min, purity 99.3%.


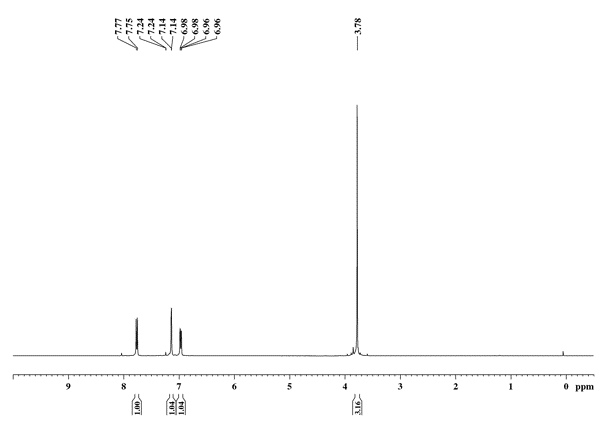


^1^H NMR spectrum of compound **5** (400 MHz, CDCl_3_)


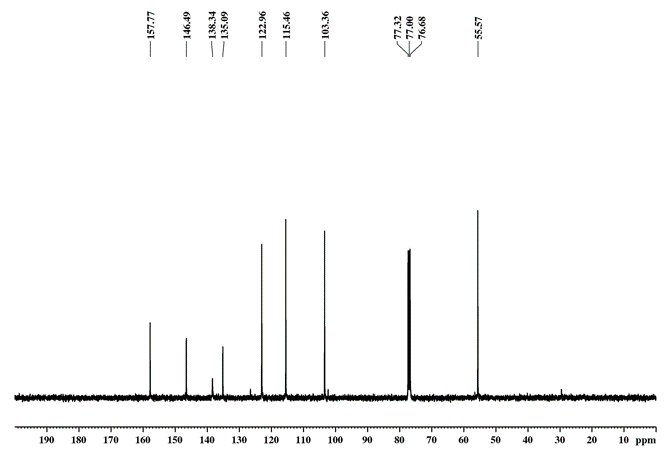


^13^C NMR spectrum of compound **5** (100 MHz, CDCl_3_)


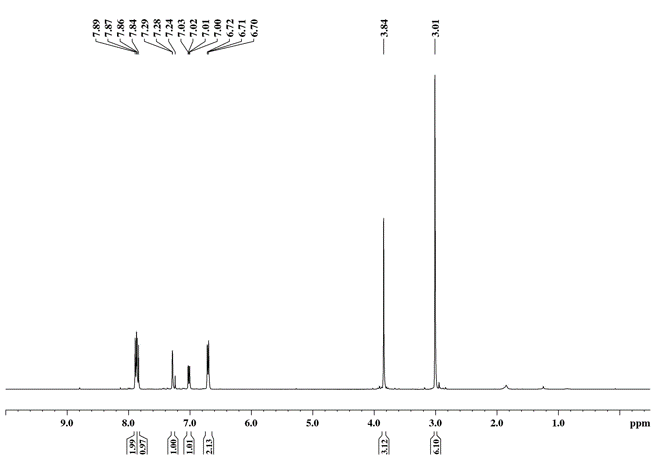


^1^H NMR spectrum of compound **7** (400 MHz, CDCl_3_)


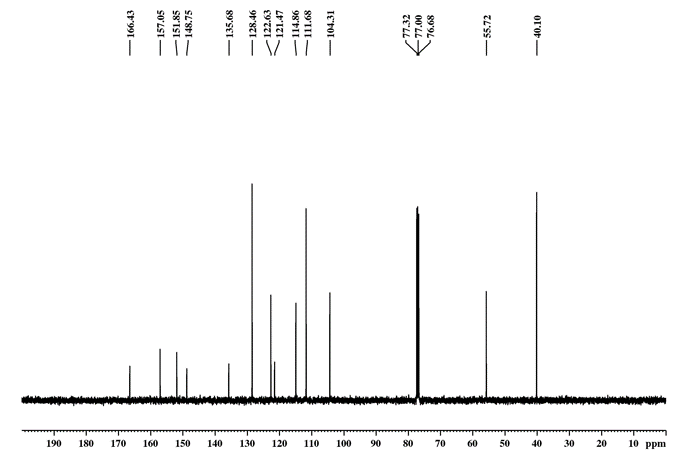


^13^C NMR spectrum of compound **7** (100 MHz, CDCl_3_)


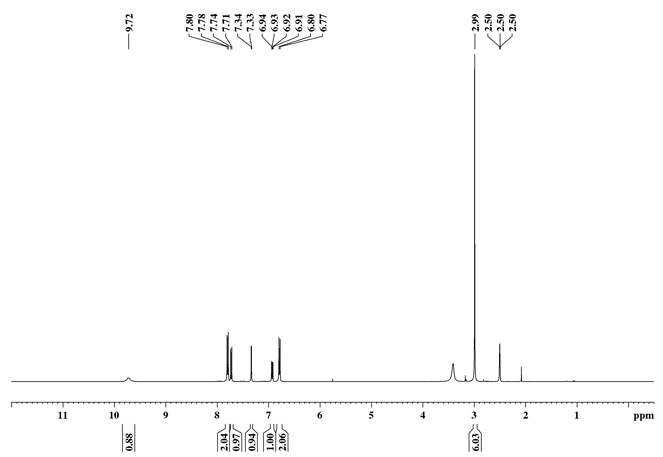


^1^H NMR spectrum of compound **8** (400 MHz, DMSO-*d*_6_)


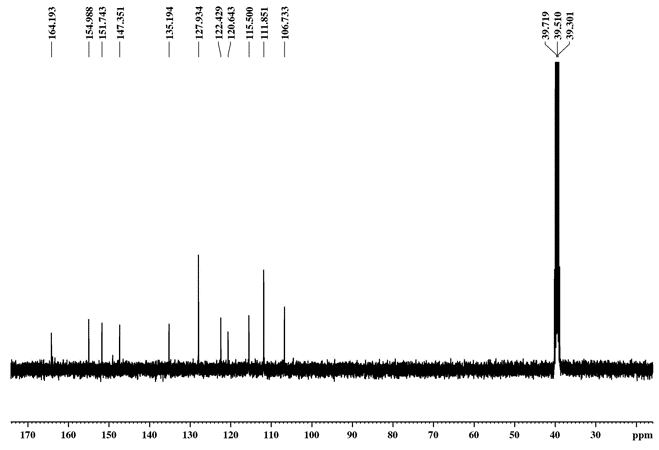


^13^C NMR spectrum of compound **8** (100 MHz, DMSO-*d*_6_)
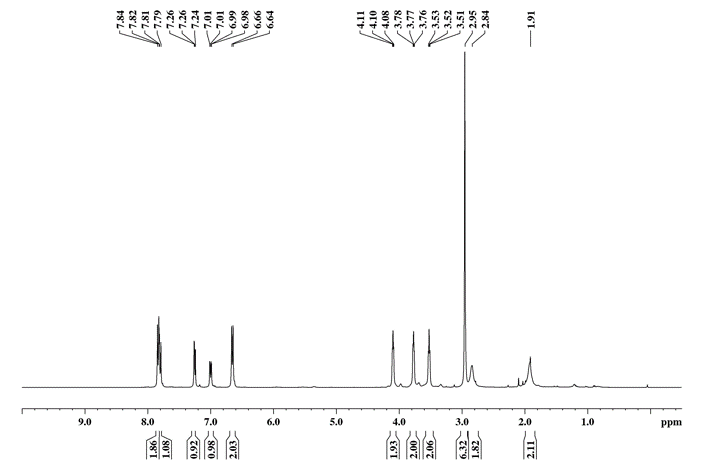


^1^H NMR spectrum of compound **9a** (400 MHz, CDCl_3_)


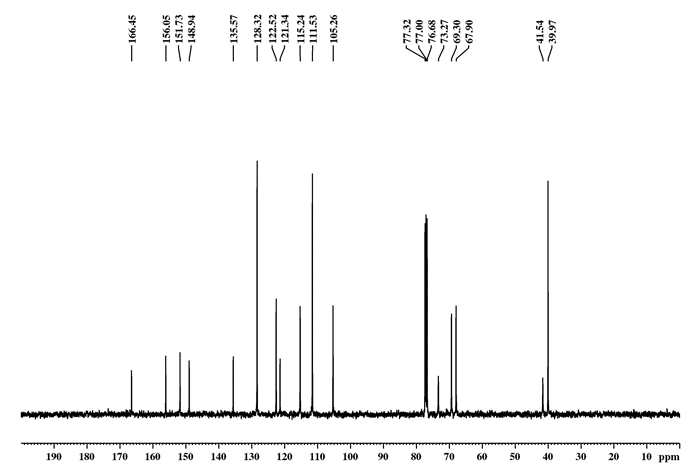


^13^C NMR spectrum of compound **9a** (100 MHz, CDCl_3_)


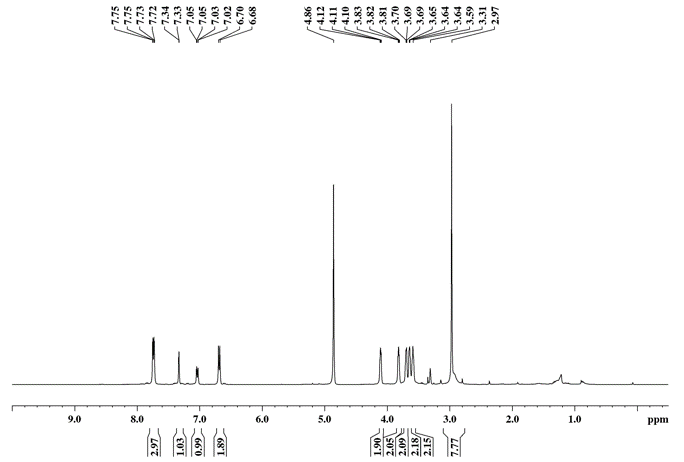


^1^H NMR spectrum of compound **9b** (400 MHz, CD_3_OD)


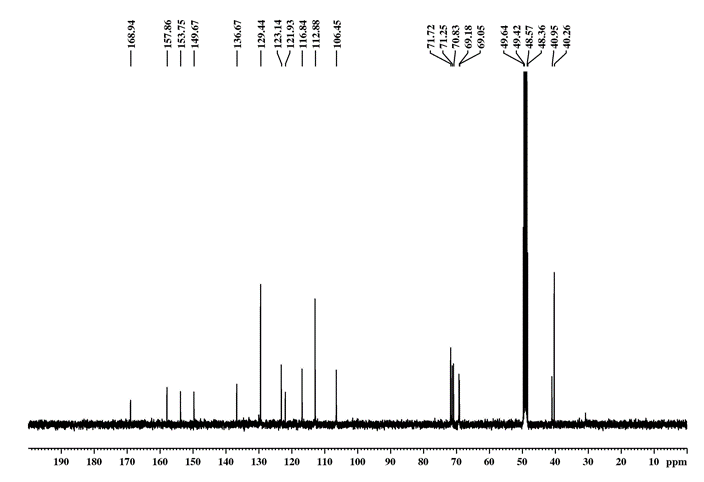


^13^C NMR spectrum of compound **9b** (100 MHz, CD_3_OD)
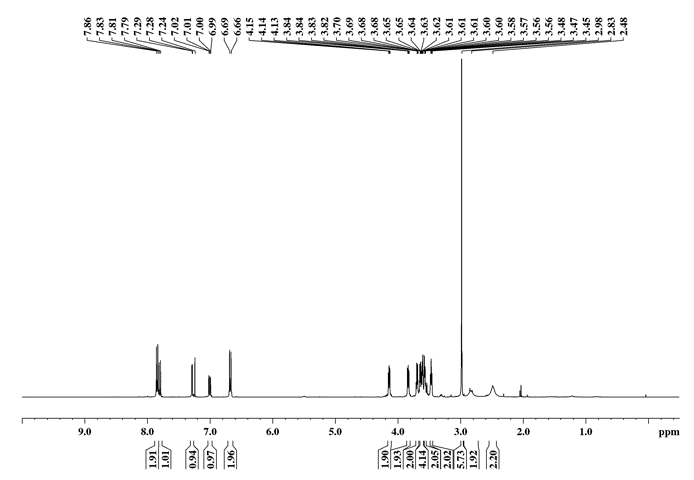


^1^H NMR spectrum of compound **9c** (400 MHz, CDCl_3_)


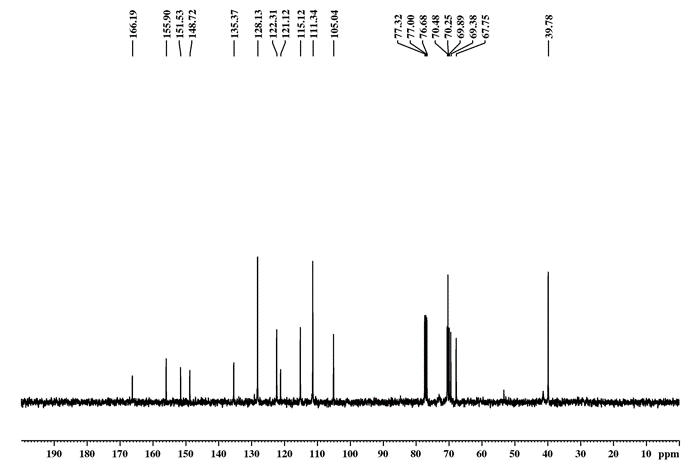


^13^C NMR spectrum of compound **9c** (100 MHz, CDCl_3_)


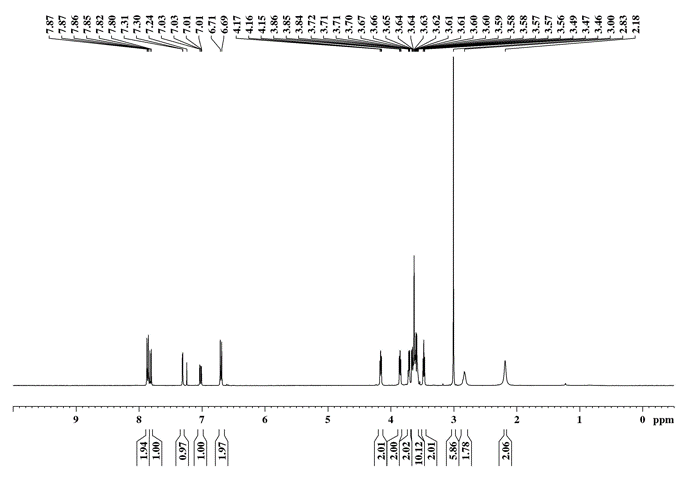


^1^H NMR spectrum of compound **9d** (400 MHz, CDCl_3_)


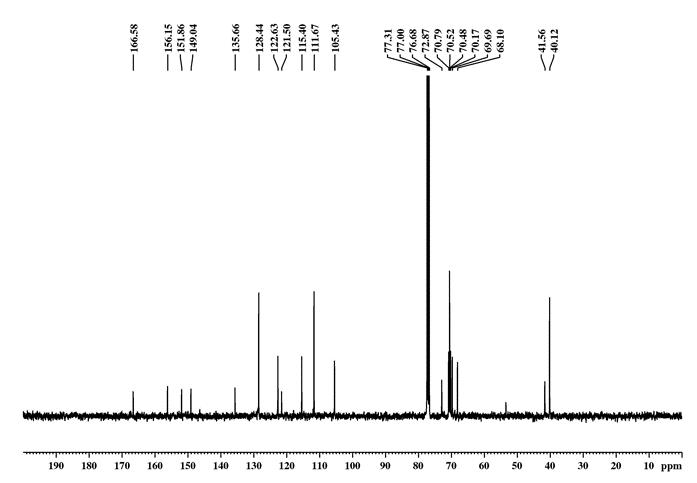


^13^C NMR spectrum of compound **9d** (100 MHz, CDCl_3_)


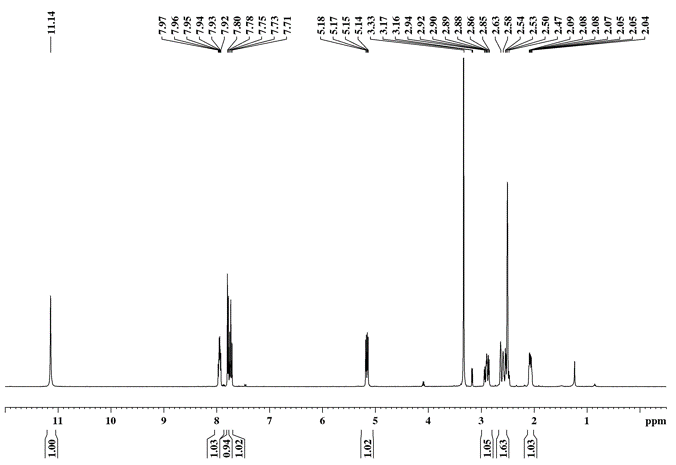


^1^H NMR spectrum of compound **10** (400 MHz, DMSO-*d*_6_)

^^
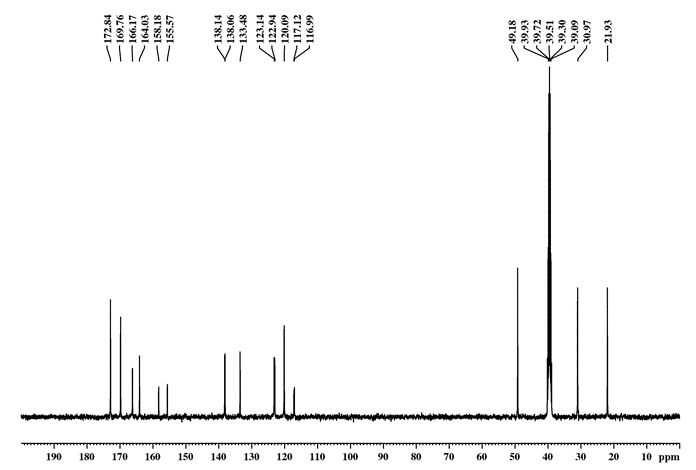


^13^C NMR spectrum of compound **10** (100 MHz, DMSO-*d*_6_)

^^
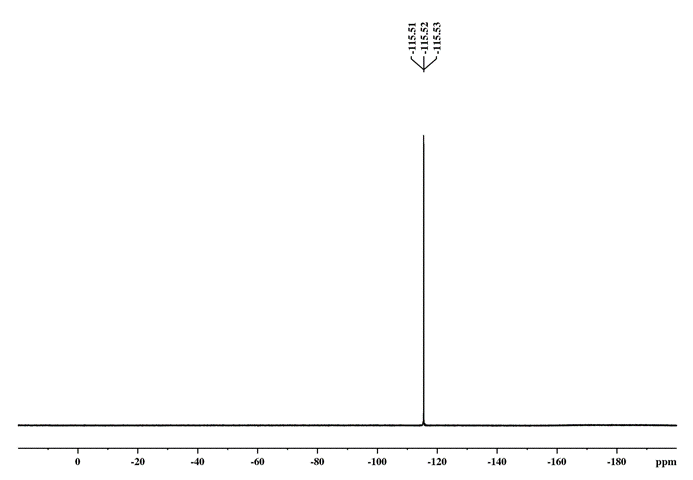


^19^F NMR spectrum of compound **10** (376 MHz, DMSO-*d*_6_)


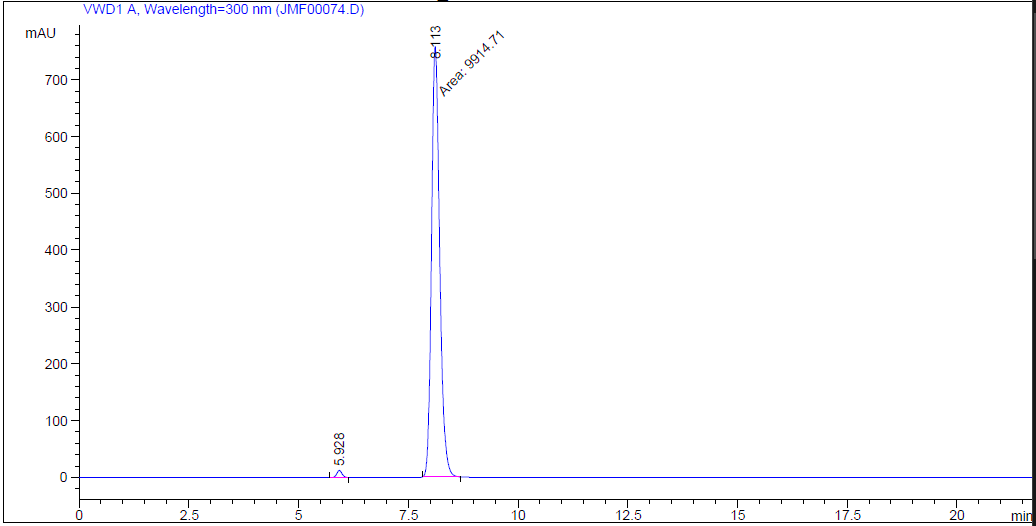


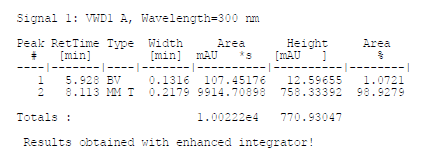


HPLC diagram of compound **10**. Silica column (Dikma, 10 × 250 mm, 10 μm particle size); elution: EtOAc/hexane = 4:1 at a flow rate of 3.0 mL/min, *t*_R_ = 8.1 min, purity 98.9%.


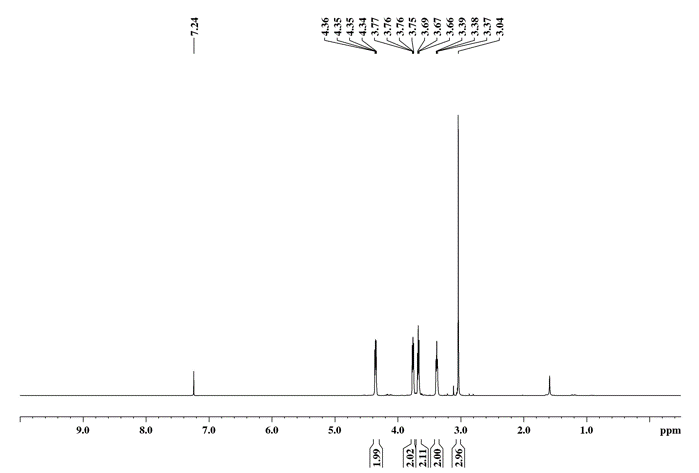


^1^H NMR spectrum of compound **S1a** (400 MHz, CDCl_3_)

^^
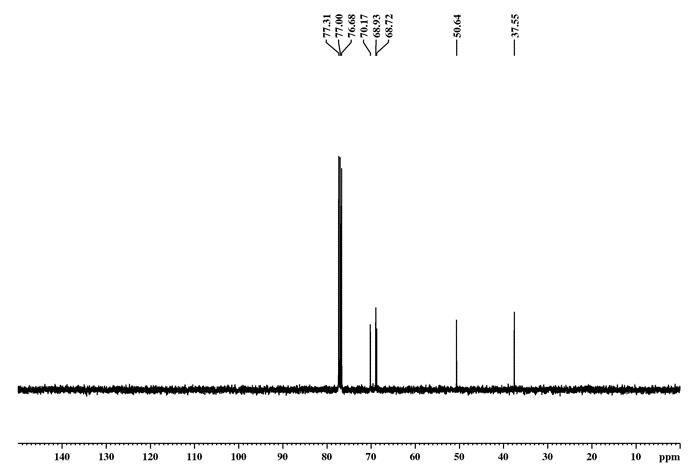


^13^C NMR spectrum of compound **S1a** (100 MHz, CDCl_3_)


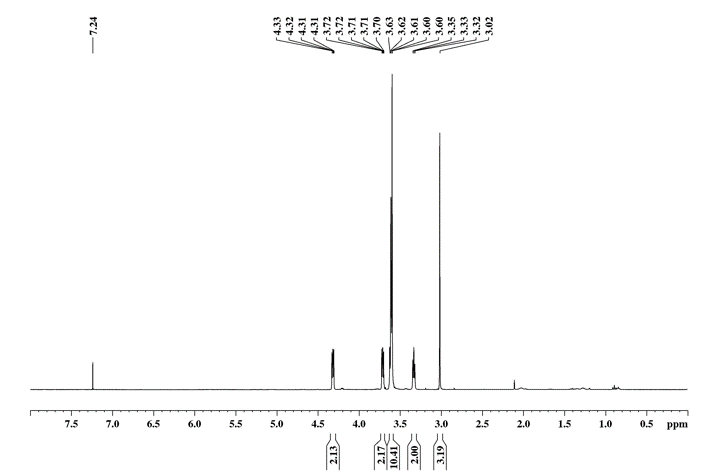


^1^H NMR spectrum of compound **S1b** (400 MHz, CDCl_3_)

^^
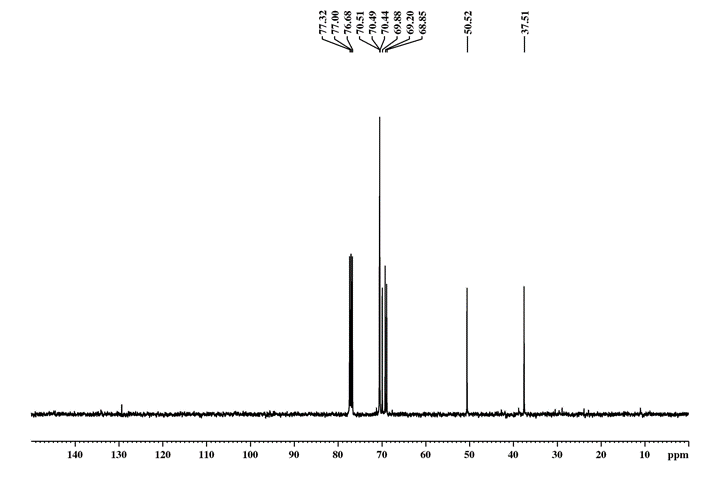


^13^C NMR spectrum of compound **S1b** (100 MHz, CDCl_3_)


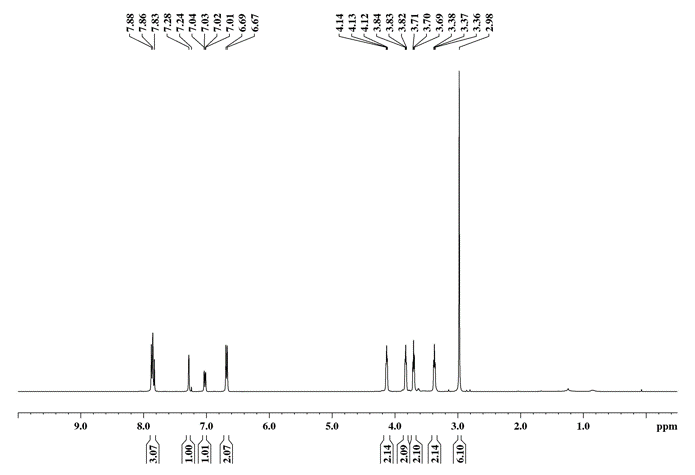


^1^H NMR spectrum of compound **S2a** (400 MHz, CDCl_3_)


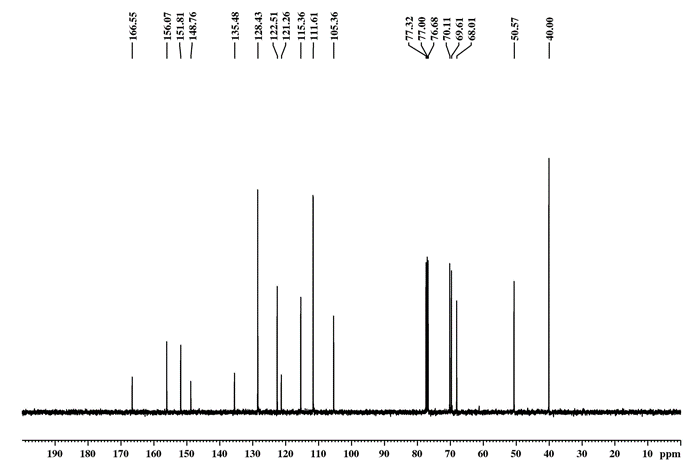


^13^C NMR spectrum of compound **S2a** (100 MHz, CDCl_3_)
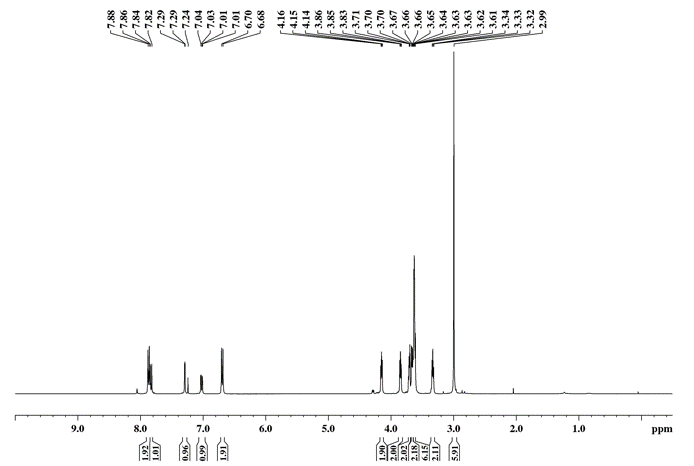


^1^H NMR spectrum of compound **S2b** (400 MHz, CDCl_3_)


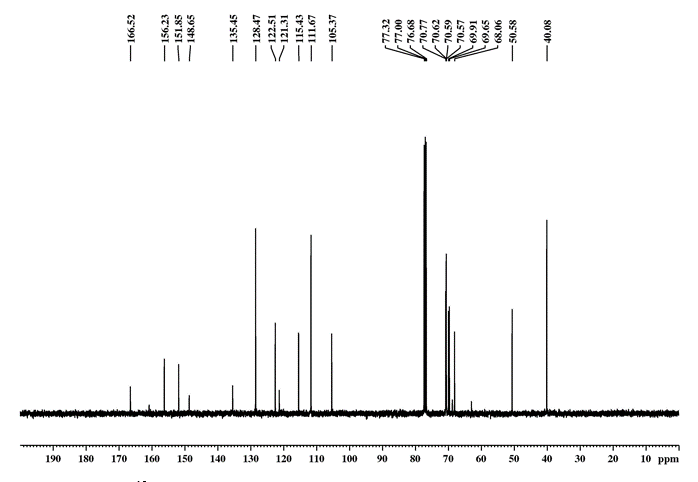


^13^C NMR spectrum of compound **S2b** (100 MHz, CDCl_3_)


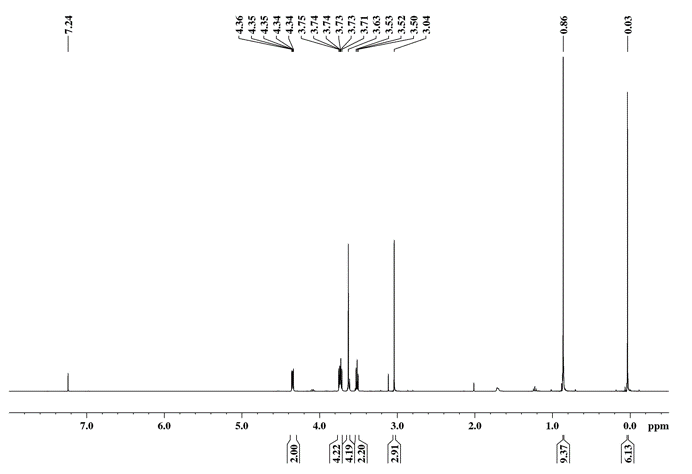


^1^H NMR spectrum of compound **S3a** (400 MHz, CDCl_3_)

^^
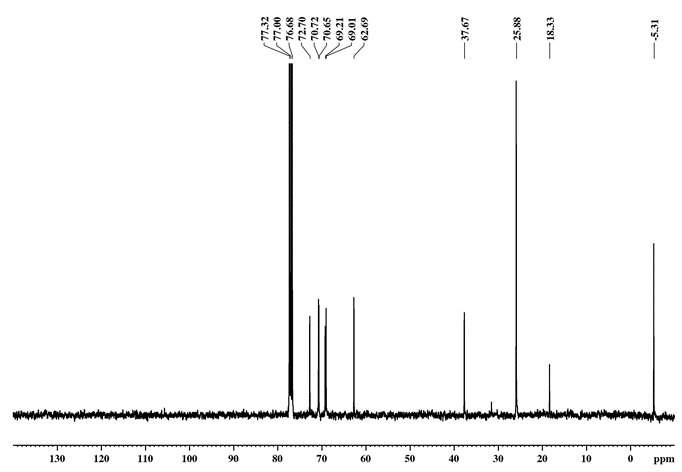


^13^C NMR spectrum of compound **S3a** (100 MHz, CDCl_3_)
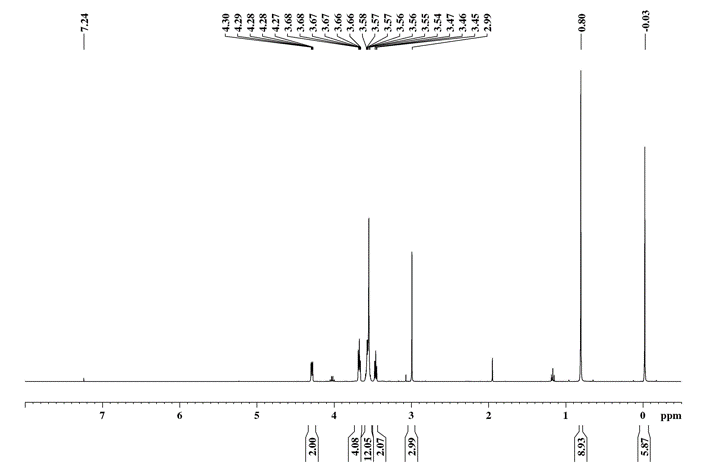


^1^H NMR spectrum of compound **S3b** (400 MHz, CDCl_3_)

^^
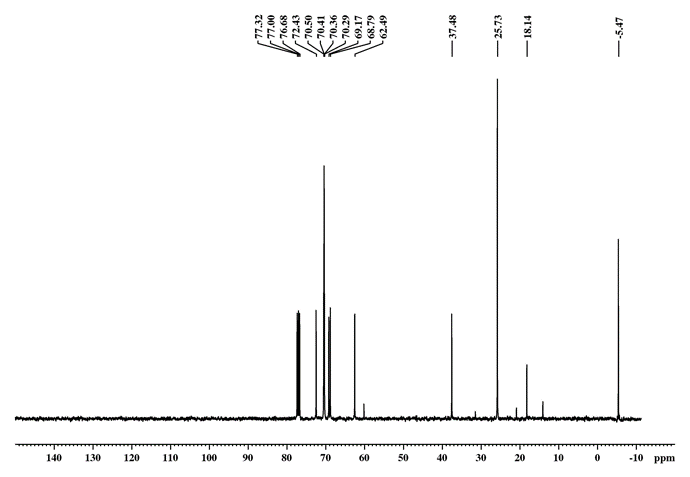


^13^C NMR spectrum of compound **S3b** (100 MHz, CDCl_3_)

^^
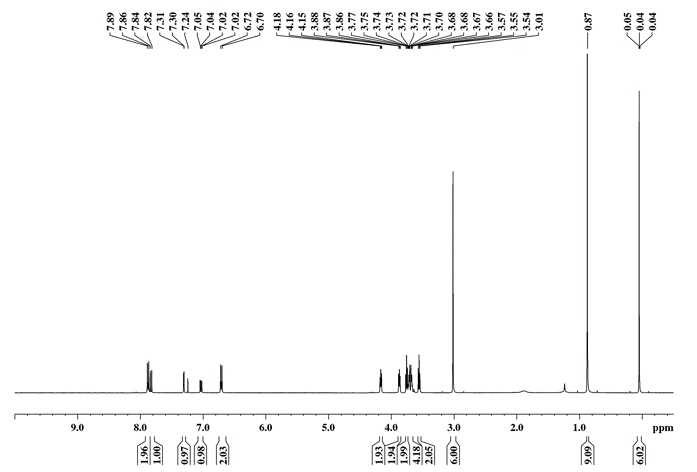


^1^H NMR spectrum of compound **S4a** (400 MHz, CDCl_3_)


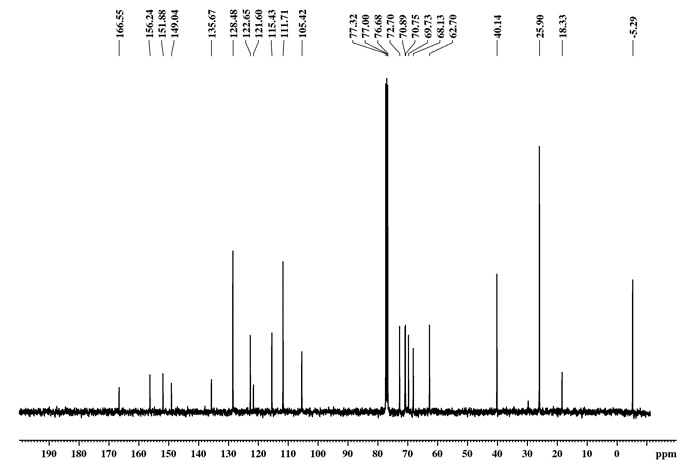


^13^C NMR spectrum of compound **S4a** (100 MHz, CDCl_3_)
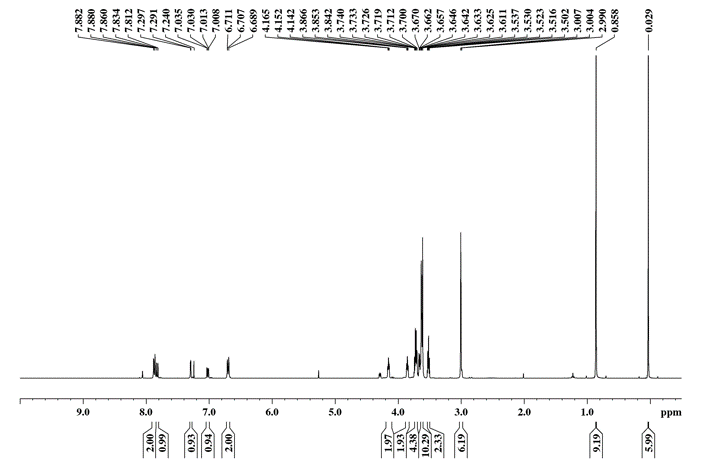


^1^H NMR spectrum of compound **S4b** (400 MHz, CDCl_3_)


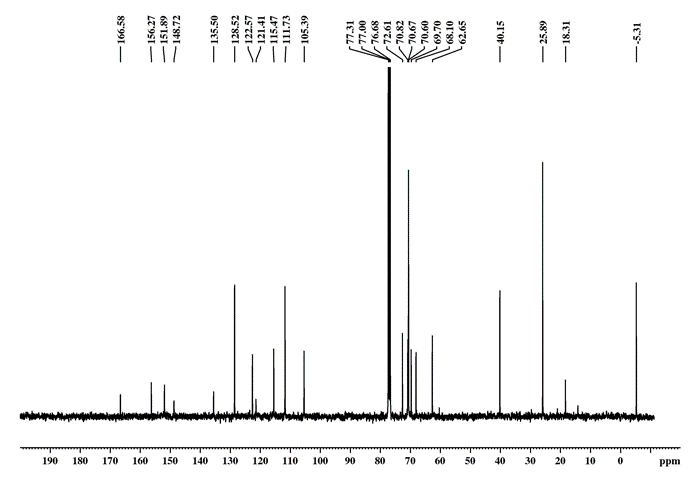


^13^C NMR spectrum of compound **S4b** (100 MHz, CDCl_3_)


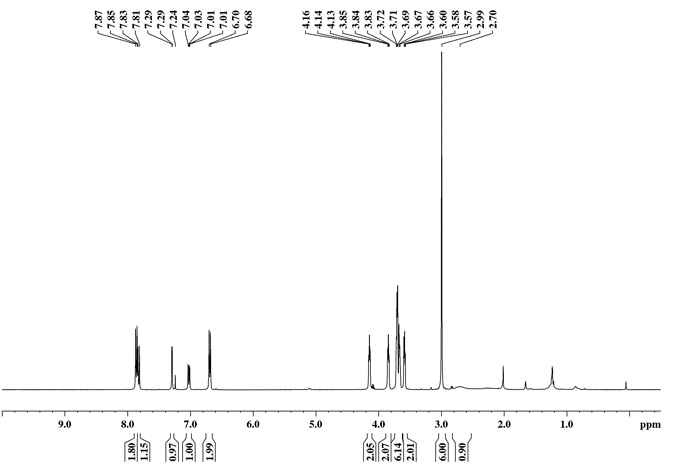


^1^H NMR spectrum of compound **S5a** (400 MHz, CDCl_3_)


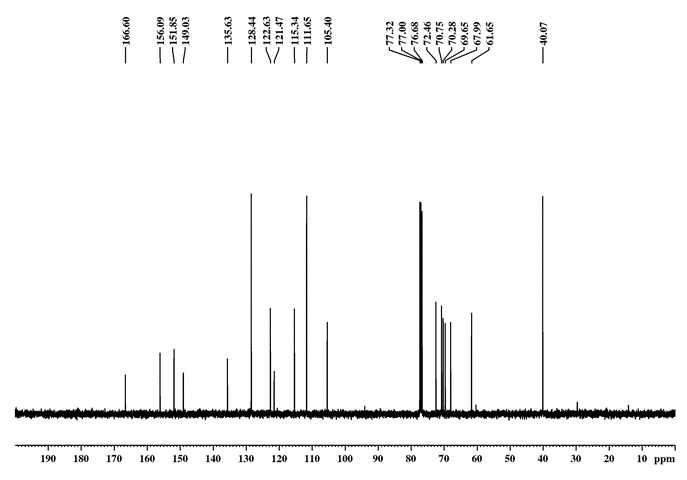


^13^C NMR spectrum of compound **S5a** (100 MHz, CDCl_3_)


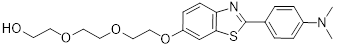

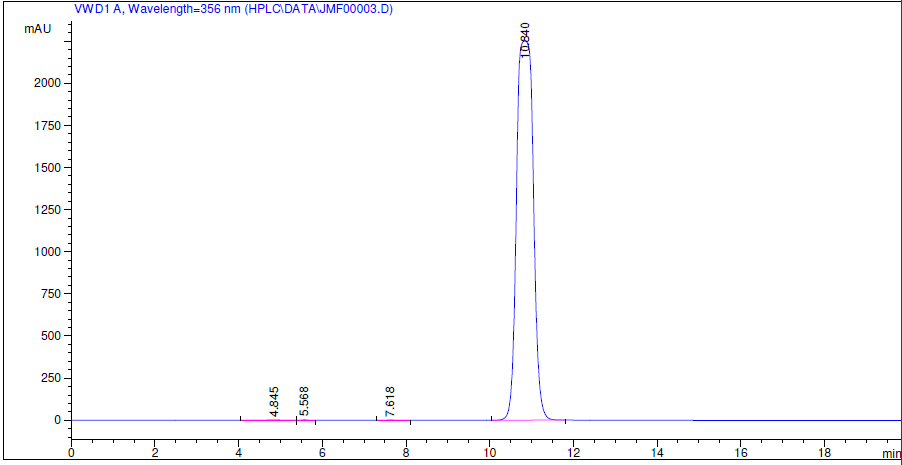


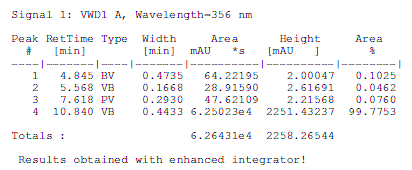


HPLC diagram of compound **S5a**. Silica column (Dikma, 10 × 250 mm, 10 μm particle size); elution: EtOAc/MeOH = 97:3 at a flow rate of 3.0 mL/min, *t*_R_ = 10.8 min, purity 99.8%.


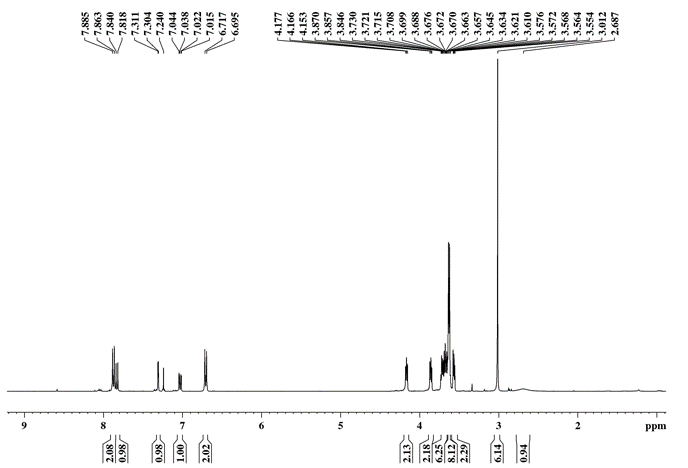


^1^H NMR spectrum of compound **S5b** (400 MHz, CDCl_3_)


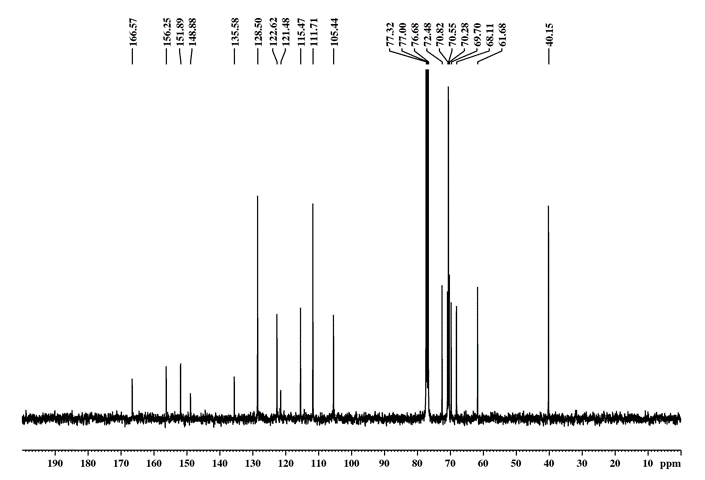


^13^C NMR spectrum of compound **S5b** (100 MHz, CDCl_3_)


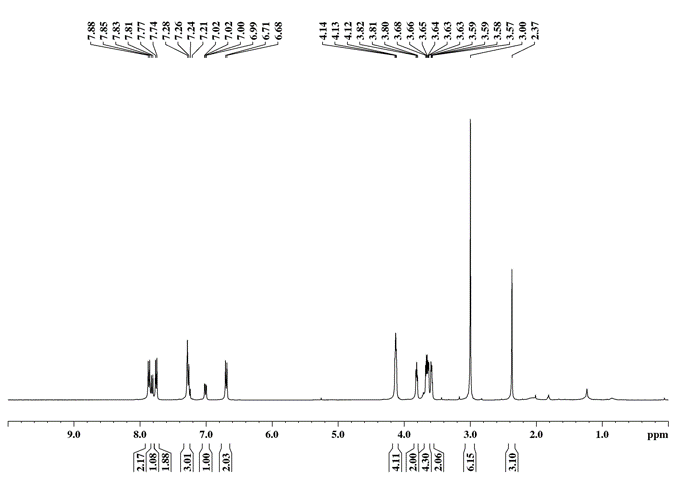


^1^H NMR spectrum of compound **S6a** (400 MHz, CDCl_3_)


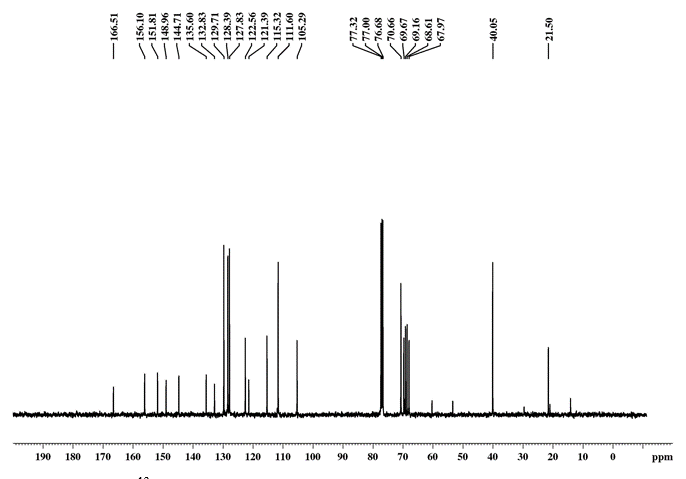


^13^C NMR spectrum of compound **S6a** (100 MHz, CDCl_3_)


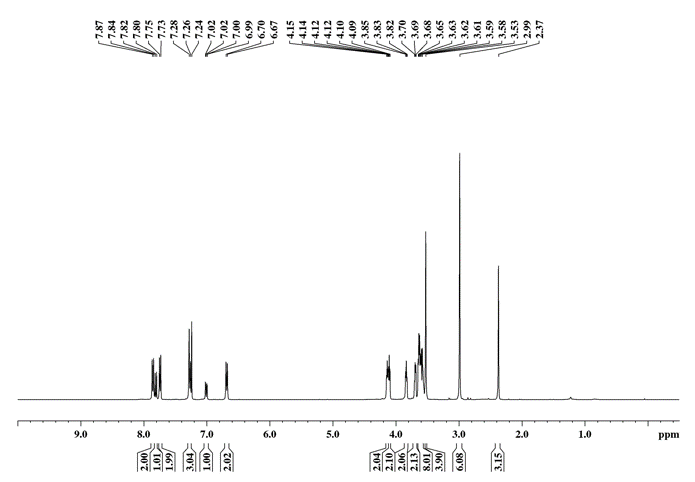


^1^H NMR spectrum of compound **S6b** (400 MHz, CDCl_3_)


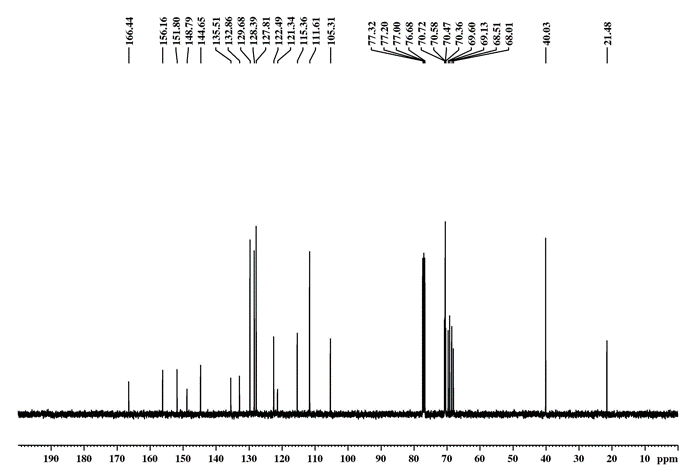


^13^C NMR spectrum of compound **S6b** (100 MHz, CDCl_3_)


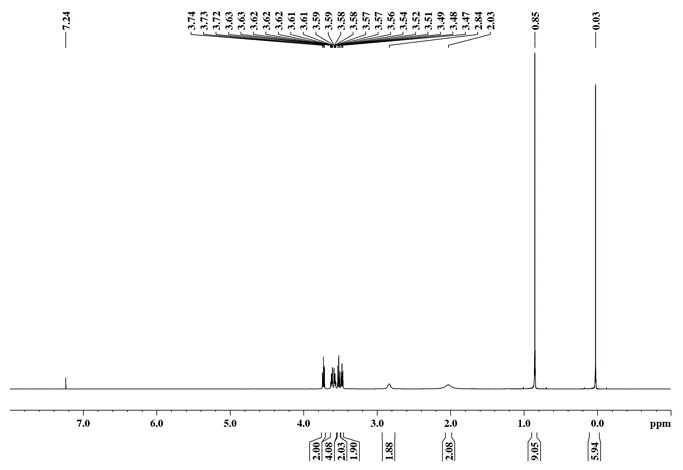


^1^H NMR spectrum of compound **S7** (400 MHz, CDCl_3_)


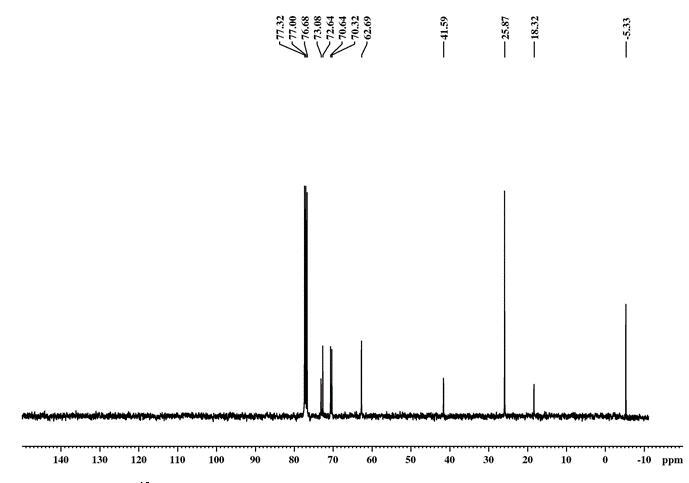


^13^C NMR spectrum of compound **S7** (100 MHz, CDCl_3_)


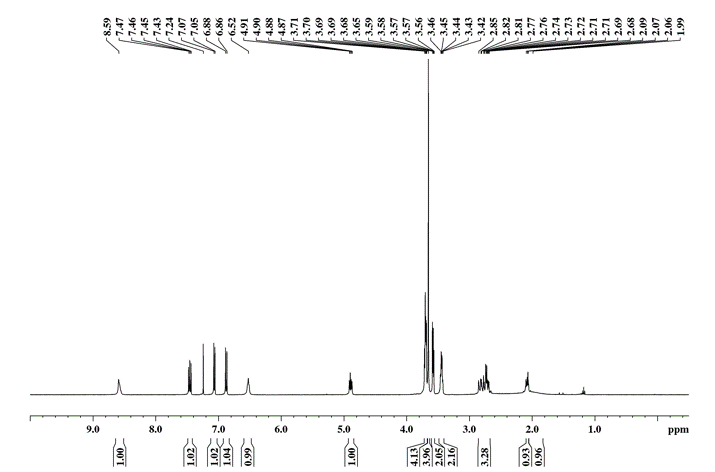


^1^H NMR spectrum of compound **S8** (400 MHz, CDCl_3_)


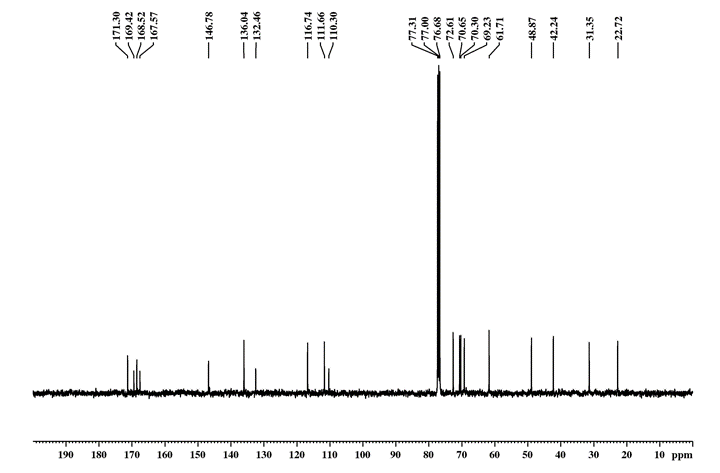


^13^C NMR spectrum of compound **S8** (100 MHz, CDCl_3_)


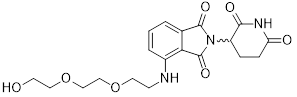

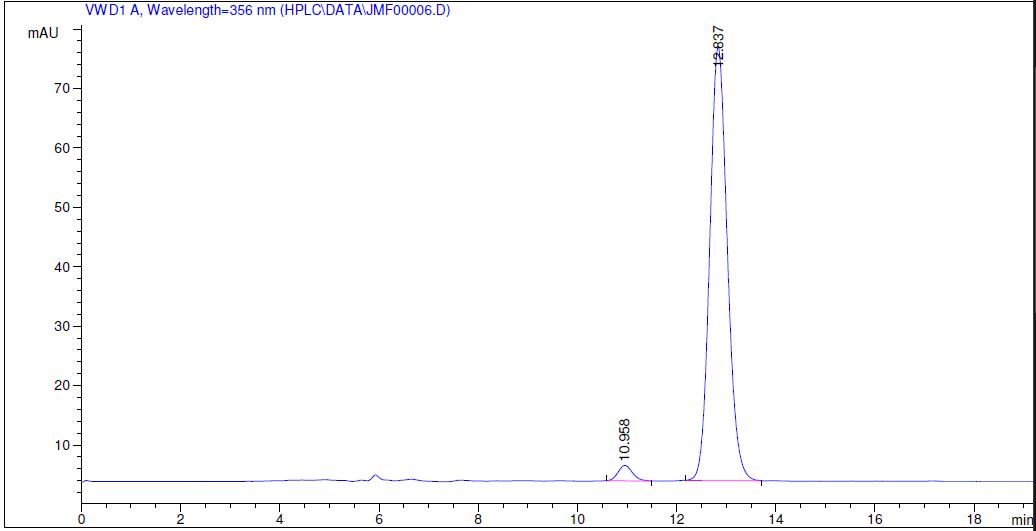


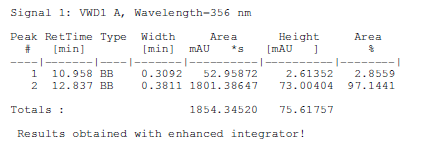


HPLC diagram of compound **S8**. Silica column (Dikma, 10 × 250 mm, 10 μm particle size); elution: EtOAc/MeOH = 97:3 at a flow rate of 3.0 mL/min, *t*_R_ = 12.8 min, purity 97.1%.
